# Bioorthogonal Reaction of *o*‐Quinone with Furan‐2(*3H*)‐One for Site‐Selective Tyrosine Conjugation and Construction of Bispecific Antibody Complexes

**DOI:** 10.1002/advs.202417260

**Published:** 2025-04-25

**Authors:** Hongfei Chen, Zhiyi Xu, Yishu Bao, Farshad Shiri, Dingdong Yuan, Yuke Hu, Biquan Li, Bin Zeng, Xiaojing Li, Hao Kong, Zikang Wang, Wilson Chun‐Yu Lau, Zhenyang Lin, Jiang Xia

**Affiliations:** ^1^ Department of Chemistry The Chinese University of Hong Kong Shatin Hong Kong SAR China; ^2^ Department of Chemistry Hong Kong University of Science and Technology Kowloon Hong Kong SAR China; ^3^ Department of Applied Biology and Chemical Technology The Hong Kong Polytechnic University Kowloon Hong Kong SAR China

**Keywords:** antibody conjugation, antibody‐dependent cellular cytotoxicity, bispecific antibody complex, o‐quinone, tyrosine reaction

## Abstract

Bioorthogonal reactions enable the chemical conjugation of functional moieties to native proteins and empower the development of new diagnostic tools and therapeutics. Through site‐selective reactions, therapeutic molecules can be conjugated with antibodies in a stoichiometry‐ and site‐controlled manner. Here, a one‐pot chemoenzymatic reaction is reported that preferentially modifies a terminal tyrosine of recombinant proteins, or tyrosine 296 in the Fc domain of selected human antibodies. This reaction combines tyrosinase‐catalyzed oxidation of phenol to *o*‐quinone, the bioorthogonal addition reaction of *o*‐quinone with an azide‐containing furan‐2(*3H*)‐one (**FuA**) moiety, and the subsequent azide click reactions. To this surprise, experimental evidence indicates that the *o*‐quinone−**FuA** reaction proceeds through nucleophilic addition instead of the cycloaddition pathway. This reaction enables site‐selective modification of therapeutic human antibodies, including atezolizumab, trastuzumab, daratumumab, and cetuximab. Monofunctionalized antibody conjugates and DNA‐templated bispecific antibody complexes (DNA‐bsAbC) are thus constructed in a modular way. DNA‐bsAbC acts as a bispecific engager to mediate the interaction between immune cells and cancer cells, resulting in antibody‐dependent cellular cytotoxicity (ADCC) toward cancer cells. Taken together, here a bioorthogonal reaction is reported for site‐selective tyrosine conjugation in recombinant proteins and human antibodies and showcase its application in constructing antibody conjugates for potential applications in immunotherapies.

## Graphical abstract

A site‐selective, efficient, and mild tyrosine conjugation via tyrosinase oxidation and nucleophilic addition, allowing for site‐selective functionalization of peptides, recombinant proteins, and IgGs. Site‐selective mono‐functionalization of IgGs at Y296 enables the construction of immunoliposomes. Site‐selective IgG‐DNA conjugates enable the construction of DNA‐templated antibody dimers for ADCC.

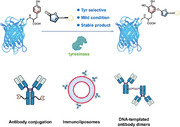

## 1 Introduction

Since the approval of the first monoclonal antibody (mAb), muromonab‐CD3, by the US Food and Drug Administration (FDA) in 1986 for the prevention of acute transplant rejection, antibody therapies have revolutionized modern medicine. Monoclonal antibody therapies provide targeted and effective treatments for diseases such as cancers, autoimmune disorders, and infectious diseases.^[^ ^1^, ^2^ ^]^ Besides monoclonal antibodies, antibody derivatives such as antibody‐drug conjugates (ADCs) and bispecific antibodies have been developed to enhance the cytotoxic effects and immune responses against cancer cells.^[^ ^3^, ^4^, ^5^, ^6^ ^]^ Bispecific antibodies are clinically used as bispecific engagers, which bridge immune effector cells and cancer cells for enhanced cytotoxicity.

With the increasing need of constructing new antibody conjugates, the field also requests more antibody functionalization strategies.^[^ ^5^, ^6^, ^7^, ^8^ ^]^ However, very few organic chemical reactions are compatible with antibody functionalization. In particular, modifying native, clinically used, and commercially available antibodies without genetic engineering is challenging.^[^ ^9^ ^]^ Such chemical reactions must proceed under mild conditions close to the physiological environment, i.e., in aqueous buffered solutions at neutral pH and room temperature.^[^ ^5^, ^6^, ^9^ ^]^ Ideally, these reactions also need to be highly chemo‐selective and site‐selective to minimize the risk of affecting antibody structure or activity. Among the 20 natural amino acids, cysteine‐specific reactions are the most popular due to the high nucleophilicity of the thiol group and cysteine's low abundance on the protein surface.^[^ ^10^ ^]^ As an immunoglobulin G (IgG) lacks surface‐exposed free cysteines, partial reduction of the disulfide bonds is required to unlock the interchain disulfide bonds to expose reactive cysteine residues.^[^ ^11^, ^12^, ^13^, ^14^ ^]^ Exposing IgG with reducing agents may significantly destabilize the antibody structure. Alternatively, antibody engineering is required to introduce cysteine residues. On the other hand, antibodies can be functionalized by amine‐reacting reagents such as N‐hydroxysuccinimide (NHS) esters, but it is hard to control the reaction sites and stoichiometry.^[^ ^15^ ^]^ In view of this limitation, we strived to harness proximity reactions to control the regioselectivity of cysteine or lysine reactions.^[^ ^16^, ^17^, ^18^, ^19^, ^20^, ^21^, ^22^, ^23^, ^24^ ^]^ Recently, utilizing an Fc‐binding peptide FcIII as the guiding binder, we achieved site‐selective functionalization of a single lysine residue (Lys 248) at the Fc domain of an antibody, proving that proximal reaction can achieve residue selectivity in antibody reactions.^[^ ^23^ ^]^

**Figure 1:**
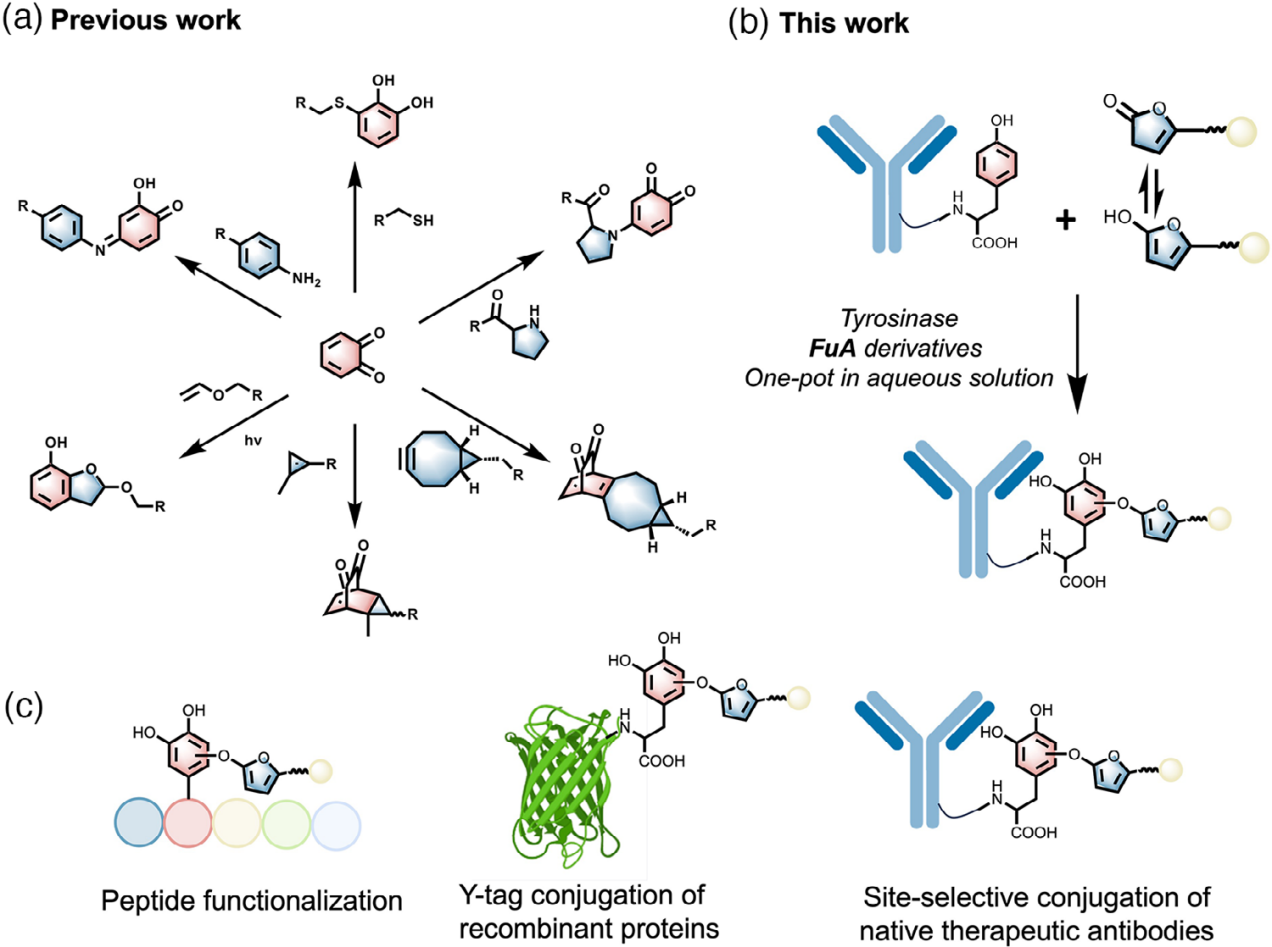
Tyr conjugation through the *o*‐quinone−**FuA** reaction. A) A summary of *o*‐quinone conjugation reactions. B) Scheme of the one‐pot tyrosine reaction combining tyrosinase oxidation and *o*‐quinone addition in the aqueous solution. C) Applications of the Tyr reaction peptides, recombinant proteins, and native therapeutic antibodies.

Tyrosine is an attractive target for protein reactions due to its relatively low abundance and rich chemistry.^[^ ^25^, ^26^ ^]^ Tyrosine reactions, such as the Mannich‐type reaction, the diazonium reaction, the sulfur fluoride exchange (SuFEx) reaction, the triazolinedione reaction, and the transition metal‐catalyzed reaction, have been widely used for protein modifications.^[^ ^27^, ^28^, ^29^, ^30^, ^31^, ^32^, ^33^, ^34^, ^35^, ^36^, ^37^, ^38^, ^39^, ^40^, ^41^ ^]^ New members of tyrosine reactions include electrochemical reactions with urazoles or phenothiazine derivatives, photocatalytic reactions based on dinitroimidazole reagent, and lumiflavin‐catalyzed formyl group conjugation.^[^ ^42^, ^43^, ^44^, ^45^, ^46^, ^47^, ^48^, ^49^ ^]^ Interestingly, tyrosinases, such as the tyrosinase from the mushroom *Agaricus bisporus (A. bisporus)*, can oxidize phenols to *o*‐quinone. *o*‐Quinone can undergo a variety of reactions, such as bioconjugation with cysteine or amine, or strain‐promoted [4 + 2] cycloaddition (SPOCQ) with cyclic alkynes such as bicyclo[6.1.0]nonyne (BCN) derivatives, etc. (**Figure** **1**a)^[^ ^50^, ^51^, ^52^, ^53^, ^54^ ^]^ For example, we recently proved the bioorthogonality of the *o*‐quinone−vinyl ether photoaddition reaction, which enabled us to develop chemoenzymatic tyrosine reactions. The mushroom tyrosinase from the commercial source showed a surprising site preference for tyrosine 296 (Tyr 296, or Y296) of the Fc domain. Combining these features, we reported a chemoenzymatic photoaddition reaction of IgG preferentially at Tyr 296.^[^ ^24^ ^]^

On another note, five‐membered lactone rings have unique reactivities.^[^ ^55^, ^56^ ^]^ The unsaturated β, γ‐butenolide, 5‐substituted‐furan‐2(3H)‐one, can be deprotonated at the α‐position, yielding a dienolate that can be involved in reactions such as Michael reactions, Mannich reactions, and aldol reactions.^[^ ^57^, ^58^, ^59^ ^]^ This feature was utilized in transformations involving nucleophilic reactivity from the α‐ or γ‐position.^[^ ^60^, ^61^, ^62^, ^63^ ^]^ Acting as an electrophile, *o*‐quinone was reported to react with furan‐2(*3H*)‐ones using Takemoto's catalyst to perform a vinylogous aldol reaction.^[^ ^64^ ^]^ *o*‐Quinone and furan‐2(3*H*)‐one reaction also took place in aqueous solutions without catalysts. For example, Zhang and coworkers reported anionic cycloaddend‐promoted bioorthogonal cycloaddition reactions between phenanthrene‐9,10‐dione and 5‐substituted‐furan‐2(3*H*)‐one derivative (**FuA**), which proceeded rapidly in an aqueous solution and on live cells.^[^ ^65^ ^]^

We envision that the ionic enolate isoform of furan‐2(*3H*)‐one may react with *o*‐quinone through cycloaddition or nucleophilic addition similar to the reaction between *o*‐quinone and thiols (Figure 1B). In the context of tyrosine reaction, because thiol‐containing compounds inhibit tyrosinase activity,^[^ ^50^ ^]^ here, we first prove that *o*‐quinone reaction with furan‐2(*3H*)‐one is biocompatible, and show that the combination of tyrosinase oxidation with *o*‐quinone reaction with furan‐2(*3H*)‐one enables site‐selective tyrosine functionalization in proteins. Surprisingly, we have found that *o*‐quinone undergoes enol‐mediated nucleophilic addition reaction with furan‐2(*3H*)‐one in the aqueous buffer, rather than the previously reported Diels‐Alder (DA) cycloaddition reaction. This reaction is analogous to the quinone‐thiol conjugation reaction, which forms a C‐S bond.^[^ ^50^, ^66^ ^]^ Similar addition reactions were also found in biochemical pathways, such as the aromatic ring metabolism,^[^ ^67^ ^]^ mussel adhesion,^[^ ^68^ ^]^ and wine aging.^[^ ^69^ ^]^ This quinone‐enol addition reaction yields a stable C─O bond in proteins, which allows us to realize site‐selective antibody modification at Y296 and enables the construction of antibody conjugates for targeted drug delivery and immunotherapies (Figure 1C).

## 2 Results and Discussion

### 2.1 Reaction of Furan‐2(*3H*)‐One and *o*‐Quinone

**Figure 2:**
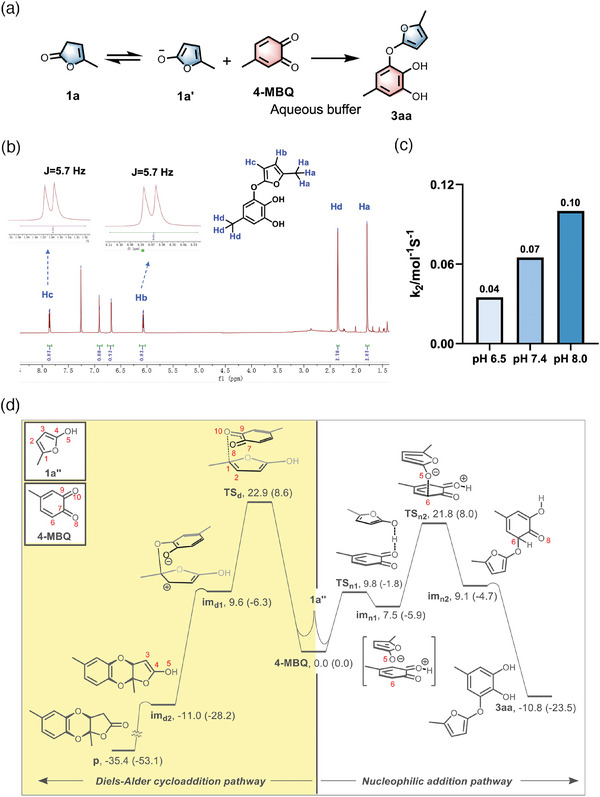
A model reaction between **FuA** and **4‐MBQ**. A) Isomerization of **1a** followed by the addition of **4‐MBQ** in an aqueous solution yields **3aa**. Reaction condition: **1a** (0.1 mmol), **4‐MBQ** (0.5 mmol) were dissolved in PB buffer (pH 6.5, 0.2 m). The reaction system was stirred at room temperature for 30 min. B) ^1^H‐NMR spectra of product **3aa** showing the presence of a dihydroxyphenyl structure. C) Reaction kinetic rates of the model reaction in the aqueous solution at different pH values. D) Comparison of energy profiles calculated using DFT Theory for the nucleophilic addition pathway and the Diels–Alder cycloaddition pathway.

We start our exploration of the reaction of furan‐2(*3H*)‐one (**FuA**) with the diketone moiety of the *o*‐quinone from a model reaction of **1a** and 4‐methyl‐1,2‐benzoquinon (**4‐MBQ)** (**Figure** **2**A). The two compounds react in a phosphate buffer (0.2 m, pH 6.5) under the room temperature for 30 min to give an adduct. ^1^H‐NMR and ^1^H‐^1^H COSY NMR spectra analysis revealed a dihydroxyphenyl core structure in the product, consistent with a nucleophilic addition product **3aa** (Figure 2B; Figure S1, Supporting Information). To our surprise, the reaction did not give the cycloaddition product as previously reported.^[^ ^65^ ^]^ We propose that the reaction proceeds through a nucleophilic addition in the aqueous solution: **1a** first undergoes isomerization to generate the enolate **1a′**, which then conjugates with **4‐MBQ** to form the final product **3aa**. This mechanism is consistent with the observation that higher pH expedites the reaction rate (Figure 2C; Figure S2, Supporting Information).

To gain insights into the addition reaction between **1a“** and **4‐MBQ**, we utilized density functional theory (DFT) calculations at the SMD/wb97xd/def2‐TZVP//SMD/wb97xd/6‐31G(d) level of theory. Our DFT calculations focused on the comparison of the Diels‐Alder (DA) cycloaddition and nucleophilic addition pathways. The DFT results indicate that the DA cycloaddition proceeds through an asynchronous stepwise pathway, which begins with the interaction between C1 of **1a”** and O10 of **4‐MBQ**, resulting in the formation of intermediate **im_d1_**. The elongation of the C1─C2 bond in **im_d1_** (1.514 Å) when compared to **1a“** (1.354 Å) indicates a substantial reduction in the bond order of the C1─C2 bond. Thus, a formal carbocationic C2 center is assigned in **im_d1_**. From **im_d1_**, an almost barrierless C–O coupling between the carbocationic C2 center and the negatively charged O8 gives **im_d2_**. Finally, a 1,3‐proton transfer from O5 to C3 in **im_d2_** yields the final DA cycloaddition product **p**. In contrast, the nucleophilic addition pathway begins with a proton migration from **1a”** to **4‐MBQ**, which gives intermediate **im_n1_**, via the transition state **TS_n1_** overcoming a barrier of 9.8 kcal mol^−1^. The resulting highly reactive intermediate **im_n1_** readily undergoes nucleophilic addition on the C6 carbon of the protonated **4‐MBQ**, forming **im_n2_**. A subsequent 1,3‐proton transfer in **im_n2_** leads to the final nucleophilic addition product **3aa**. Our calculations identify the nucleophilic addition step as the rate‐determining step for this pathway. Consistent with the experimental observation, the DFT results shown in Figure 2D indicate that the transition state **TS_n2_** is lower in energy than **TS_d_**, suggesting a kinetic preference for the formation of nucleophilic addition product **3aa** over that of DA cycloaddition product **p**.

The preference for the nucleophilic addition pathway over the DA cycloaddition pathway can be attributed to the presence of a large non‐aromatic conjugated system in **4‐MBQ**. The extended conjugation greatly facilitates the nucleophilic addition. It is worth noting that when phenanthrenedione is used (instead of **4‐MBQ**), a nucleophilic addition would lead to dearomatization, which is expected to be very energy‐demanding. In other words, the lack of suitable sites in phenanthrenedione for nucleophilic addition renders this pathway highly unfavorable. This observation aligns perfectly with the exclusive occurrence of the DA cycloaddition pathway when phenanthrenedione is used.^[^ ^65^ ^]^

### 2.2 Tyrosine Reaction of Peptides and Recombinant Proteins

**Figure 3:**
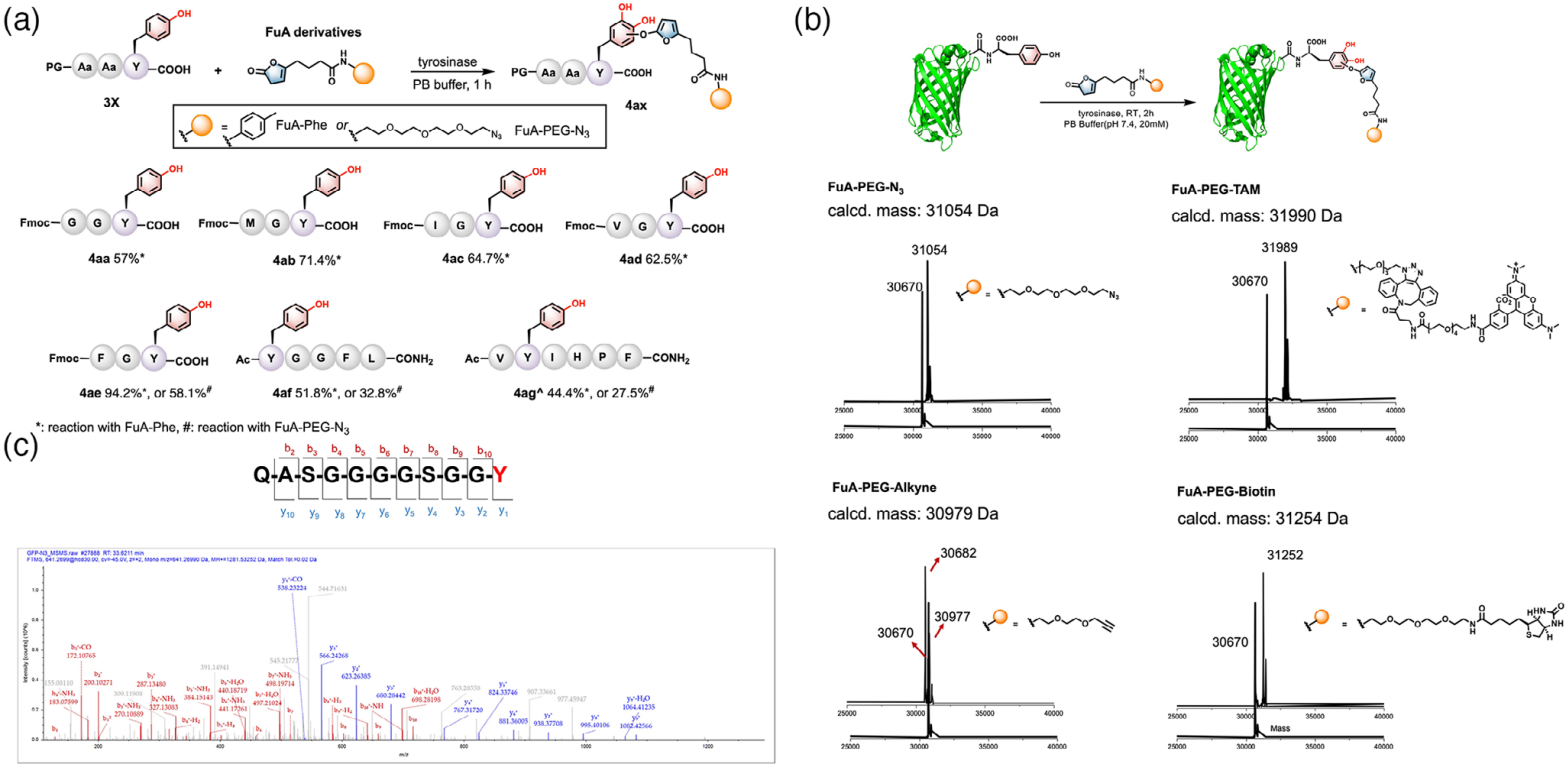
Tyr reactions of synthetic peptides and recombinant proteins. A) One‐pot chemoenzymatic reactions of tyrosine‐containing peptides with a **FuA** derivative **2a**. ^: 12 h reaction time for **4ag**. B) MALDI‐TOF analysis of the conjugation of **FuA‐PEG‐N_3_**, **FuA‐PEG‐TAM**, **FuA‐PEG‐Alkyne**, and **FuA‐PEG‐biotin** to GFP‐GGY. C) LC‐MS/MS analysis of the reaction site of conjugation product between **FuA‐PEG‐N_3_** and GFP‐GGY revealing the modification of the C‐terminal Y‐tag.

Next, we explored combining the tyrosinase oxidation reaction and the *o*‐quinone addition reaction in synthetic peptides with a C‐terminal tyrosine. Fmoc‐GGY‐OH (G: Gly, Y: Tyr, at 10 mm) was incubated with tyrosinase (1.68 µm) and **FuA‐Phe** (100 mm) in the phosphate buffer (0.2 m, pH 6.5) at room temperature (**Figure** **3**A). Fmoc‐GGY‐OH peptide shifted to a new peak in the HPLC chromatogram (Figure S4, Supporting Information). The conjugation product showed high stability in both acidic and basic solutions (Figure S3, Supporting Information). All the Tyr‐containing peptides can react, albeit with different yields (Figures S4–S10, Supporting Information). The presence of methionine, isoleucine, phenylalanine, or other amino acids did not affect the reaction. We observed that the chemoenzymatic reaction highly favors C‐terminal Y. The N‐terminal or internal Y residues have a much lower chance of being modified. For example, the reaction of peptide **4ag** is exceptionally slow. This proves that the accessibility of Y to tyrosinase is the key factor in determining the site‐selectivity. Thus, the C‐terminal GGY sequence is the most preferred substrate of the chemoenzymatic reaction. Furthermore, when **FuA‐PEG‐N_3_** was used as the substrate, duplet peaks of products of identical m/z values were observed in the HPLC traces, suggesting the generation of configurational isomers (Figures S11–S13, Supporting Information). The **FuA** addition reaction may take place at two sites of the benzoquinone, leading to the formation of two steric isomers (Figure S14, Supporting Information). The formation of two isomeric products, however, was not observed in the model reaction between **FuA** and **4‐MBQ** (Figure 2), possibly because the methyl group in **4‐MBQ** dictated the regioselectivity of the addition reaction in the context of the small molecule while such the difference of these two nucleophilic sites was negligible in tyrosine residues in the context of a peptide.

Next, we explored the reaction of a green fluorescent protein (GFP) with a C‐terminal GGY sequence (called Y‐tag here). Briefly, GFP‐GGY (1 *eq*.) was incubated with tyrosinase (0.01 *eq*.) along with various **FuA** derivatives (100 *eq*.) in PB buffer (pH 7.4) at room temperature for 1 h. LC‐MS analysis of **FuA‐PEG‐N_3_** conjugated GFP‐GGY revealed an increase in molecular weight, indicating the successful addition reaction. Subsequent LC‐MS/MS analysis confirmed the C‐terminal tyrosine as the reaction site (Figure 3B; Figures S15 and S16, and S20, Supporting Information). Similarly, **FuA‐PEG‐tetramethylrhodamine (TAM)**, **FuA‐PEG‐Alkyne**, and **FuA‐PEG‐biotin** were successfully conjugated to GFP‐GGY, as confirmed by LC‐MS analysis (Figures S17–S19, Supporting Information). Next, we fused the Y‐tag at the C‐terminus of a HER2‐specific nanobody, resulting in HER2‐nanobody‐GGY, and showed that the nanobody‐GGY could be biotinylated by **FuA‐PEG‐biotin**. Moreover, mutating the C‐terminal Y in the Y‐tag to Phe (F) abolished the biotinylation reaction (Figure S21, Supporting Information).

We observed no appreciable level of Tyr‐specific reaction was observed in HER2‐nanobody‐GGF, despite that it contains six internal tyrosine residues (Figure S21, Supporting Information). These data demonstrate that the chemoenzymatic *o*‐quinone reaction is compatible with proteins under physiological conditions and highly prefers solvent‐exposed tyrosine residues, such as C‐terminal GGY.

### 2.3 Site‐Selective Tyr Reaction of IgG

**Figure 4:**
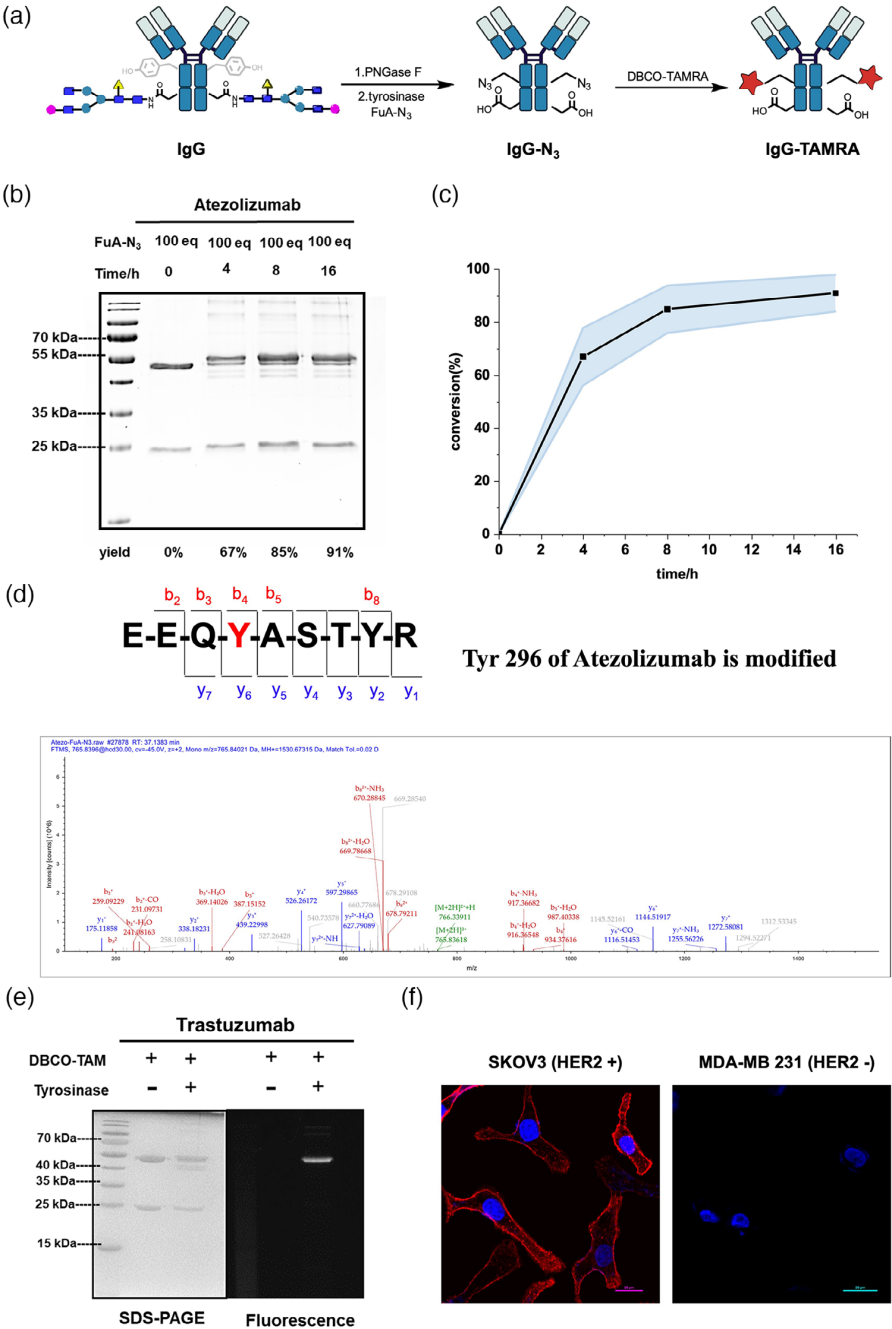
Tyr conjugation of native therapeutic antibodies. A) Schematic illustration showing the procedure of antibody modification by **FuA‐PEG‐N_3_**. B) SDS‐PAGE analysis of the site‐selective Tyr reaction of Atezo. Briefly, a solution containing 5.0 µm IgG, 500 µm **FuA‐PEG‐N_3_**, and 2.0 µm tyrosinase in PB buffer (0.2 m, pH 7.4) was incubated at 4 °C for 0–16 h. Small molecules were removed by centrifugation and DBCO‐TAMRA was added. The reaction solution was quenched by 1% sodium dodecyl‐sulfate (SDS), resolved by denaturing SDS−polyacrylamide gel electrophoresis (SDS‐PAGE), imaged by Coomassie blue staining, and quantified. C) Quantification of conjugation conversion with different incubation times. D) LC‐MS/MS analysis of Atezo‐N_3_ providing evidence of Y296 as the reaction site. E) SDS‐PAGE analysis of Tras‐TAMRA showing fluorescent labeling only on the heavy chain. Left, Coomassie‐stained image; right, fluorescent image at an excitation wavelength of 365 nm. F) Fluorescent images of SKOV3 cells and MDA‐MB 231 cells incubated with Tras‐TAMRA, respectively. Scale bar, 20 µm.

The mushroom tyrosinase is known to preferentially oxidize Y296 of selected commercially available antibodies to *o*‐quinone,^[^ ^24^ ^]^ so we tested the reaction of atezolizumab (Tecentriq, or called Atezo here), a monoclonal antibody used to treat breast and lung cancers (**Figure** **4**A). We incubated atezolizumab with **FuA‐PEG‐N_3_** (100 *eq*.) and tyrosinase (0.4 *eq*.) at 4 °C for 16 h. Then we performed a click reaction to attach a fluorophore, 6‐carboxy tetramethylrhodamine (TAMRA) via the azide−dibenzocyclooctyne (DBCO) reaction. On SDS‐PAGE, we observed a molecular weight increase in the heavy chain but not the light chain (Figure 4B; Figure S22, Supporting Information). Quantification showed up to 90% of the heavy chain reacted in 16 h (Figure 4C). LC‐MS/MS analysis of the digested fragments of the labeled Atezo gave evidence that Y296 was modified (Figure 4D; Figure S23–S25, Supporting Information), consistent with the previous report.^[^ ^24^ ^]^ Although we could not rule out the possibility that other residues might also be the reaction sites, these data suggest that Y296 is most likely the preferred reactive site. This reaction was also successfully applied to other therapeutic antibodies, trastuzumab (Tras), daratumumab, and cetuximab. For Tras, the antibody was pretreated with PNGase F enzyme to remove the N297 glycan, exposing Y296 in the Fc domain.^[^ ^70^, ^71^ ^]^ Deglycosylated Tras was then successfully labeled (Figure 4E; Figure S23 and S24, Supporting Information). Without deglycosylation, the antibody cannot be labeled, suggesting the reaction prefers Y296 which is adjacent to N297. The resultant Tras‐TAMRA conjugate maintained its binding ability with HER2‐positive SKOV3 cells but did not bind to HER2‐negative MDA‐MB‐231 cells (Figure 4F; Figure S26, Supporting Information). Notably, we observed that increasing the concentration of tyrosinase or prolonging the reaction time might lead to reactions at other tyrosine residues in the Fc domain or the Fab domain (data not shown). These data show that the chemoenzymatic addition reaction preferred Y296 of therapeutic antibodies.

### 2.4 Construction of Immunoliposome via Antibody Lipidation

**Figure 5:**
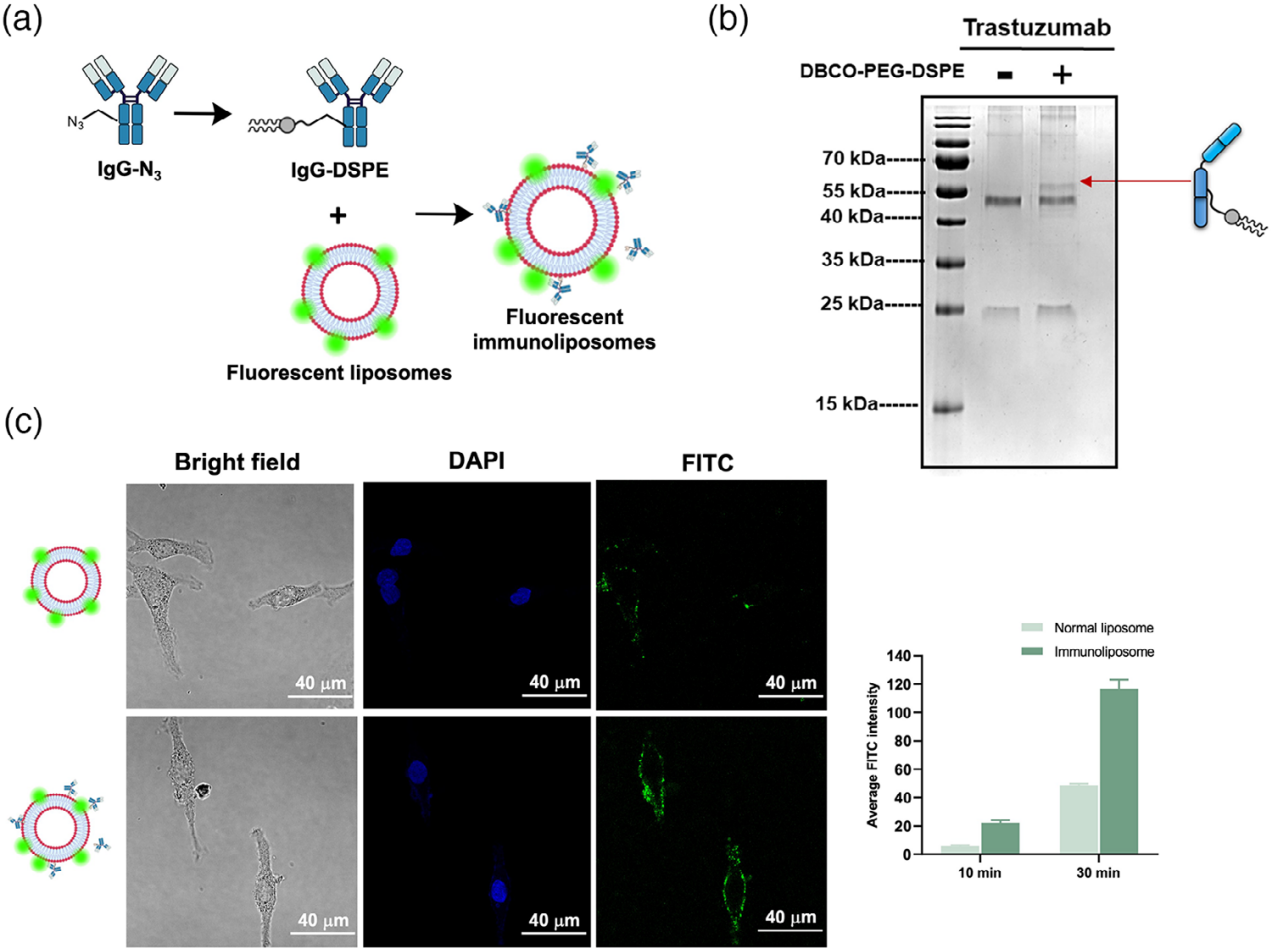
Construction of immunoliposomes and the enhanced binding interaction with HER2+ cells. A) Schematic illustration showing the synthesis of the antibody−lipid conjugate IgG‐DSPE and the construction of fluorescent immunoliposomes. B) SDS‐PAGE analysis showing the synthesis of Tras‐lipid conjugates. Briefly, Tras‐N_3_ (10 µm) and DSPE−PEG2000−DBCO (100 µm) were mixed in PBS buffer (pH 7.4) at 37 °C for 2 h. The reaction solution was quenched by 1% sodium dodecyl‐sulfate (SDS), resolved by denaturing SDS−polyacrylamide gel electrophoresis (SDS‐PAGE), and imaged by Coomassie blue staining. C) Confocal fluorescent microscope images showing the enhanced binding of liposomes with HER2+ SKOV3 cells, compared to liposomes without antibodies on the surface. Briefly, cells were incubated with fluorescent liposomes or immunoliposomes (3.3 µm) in a DMEM medium at 37 °C for 30 min, fixed, imaged (green showing the FITC signal), and quantified. The quantification was based on the original images shown in Figure S22 (Supporting Information) by ImageJ. Data are presented as the mean ± S.D. of n = 3 independent experiments.

Next, we synthesized antibody−lipid conjugates and subsequently functionalized liposomes to form construct antibody‐functionalized liposomes, also known as immunoliposomes (**Figure** **5**A).^[^ ^72^, ^73^ ^]^ Briefly, Tras‐N_3_ was incubated with 1,2‐distearoyl‐sn‐glycero‐3‐phosphoethanolamine (DSPE)−PEG2000−DBCO (10 *eq*.) for 2 h at 37 °C to give Tras‐DSPE. According to the SDS‐PAGE images, 16% of Tras was modified by the lipid molecule (Figure 5B, based on ImageJ grayscale analysis). Fluorescently labeled liposome was prepared by POPC (1‐palmitoyl‐2‐oleoyl‐glycero‐3‐phosphocholine), cholesterol, and a fluorescent lipid DSPE‐PEG2000‐FITC. Immunoliposome preparation was then generated by incubating Tras‐DSPE with the liposome for 30 min at 40 °C. The immunoliposome can enter HER2‐positive (HER2+) SKOV3 cells at 37 °C more efficiently than liposomes without surface‐conjugated Tras (Figure 5C; Figure S27, Supporting Information).

### 2.5 DNA‐Templated Bispecific Antibody Complexes (DNA‐bsAbCs)

**Figure 6:**
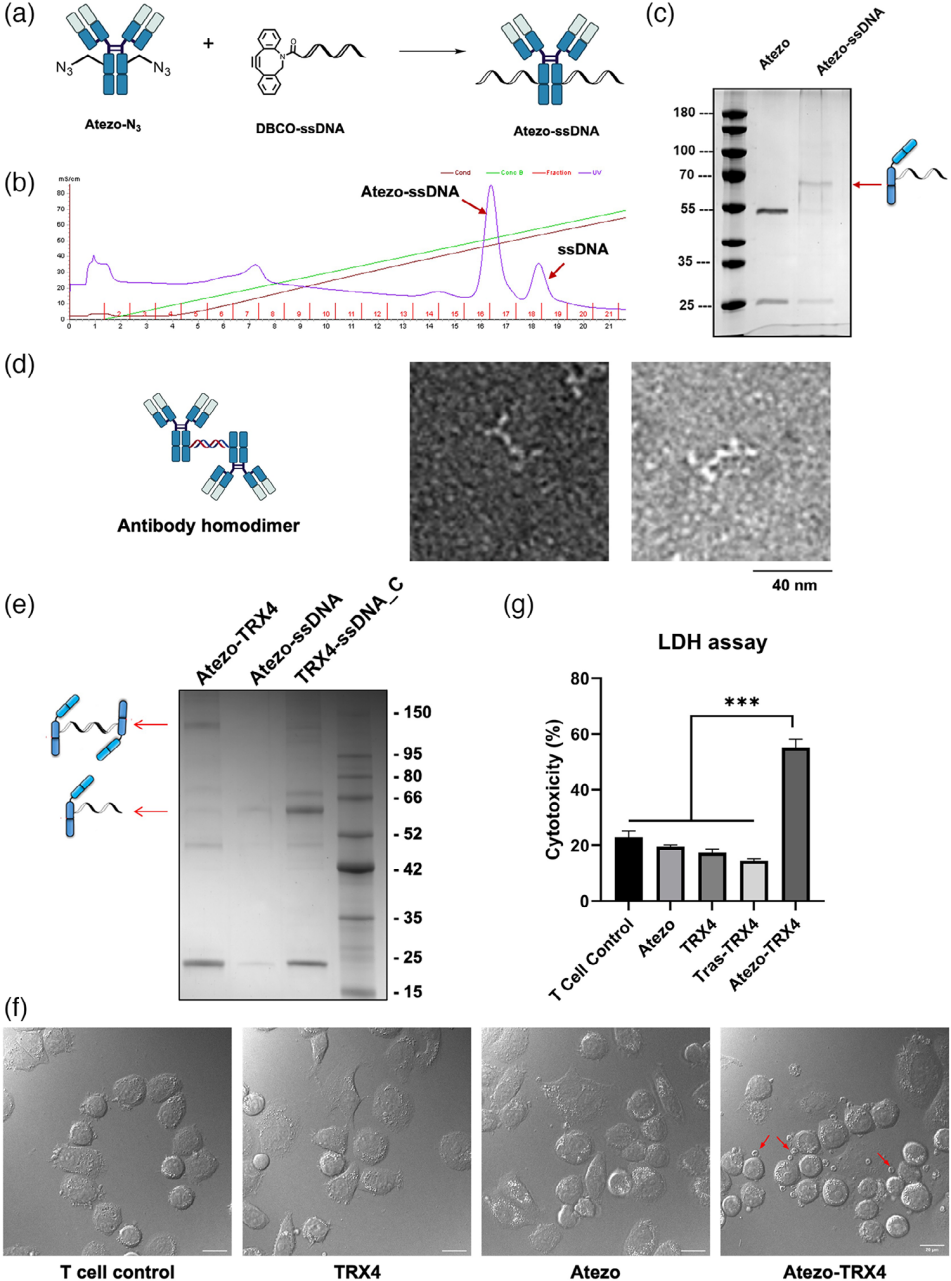
Construction of DNA‐templated antibody dimers for ADCC. A) Schematic illustration to show the construction of antibody‐ssDNA conjugates. B) FPLC spectrum of the reaction solution of antibody‐ssDNA conjugation. C) SDS‐PAGE images showing the purified Atezo‐ssDNA conjugates. D) Representative negatively stained EM micrographs showing the presence of antibody homodimers. Scale bar, 40 nm. E) SDS‐PAGE image of the DNA‐templated Atezo‐TRX4 heterodimer under the reducing condition. F) Confocal images showing that Atezo‐TRX4 heterodimer engages T cells to MDA‐MB‐231 cells. T cells were amplified from human PBMCs. The red arrows indicate T cells that attach to MDA‐MB‐231 cells after washing. G) Cytotoxicity of MDA‐MB‐231 cells in the presence of T cells and Atezo‐TRX4 heterodimer or antibodies alone. Data are presented as the mean ± S.D. of n = 3 independent experiments; ^***^, *P* < 0.001, according to unpaired Student's t‐test.

Lastly, we synthesized antibody‐DNA conjugates and constructed antibody dimers through DNA hybridization (**Figure** **6**A).^[^ ^74^, ^75^, ^76^ ^]^ Briefly, Atezo‐N_3_ was first generated by reacting Atezo with **FuA‐PEG‐N_3_**. After desalting, Atezo‐N_3_ (3 mg mL^−1^) was mixed with a DBCO‐conjugated single‐strand DNA, DBCO‐ssDNA (MW 7 kDa), at a ratio of 1:10 at room temperature for 16 h. According to the SDS‐PAGE, the click reaction proceeded and gave a band of over 63 kDa under the reducing condition, corresponding to the molecular weight of the Atezo‐ssDNA product with a yield of ≈ 60% (Figure 6B,C, calculated based on the FPLC analysis). Then, DNA‐templated Atezo‐Atezo dimer was synthesized by incubating Atezo conjugated to two complementary ssDNA sequences, Atezo‐ssDNA and Atezo‐ssDNA‐C, with a 1:1 ratio at 37 °C. Under a negative stain electron microscope (EM), we observed the dimeric antibody structure (Figure 6D; Figure S28, Supporting Information).

### 2.6 Effector‐Cell‐Mediated Cytotoxicity of DNA‐bsAbCs

To produce a bispecific engager capable of recruiting cytotoxic T lymphocytes to specifically target PD‐L1+ cells, we synthesized Atezo‐TRX4 heterodimer by linking the anti‐PD‐L1 antibody Atezo with the anti‐CD3 antibody TRX4, through a single‐stranded DNA (ssDNA) and its complementary DNA (ssDNA_C). Based on the quantification of Figure 6E, ≈60% of the Atezo heavy chain and 70% of the TRX4 heavy chain were conjugated with ssDNA, respectively (lane 2 and lane 3). Samples were then centrifuged in 100 kD Amicon Ultra Centrifugal Filters to remove extra free ssDNA. By measuring protein concentrations before and after this separation step, we found that 90–95% of the antibodies were recovered. The two antibody‐ssDNA conjugates were then hybridized to give the Ateo‐TRX4 heterodimer, and ≈48% of the antibody monomers were correctly paired to form bispecific antibodies (Figure 6E; Figure S29A, Supporting Information). Although the reaction solution contains the Atezo‐TRX4 heterodimer, the monomer Atezo‐ssDNA, and the monomer TRX4‐ssDNA_C, we reason that the antibody monomers would not affect the following cells experiments, so we didn't purify the bispecific antibody complexes and used the mixture directly in the following experiments.

Subsequently, we co‐cultured the adherent PD‐L+ MDA‐MB‐231 cells with CD3+ T cells, and supplemented the medium with monoclonal antibodies or the Atezo‐TRX4 heterodimer respectively, for 4 h at 37 °C. Unbound T cells were subsequently removed through gentle washing with PBS, because they are suspension cells, whilst MDA‐MB‐231 cells are adherent. The Atezo‐TRX4 heterodimer promoted the binding of CD3+ T cells to MDA‐MB‐231 cells. In the presence of the Atezo‐TRX4 heterodimer, a significantly larger number of T cells adhered to the PD‐L1+ MDA‐MB‐231 cells, compared to the groups that received monoclonal antibodies alone, or the Tras‐TRX4 heterodimer (Figure 6F; Figure S29B and S30, Supporting Information). The Atezo‐TRX4 heterodimer, therefore, recruited the CD3+ T cells to the PD‐L1+ MDA‐MB‐231 cells, so they are bispecific engagers, or we call them DNA‐templated bispecific antibody conjugates. Next, we explored the cytotoxicity of the T cells to MDA‐MB‐231 cells following the engagement of T cells. Following a 48‐h coincubation of MDA‐MB‐231 cells and activated T cells in the presence or absence of various antibody preparations, an LDH cytotoxicity assay was performed to quantify the cytotoxicity induced by T cells. Notably, the Atezo‐TRX4 heterodimer induced a significant T cell cytotoxicity to MDA‐MB‐231 cells, whereas Atezo and TRX4 alone or the Tras‐TRX4 heterodimer did not cause notable T cell cytotoxicity toward the cancer cell (Figure 6G). These results demonstrate that the Atezo‐TRX4 DNA‐bsAbC efficiently recruits T cells to the targeted cancer cells, activates T cells, and ultimately leads to T‐cell‐induced cytotoxicity.

## 3 Conclusion

Our study presents a site‐selective, efficient, and mild Tyr reaction coupling the tyrosinase oxidation and nucleophilic addition reactions. This conjugation reaction proceeds with good chemo‐ and site‐selectivity and functional group tolerance, allowing for site‐selective functionalization of peptides, recombinant proteins, and IgGs. The conjugate linkage is stable in physiological, acidic, and basic solutions. Site‐selective mono‐functionalization of IgGs at Y296 enables the construction of immunoliposomes carrying a lipid chain at the heavy chain for targeted drug delivery. Site‐selective IgG‐DNA conjugates were synthesized, enabling the construction of DNA‐templated antibody dimers. Bispecific antibody dimers constructed via DNA hybridization recruit effector T cells to cancer cells and induce the killing effect against cancer cells, known as antibody‐dependent cellular cytotoxicity (ADCC). The antibody dimers are thus a type of bispecific engagers for effector cells and may have potential for cancer treatments.

## Conflict of Interest

The authors declare no conflict of interest.

## Supporting information



Supporting Information

## Data Availability

The data that support the findings of this study are available in the supplementary material of this article.
