# Supplementary material for: Bioorthogonal Reaction of o‐Quinone with Furan‐2(3H)‐One for Site‐Selective Tyrosine Conjugation and Construction of Bispecific Antibody Complexes
Source: Adv Sci (Weinh). 2025 Apr 25;12(22):2417260. doi: 10.1002/advs.202417260 (PMC12165061; doi:10.1002/advs.202417260)
Supplement: Supplementary file 1 — Supporting Information [file ADVS-12-2417260-s001.pdf]

ADVANCED SCIENCE
Open Access

Supporting Information

for *Adv. Sci.*, DOI 10.1002/advs.202417260

Bioorthogonal Reaction of *o*-Quinone with Furan-2(*3H*)-One for Site-Selective Tyrosine Conjugation and Construction of Bispecific Antibody Complexes

Hongfei Chen, Zhiyi Xu, Yishu Bao, Farshad Shiri, Dingdong Yuan, Yuke Hu, Biquan Li, Bin Zeng, Xiaojing Li, Hao Kong, Zikang Wang, Wilson Chun-Yu Lau, Zhenyang Lin and Jiang Xia**

Supporting Information

Bioorthogonal Reaction of *o*-Quinone with Furan-2(*3H*)-one for Site-Selective Tyrosine Conjugation and Construction of Bispecific Antibody Complexes

Hongfei Chen,[a,†] Zhiyi Xu,[a,†] Yishu Bao,[a,†] Farshad Shiri,[b,†] Dingdong Yuan,[a] Yuke Hu,[a] Biquan Li,[a] Bin Zeng,[a] Xiaojing Li,[a] Hao Kong,[a] Zikang Wang,[c] Wilson Chun-Yu Lau,[c] Zhenyang Lin,[b,*] Jiang Xia[a,*]

[a] Department of Chemistry, The Chinese University of Hong Kong, Shatin, Hong Kong SAR, China.

[b] Department of Chemistry, Hong Kong University of Science and Technology, Shatin, Hong Kong SAR, China.

[c] Department of Applied Biology and Chemical Technology, The Hong Kong Polytechnic University, Hong Kong SAR, China.

*Address correspondence to chzlin@ust.hk and jiangxia@cuhk.edu.hk ORCID 0000-0001-8112-7625

Phone: (852) 3943 6165

Fax: (852) 2603 5057

[†] H. C., Z. X., Y. B., and F. S. contributed equally to this work.

The authors declare no conflict of interest.

Contents

Items	Page No.
**Detailed experimental procedures**	**S4**
**Figure S1.** Structure confirmation of the model reaction.	**S18**
**Figure S2.** Kinetic study of the peptide reaction.	**S20**
**Figure S3.** Stability of the peptide conjugation product.	**S21**
**Figure S4**. Reaction between Fmoc-GGY-OH and **FuA-Phe.**	**S22**
**Figure S5**. Reaction between Fmoc-MGY-OH and **FuA-Phe**.	**S23**
**Figure S6**. Reaction between Fmoc-IGY-OH and **FuA-Phe**.	**S24**
**Figure S7**. Reaction between Fmoc-VGY-OH and **FuA-Phe**.	**S25**
**Figure S8**. Reaction between Fmoc-FGY-OH and **FuA-Phe**.	**S26**
**Figure S9.** Reaction between Ac-YGGFL-$CONH_2$ and **FuA-Phe**.	**S27**
**Figure S10.** Reaction between Ac-VYIHPF-$CONH_2$ and **FuA-Phe**.	**S28**
**Figure S11.** Reaction between Fmoc-FGY-OH and **FuA-PEG-N_3**.	**S29**
**Figure S12.** Reaction between Ac-YGGFL-$CONH_2$ and **FuA-PEG-N_3**.	**S30**
**Figure S13.** Reaction between Ac-VYIHPF-$CONH_2$ and **FuA-PEG-N_3**.	**S31**
**Figure S14.** Reaction pathways leading to two configurational isomers.	**S32**
**Figure S15**. Characterization of GFP-GGY.	**S33**
**Figure S16**. Reaction of GFP-GGY and **FuA-PEG-N_3**.	**S34**
**Figure S17.** Reaction of GFP-GGY and **FuA-TAM**.	**S35**
**Figure S18**. Reaction of GFP-GGY and **FuA-PEG-Alkyne**.	**S36**

**Figure S19.** Reaction of GFP-GGY and **FuA-PEG-biotin**.	**S37**
**Figure S20.** LC-MS/MS characterization of GFP-GGY/**FuA-PEG-N_3** conjugate.	**S38**
**Figure S21.** Reaction of HER2-nanobodies**.**	**S39**
**Figure S22.** The reaction of atezolizumab (a humanized IgG1 monoclonal antibody) with different concentrations of **FuA-PEG-N_3**.	**S40**
**Figure S23.** Sequence comparison of human IgG1 heavy chains.	**S41**
**Figure S24.** Reactions of Human IgG1s.	**S43**
**Figure S25.** LC-MS/MS characterization of reaction site of Atezo/**FuA-PEG-N_3** conjugate.	**S44**
**Figure S26.** Confocal images of SKOV3 cells and MDA-MB 231 cells incubated with Tras-TAMRA.	**S45**
**Figure S27.** Confocal images of liposome-cell fusion.	**S46**
**Figure S28**. Representative EM images of the IgG dimers.	**S47**
**Figure S29**. Bispecific antibody and Jurkat cells recruitment.	**S48**
**Figure S30.** Confocal images show that the Atezo-TRX4 heterodimer engages T cells to MDA-MB-231 cells.	**S49**
**Figure S31.** NMR spectrums of **FuA**.	**S50**
**Figure S32.** NMR spectrums of **FuA-PEG-N_3**.	**S51**
**Figure S33.** NMR spectrums of **FuA-Phe**.	**S52**
**Figure S34.** NMR spectrums of **FuA-PEG-alkyne**.	**S53**
**Figure S35.** NMR spectrums of **FuA-PEG-Biotin**.	**S54**
**References**	**S55**

Detailed experimental procedures

Materials and Instruments. Unless otherwise noted, all reagents were used without further purification. Fmoc-protected amino acids and coupling reagents were obtained from GL Biochem Ltd. (Shanghai, China). Rink amide resins were obtained from Biotage (Uppsala, Sweden). 5(6)-TMARA was purchased from Beijing Okeanos Technology Co., Ltd. (Beijing, China). 2-azidoacetic acid, DBCO-TMARA (Catalogue number 760773), and tyrosinase (from mushroom, product number T3824-25KU) were purchased from Sigma-Aldrich Co. (USA). PNGase F was obtained from New England Biolabs (NEB). Human IgG (Catalogue number ab91102) was purchased from Abcam. In-Gel Tryptic Digestion Kit was purchased from Thermo Fisher Scientific Inc. (USA). Single strain DNA used were purchased from BGI company (Shenzhen, China). Peptide characterization and purification were performed in RP-HPLC (Shimadzu, DGU-20A5, Japan). Peptide analysis was performed in an AutoFlex Speed LRF MALDI-TOF mass spectrometer (Bruker Daltonics, Germany). The gel images were captured by an ENDURO™ GDS Gel Documentation System (USA) or a Bio-Rad ChemiDoc Image System (USA).

Peptide synthesis. All Peptides were synthesized based on manual Fmoc-SPPS chemistry. Briefly, Rink Amide-ChemMatrix® resins (Biotage, Sweden) with a loading capacity of 0.5 mmol/g were first swelled by DCM/DMF (50% v/v). For each coupling procedure, a five-fold excess of protected amino acid, HBTU, HOBt, and DIEA (with a ratio of 1: 1: 1: 2) in DMF was added to the resin for 35 min with shaking at RT. The deprotection reaction of the Fmoc group was performed in 20% piperidine in DMF (v/v) after the resins were washed with DMF 5 times. For capping the N-terminus amine, the resin was suspended in a DMF solution containing acetic anhydride (10 equivalents based on resin

substitution) and DIEA (10 equivalents based on resin substitution) and shaken at RT for 30 min. Normally, peptides were cleaved from the resin, and the sidechains were deprotected by treatment with TFA/H_2O/TIPS (95/2.5/2.5) for 2 h at RT. Then the resin was filtered and rinsed twice with TFA. The crude peptide was obtained by precipitation by adding cold diethyl ether.

Peptide purification and characterization. Crude peptides were dissolved in 50% ACN: 50% H_2O containing 0.1% TFA. After being filtered through a 0.2 μm filter, the peptide solution was injected into RP-HPLC (Shimadzu, DGU-20A5, Japan) equipped with a C18 column (Vydac 218TP C18 LC Column 5um, 250x4.6mm ID). 0.1% TFA in H_2O (v/v) and 0.1% TFA in ACN (v/v) were used as the mobile phases A and B, respectively. For analytical HPLC analyses, the total flow rate was set to be 1 mL/min, and the B concentration rose from 5 % to 95 % over 13 min following a linear gradient. For the purification of peptides on a larger scale by semi-prep HPLC columns (Vydac 218TP C18 LC Semi-Prep Column 10um, 250x10mm ID), the total flow rate was set to be 3 mL/min (gradient: 0-5 minutes 5% B, 5-30 minutes 5-65% B, 30-33 minutes 65-95% B, 33-36 minutes 95% B). The peptide peaks were collected, lyophilized, and confirmed by MALDI-TOF mass spectrometry analysis (Bruker Daltonics, Germany).

The model reaction of 1a and 2a. Substrates **1a** (0.1 mmol), **4-MBQ** (0.5 mmol) were dissolved in PB buffer (0.2M, pH 6.5). The reaction system was stirred for 30 min at room temperature. The reaction solution was extracted by EtOAc (2 mL) three times. Then, the product was purified by silica column chromatography (EtOAc/hexane=3/1).

^{1}H NMR (500 MHz, Chloroform-*d*) δ 7.86 (dd, *J* = 5.7, 1.5 Hz, 1H), 6.91 (d, *J* = 1.4 Hz, 1H), 6.68 (s,

1H), 6.07 (dd, J = 5.7, 1.5 Hz, 1H), 2.35 (s, 3H), 1.79 (s, 3H).

^{13}C NMR (125 MHz, Chloroform-*d*) δ 159.65, 143.24, 141.23, 129.82, 127.71, 119.69, 119.40, 113.50, 90.00, 26.41, 20.66.

HRMS (ESI) calcd. for $C_{12}H_{12}O_4Na^+$ 243.0628 $[M+Na]^+$; found 243.0623.

Peptide reactions. The starting peptide Fmoc-GGY-OH (10 mM, 5 μL), **FuA-Phe** (100 mM, 5 μL), and 25 μL DI water were incubated with tyrosinase (1.68 μM) at room temperature in PB Buffer (0.2 M, pH 6.5, 10 μL) for 30 min. For the kinetic study, the reaction rate constants of conjugation of Fmoc-GGY-OH oxidized form and **FuA-Phe** were calculated based on absorbance at 390 nm (the maximum absorbance of quinone). Percent conversion (x) was calculated by the standard addition method. Pseudo-first order rate constant k_1 was measured by plotting ln[1/(1-x)] versus time and analysis by linear regression. For other peptide reactions, the reaction yield was determined by the integrated peak area of the product in HPLC chromatograms (benzophenone was used as internal standard compound), and the new peak was subjected to MALDI-TOF MS analysis to confirm it as the conjugation product. The reaction profile was analyzed by HPLC (monitored at 254 nm or 215 nm) and the yield was determined by HPLC peak area (254 nm or 215 nm) following the calculation below:

$$\text{yield (\%)} = \frac{\text{A (modified peptide)}}{\sum \text{A (peptide components)}} * 100$$

Stability analysis of peptide conjugation product.

Plasmid construction. *E. coli* TOP10 strain was used for cloning and plasmid propagation and grown in selective Luria-Bertani medium or Luria Bertani plates with 1.5 wt% agar. Antibiotics were added for selection at the following concentrations: 100 μg/mL ampicillin or 50 μg/mL kanamycin. The DNA

sequences of the plasmid constructs containing PCR fragments were confirmed by sequencing. The primers were ordered from BGI. Plasmids were constructed using standard restriction enzyme cloning techniques.

Expression and purification of proteins. BL21 (DE3) cells were grown at 37 ºC until cultures reached an OD600 value between 0.6-0.8, upon which protein expression was induced with a final concentration of 0.1 g/L IPTG. Cells were then shaken overnight at 37 ºC and pelleted. Then cell pellets were resuspended in 15 mL of an equilibration buffer (20 mM sodium phosphate, 2 M NaCl, 20 mM imidazole at pH = 7.4) and then lysed via sonication for 30 min at 60% amplitude. The cell lysate was centrifuged at 14,000 rpm for 30 min; then, the supernatant was decanted before loading onto a HisTrap Crude column. After binding, the bound protein was washed with four portions of two resin bed volumes of wash buffer (20 mM sodium phosphate, 300 mM NaCl, 25 mM imidazole at pH 7.4) and subsequently eluted with four resin bed volumes of elution buffer (20 mM sodium phosphate, 300 mM NaCl, 250 mM imidazole at pH = 7.4). The purified protein was then spun-concentrated into 20 mM phosphate buffer at pH 7.2 using 10 kDa MWCO or 30 kDa MWCO filters. Purified protein samples were flash-frozen and stored at -80 ºC until use.

Reactions of the recombinant proteins and product analysis. Protein (10 μM) was incubated with tyrosinase (100 nM) and small molecules (1000 μM) in PB buffer (0.2 M, pH 7.4) at room temperature for 60 min. 12% acrylamide gels were prepared according to BIO-RAD bulletin 6201 protocol. 12 μL 0.5 mg/mL protein solution was diluted with 3 μL 5 × sample buffer, including 5% 2-mercaptoethanol, and heated to 95 °C for 10 minutes. After loading the samples, the gel was run using a BIO-RAD Mini-

PROTEAN Tetra Vertical Electrophoresis Cell at 150 volts until completion. Fluorescently labeled proteins were analyzed prior to staining using a BioRad ChemiDoc™ system. Subsequently, the gel was stained using a staining solution containing 1 g/L Coomassie Brilliant Blue R-250 in 5:4:1 (v/v/v) methanol: water: acetic acid for 30 min. The gel was subsequently destained using 5:4:1 (v/v/v) methanol: water: acetic acid for 60 min, after which it was further destained overnight using deionized water.

SDS-PAGE analysis. Add 2 μg of the monoclonal antibody into SDS Sample Buffer (5 X) with the supplement of 18% β-mercaptoethanol and boil samples for 10 min before loading. For DNA-bsAbs, heat the samples at 55°C for 10 min before loading to avoid the denature of dsDNA. Run the samples on a pre-cast SDS polyacrylamide gel at 90V for 15 min and 160 V for 40 to 60 minutes until the dye reaches the bottom of the gel. Remove the gel and incubate it in the Coomassie blue stain solution on the shaker at 60 rpm for 10 min. Replace the Coomassie blue stain solution with ddH_2O and incubate the gel on the shaker until the protein lane is clear. Use Bio-Rad ChemiDoc Image System (USA) to detect the signal.

Western Blotting experiments. Add 5 μg of the modified protein into SDS Sample Buffer (5 X) and boil samples for 10 min before loading. Run the samples on a pre-cast SDS polyacrylamide gel at 170V (constant voltage) for 40 to 60 minutes until the dye reaches the bottom of the gel. Remove the gel and soak it in 1L of protein transfer buffer for 15 minutes. Cut the nitrocellulose membrane (ISC BioExpress, F-3139-3) to a similar size to the transfer area of the gel. Assemble the electroblotting cassette and place the electrodes in the blotting unit, then transfer in the transfer buffer at 100 V for 1

hour at a constant current (not to exceed 0.4 A). Following the transfer, remove the membrane from the blotting cassette and mark the orientation of the gel with a pencil. Rinse briefly with PBS. Wash the membrane with TBST (10 mM Tris-HCl, pH 8.0, 150 mM NaCl, 0.05% Tween 20) once for 5 min at room temperature. Block non-specific binding on the membrane with freshly prepared 5% nonfat dried milk for 1 hour on a shaking platform at room temperature. Incubate the membrane with primary biotin antibody (BK-1/39, SC-53179 from Santa Cruz) diluted in TBST and 5% nonfat dried milk at 4° C overnight. Wash three times for 5 min each with TBST. Incubate with Anti-mouse-IgG, HRP-conjugated secondary antibody (7076S from Cell Signaling Technology), in TBST-5% nonfat dried milk for 1 hour at room temperature. Wash three times again for 5 minutes each with TBST. For detection, use Western Blot Luminol Reagent and prepare according to instructions. Lay the membrane on a plastic surface with the protein side up. Add the mixed detection solution to the membrane. Incubate for 1 minute. Remove the excess solution and cover the membrane with transparent plastic. Use Bio-Rad ChemiDoc Image System (USA) to detect the signal.

Antibody deglycosylation. Trastuzumab (200 μg, 5 mg/mL in PBS pH 7.4) was incubated with PNGase F (2.0 μL, 250 units) at 37 °C. After overnight incubation, the antibody was dialyzed (3 times to PBS pH 5.5) and concentrated to 2.0 mg/mL.

Procedure for antibody conjugation. Deglycosylated trastuzumab (20 μL, 2.0 mg/mL, 40 μg in PBS pH 5.5, final concentration 5 μM) was diluted with 20 μL PB buffer pH 7.4 and incubated with **FuA-PEG-N_3** (5 μL, 5 mM in DMSO, final concentration 500 μM) and mushroom tyrosinase (5.0 μL, 1.7 mg/mL in phosphate buffer pH 6.0, final concentration 2 μM) and incubated for 8 h at 4 °C. After

completion, the product was concentrated, and small molecules were removed by 30 kDa MWCO filters. After that, DBCO-PEG$_4$-TAMRA (5 µL, 2 mM in DMSO) was added and incubated at room temperature for 1.5 h.

Cell imaging experiment. 1×10^5 SKOV3 or MDA-MB-231 cells were seeded in 35-mm glass-bottom tissue culture dishes. When reaching 80% confluency, cells were incubated with Trastuzumab-(FuA)-TAMRA (30 µg/mL) for 45 minutes and then washed with PBS three times. After that, cells were incubated with DAPI (10 µg/mL) at 37 °C for 10 min. After washing with PBS, cells were imaged immediately under a Leica confocal microscope with corresponding filters.

Detailed synthetic procedures of chemical molecules:

4-(5-oxo-4,5-dihydrofuran-2-yl) butanoic acid (FuA)

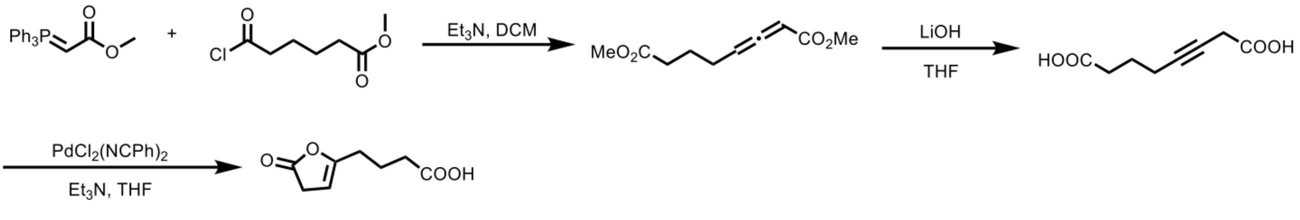

FuA

FuA was synthesized according to a previous report. [1] All starting materials were commercially available and purchased from J&K Scientific Ltd. (Beijing, China). The final product was purified by column chromatography (MeOH/DCM=1/50) (Figure S25 below).

^{1}H NMR (500 MHz, Chloroform-*d*) δ 5.20 (t, *J* = 2.1 Hz, 1H), 3.21 (q, *J* = 2.3 Hz, 2H), 2.48 – 2.39 (m, 4H), 1.94 (p, *J* = 7.4 Hz, 2H).

^{13}C NMR (125 MHz, Chloroform-*d*) δ 178.56, 176.72, 155.87, 99.28, 33.94, 32.79, 27.42, 20.78.

HRMS (ESI) calcd. for $C_8H_{10}O_4Na^+$ 193.0471 $[M+Na]^+$; found 193.0471.

N-(2-(2-(2-(2-azidoethoxy)ethoxy)ethoxy)ethyl)-4-(5-oxo-4,5-dihydrofuran-2-yl)butanamide ($FuA-PEG-N_3$)

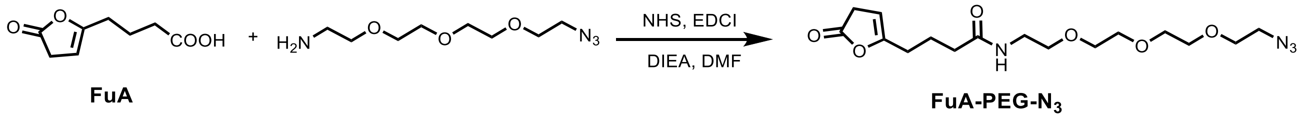

In a round bottle flask, **FuA** (0.05 mmol), NH_2-PEG_3-N_3 (1.2 eq), EDCI (2.0 eq), NHS (2.0 eq), and DIEA (3.0 eq) were dissolved in DMF (5 mL) on an ice bath for one hour. After that, the reaction solution was stirred at room temperature for 12 hours. Then, 30 mL water was added to the flask, and ethyl acetate (10 mL ×3) was used to extract the product three times. The organic layer was washed with NaCl-saturated solution (10 mL) twice and dried with anhydrous Na_2SO_4. Then, the organic layer was concentrated and purified by column chromatography (EA) to get a solid of 5 mg (27% yield) (Figure S26 below).

^{1}H NMR (500 MHz, Chloroform-*d*) δ 5.21 – 5.16 (m, 1H), 3.69 (d, *J* = 4.0 Hz, 8H), 3.65 (dt, *J* = 6.0, 1.7 Hz, 2H), 3.57 (dd, *J* = 5.6, 4.4 Hz, 2H), 3.49 – 3.45 (m, 2H), 3.45 – 3.40 (m, 2H), 3.20 (q, *J* = 2.3 Hz, 2H), 2.40 – 2.35 (m, 2H), 2.27 (d, *J* = 7.4 Hz, 2H), 1.93 (t, *J* = 7.4 Hz, 2H).

^{13}C NMR (125 MHz, Chloroform-*d*) δ 176.85, 172.25, 156.36, 99.02, 70.67, 70.61, 70.52, 70.22, 70.01, 69.90, 50.69, 39.21, 35.30, 33.96, 27.57, 21.80.

HRMS (ESI) calcd. for $C_{16}H_{26}N_4O_6Na^+$ 393.1742 $[M+Na]^+$; found 393.1744.

4-(5-oxo-4,5-dihydrofuran-2-yl)-N-(p-tolyl) butanamide (FuA-Phe)

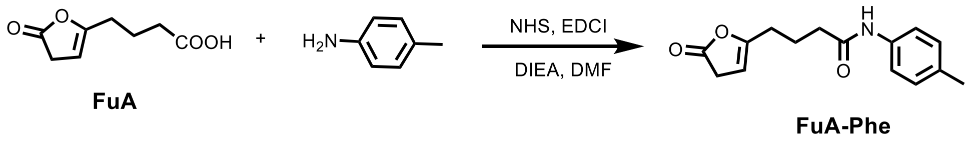

The compound was synthesized according to the above procedures with 10 mg withe solid obtained

(38% yield) in 0.1mmol scale starting materials. (Figure S27 below)

^{1}H NMR (500 MHz, Chloroform-*d*) δ 7.41 (d, *J* = 8.4 Hz, 2H), 7.15 (d, *J* = 8.2 Hz, 2H), 5.22 (s, 1H), 3.21 (t, *J* = 2.3 Hz, 2H), 2.47 – 2.42 (m, 4H), 2.34 (s, 3H), 2.07 – 2.00 (m, 2H).

^{13}C NMR (125 MHz, Chloroform-*d*) δ 176.75, 170.11, 156.25, 135.15, 134.06, 129.54, 119.86, 99.24, 36.32, 33.96, 27.51, 21.81, 20.87.

HRMS (ESI) calcd. for $C_{15}H_{17}NO_3Na^+$ 282.1101 $[M+Na]^+$; found 282.1098.

FuA-PEG-TAM

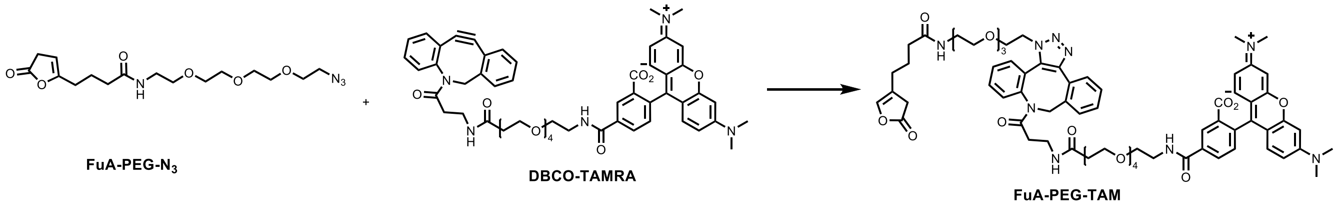

The compound was synthesized by combining **FuA-PEG-N$_3$** and DBCO-TAMRA in DMF with 1:1 ratio for 12 h. The product was purified by RP HPLC.

HRMS (ESI) calcd. for $C_{70}H_{83}N_9O_{16}Na^+$ 1328.2850 $[M+Na]^+$; found 1328.5841.

4-(5-oxo-4,5-dihydrofuran-2-yl)-N-(2-(2-(prop-2-yn-1-yloxy)ethoxy)ethyl)butanamide (FuA-PEG-Alkyne)

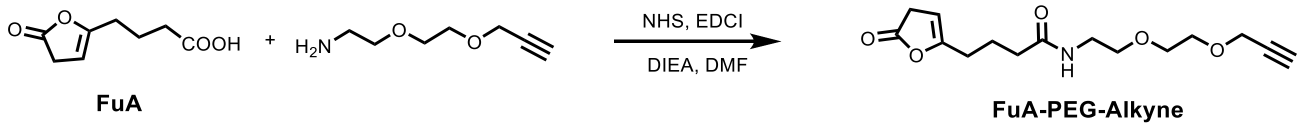

The compound was synthesized according to the above procedures with 22 mg yellow solid obtained (48% yield) in 0.1mmol scale starting materials. (Figures S28 below)

^{1}H NMR (500 MHz, Chloroform-*d*) δ 5.14 – 5.03 (m, 1H), 4.23 (d, *J* = 2.4 Hz, 2H), 3.74 – 3.66 (m, 4H), 3.59 (t, *J* = 5.1 Hz, 2H), 3.52 – 3.45 (m, 2H), 2.49 (t, *J* = 2.4 Hz, 1H), 2.28 (td, *J* = 7.1, 5.3 Hz,

2H), 1.98 – 1.72 (m, 4H).

^{13}C NMR (125 MHz, Chloroform-*d*) δ 173.04, 172.14, 156.13, 121.72, 83.13, 74.84, 70.05, 69.81, 69.10, 58.47, 39.15, 35.67, 32.49, 21.12.

HRMS (ESI) calcd. for $C_{15}H_{21}NO_5Na^+$ 318.1312 [M+Na]$^+$; found 318.1304.

N-(13-oxo-16-(5-oxo-4,5-dihydrofuran-2-yl)-3,6,9-trioxa-12-azahexadecyl)-5-((3aS,4R,6aR)-2-oxohexahydro-1H-thieno[3,4-d]imidazol-4-yl)pentanamide (FuA-PEG-Biotin)

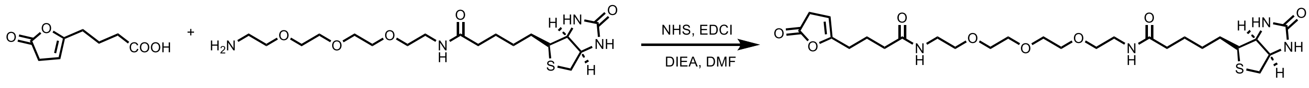

The compound was synthesized according to the above procedures with 6 mg yellow solid obtained (17.5% yield) in 0.05mmol scale starting materials. (Figures S29 below)

^{1}H NMR (500 MHz, Methanol-*d*$_4$) δ 5.16 (ddt, *J* = 6.9, 4.8, 1.8 Hz, 1H), 4.49 (dd, *J* = 7.9, 4.9 Hz, 1H), 4.31 (dd, *J* = 7.9, 4.4 Hz, 1H), 3.65 – 3.60 (m, 8H), 3.54 (t, *J* = 5.5 Hz, 4H), 3.36 (t, *J* = 5.5 Hz, 4H), 3.30 (p, *J* = 1.6 Hz, 2H), 3.21 (ddd, *J* = 8.9, 5.8, 4.4 Hz, 1H), 2.92 (dd, *J* = 12.8, 5.0 Hz, 1H), 2.70 (d, *J* = 12.8 Hz, 1H), 2.28 – 2.20 (m, 4H), 1.89 – 1.80 (m, 1H), 1.77 – 1.69 (m, 3H), 1.63 (dddd, *J* = 23.9, 14.9, 7.7, 2.9 Hz, 4H), 1.44 (p, *J* = 7.4 Hz, 2H).

^{13}C NMR (125 MHz, Chloroform-*d*) δ 173.61, 173.27, 172.69, 164.36, 156.60, 156.58, 121.51, 83.31, 70.34, 70.32, 70.07, 69.99, 69.87, 61.90, 60.36, 55.62, 40.50, 39.20, 39.14, 35.83, 35.51, 32.46, 28.18, 28.04, 25.59, 21.23.

HRMS (ESI) calcd. for $C_{26}H_{42}N_4O_8SNa^+$ 593.2616 [M+Na]$^+$; found 593.2616.

Proteins used in this study. The primers of GFP-GGY were designed according to previous work.[3] And the plasmids of Her2-nanobody-GGY and Her2-nanobody-GGF were directly purchased from

BGI Inc. (Shenzhen, China).

a) GFP-GGY

Forward：5’- AATTCGGAGGAGGAGGTTCTGGCGGCTATTAAC-3’

Reverse：5'- TCGAGTTAATAGCCGCCAGAACCTCCTCCTCCG -3'

protein sequences:

MHHHHHHMASMTGGQQMGRGSMVSKGEELFTGVVPILVELDGDVNGHKFSVSGEGEGDA

TYGKLTLKFICTTGKLPVPWPTLVTTLTYGVQCFSRYPDHMKQHDFFKSAMPEGYVQERTIF

FKDDGNYKTRAEVKFEGDTLVNRIELKGIDFKEDGNILGHKLEYNYNSHNVYIMADKQKNG

IKVNFKIRHNIEDGSVQLADHYQQNTPIGDGPVLLPDNHYLSTQSALSKDPNEKRDHMVLLE

FVTAAGITLGMDELYKELRRQASGGGGSGGY*

b) HER2-Nanobody-GGY

protein sequences:

HHHHHHSSGLVPRGSHMQVQLQESGGGSVQAGGSLKLTCAASGYIFNSCGMGWYRQSPGR

ERELVSRISGDGDTWHKESVKGRFTISQDNVKKTLYLQMNSLKPEDTAVYFCAVCYNLETY

WGQGTQVTVSSGGGGSGGY*

c) HER2-Nanobody-GGF

protein sequences:

HHHHHHSSGLVPRGSHMQVQLQESGGGSVQAGGSLKLTCAASGYIFNSCGMGWYRQSPGR

ERELVSRISGDGDTWHKESVKGRFTISQDNVKKTLYLQMNSLKPEDTAVYFCAVCYNLETY

WGQGTQVTVSSGGGGSGGF*

Sequences of DNA strands employed in antibody multimer formation.

1) ssDNA:

GGGCTCATGCGAGGCTTACGAAC (5'to 3', 5' end was modified by a C6 amine group)

2) ssDNA-C:

GTTCGTAAGCCTCGCATGAGCCC (5'to 3', 5' end was modified by a C6 amine group)

DBCO-ssDNA synthesis. ssDNA (1 *eq.*) was incubated with DBCO-PEG$_4$-NHS (20 *eq.*) in PBS (pH 7.4) for 12 h. The product was purified by HPLC. The DBCO group can be detected at wavelength 310 nm. The purified product was lyophilized and stored at -80℃.

Antibody-ssDNA conjugation. Atezo-N$_3$ (5μM) was incubated with DBCO-ssDNA (10 μM) at 37℃ for 12h. The product was purified by an anion exchange column with FPLC. The purified product was stored at -80°C.

Antibody dimer formation. Purified Atezo-ssDNA and Atezo-ssDNA-C were hybridized in PBS (1:1 molar ratio, 1 μM) and incubated at 37ºC for 30 minutes. The product was analyzed using negative stained EM.

T cells incubation. T cells were amplified from eripheral blood mononuclear cells (PBMCs) of healthy donors in OptiVitro T Cell Medium SF (TE000-N052, ExCell) with 10 ng/mL IL-2 (CT-128A, Wissen) and ImmunoCult™ Human CD3/CD28 T Cell Activator (10971, Stemcell) for one week and cryopreserved in 90% FBS with 10% DMSO.

T cells and cancer cells coincubation. [2] 1×10^5 MDA-MB-231 or SKBR3 cells were seeded in 35-mm glass-bottom tissue culture dishes and cultured overnight. 4×10^5 T cells or THP-1 cells or Jurkat cells were pre-cultured with 50nM antibodies for 2 hours. Medium in the culture dishes were removed, and HP-1 cells or Jurkat cells with or without antibodies were added to the culture dishes. After 4 hours, cells were gently washed with PBS three times and imaged under a confocal microscope (Stellaris 8, Leica).

In-Vitro Cytotoxicity Assay. 2×10^4 cells/well of MDA-MB-231 cells were seeded in the 96-well plate in the complete DMEM medium and incubated overnight at 37°C in a humidified 5% CO_2 atmosphere. T cells were thawed 2 days before the experiment and incubated in OptiVitro T Cell Medium SF (TE000-N052, ExCell) with 10 ng/mL IL-2. Then, T cells were preincubated with either 100 nM Atezo-TRX4 DNA-bsAbs, 100 nM Atezolizumab antibody, 100 nM TRX4 antibody, or 100 nM Tras-TRX4 DNA-bsAbs for 1 hour separately. Then, the T cells were mixed with MDA-MB-231 cells at the density of 4×10^4 cells/well. After 48 hours, the cytotoxicity was measured with the extracellular LDH detection using LDH Cytotoxicity Assay Kit (C0016 from Beyotime) according to the manufacturer's instructions. Maximal cell LDH release value was achieved by the MDA-MB-231 cells blank group with the addition of LDH release reagent. Spontaneous LDH release value was assessed using MDA-MB-231 cells without T cells and antibodies. The percentage of cytotoxicity towards target cells was calculated based on the following formula:

$$\text{Cytotoxicity (\%)} = \frac{\text{Experiment Value} - \text{Spontaneous LDH release value}}{\text{Maximal cell LDH release value} - \text{Spontaneous LDH release value}} \times 100$$

Computational Details

All the structures discussed in this paper were optimized using Gaussian 16. [3] The optimization was done at the level of theory of the wB97XD functional from Head-Gordon and coworkers. [4] For all of the calculations, solvent effects were considered using the SMD solvation model of Truhlar and workers [5] with water as the solvent. The 6-31G(d) basis set was employed. [6] Frequency calculations were carried out at the same level of theory as those for structural optimization. Transition structures were located using the Berny algorithm. [7] Intrinsic reaction coordinate (IRC) calculations were used to confirm the connectivity between transition structures and minima. [8] To further refine the energies obtained from the SMD/wB97XD/6-31G(d) calculations, we carried out single-point energy calculations using the wB97XD functional method for all of the structures with a larger basis set def2-TZVP. [9] A tight convergence criterion and ultrafine integral grid were also employed to increase the accuracy of the calculations. The relative Gibbs free energy for each species in the solution was calculated using the equation,

$$G = E[\text{def2-TZVP}] + G[\text{6-31G(d)}] - E[\text{6-31G(d)}] + \Delta G^{1atm\rightarrow 1M} \quad (1)$$

where $\Delta G^{1atm\rightarrow 1M}$ = 1.89 kcal/mol is the free-energy change for compression of 1 mol of an ideal gas from 1 atm to the 1 M solution phase standard state. [10]

Coordinates-ESI.xyz file

Please refer to the xyz file for the total potential energy (E), enthalpy (H), and Gibbs free energy (G) of all structures optimized at the SMD/wB97XD/6-31G(d) level of theory. Additionally, the total potential energies calculated by SMD/wB97XD/def2-TZVP//SMD/wB97XD/6-31G(d) in water are also included.

A)

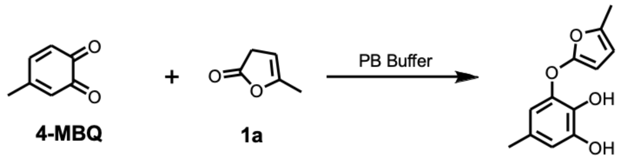
PB Buffer
4-MBQ
1a
3aa

B)

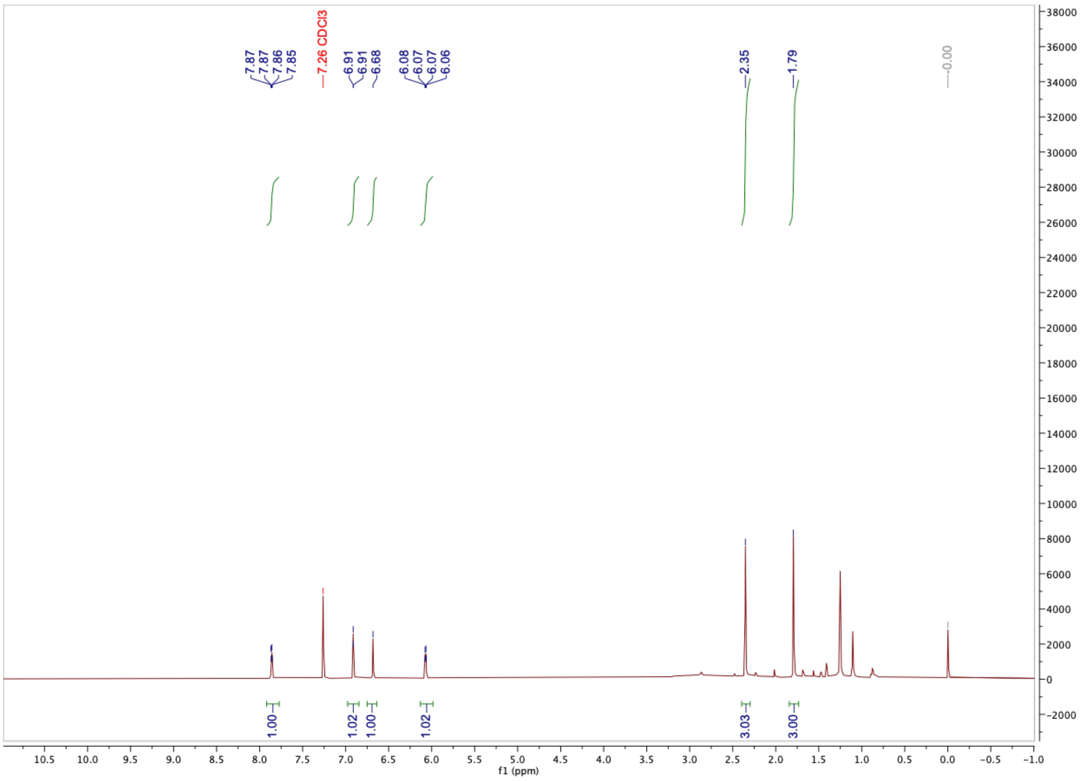
7.87
7.87
7.86
7.85
7.26 CDCl3
6.91
6.91
6.68
6.08
6.07
6.07
6.06
2.35
1.79
0.00
1.00
1.02
1.00
1.02
3.03
3.00
f1 (ppm)

C)

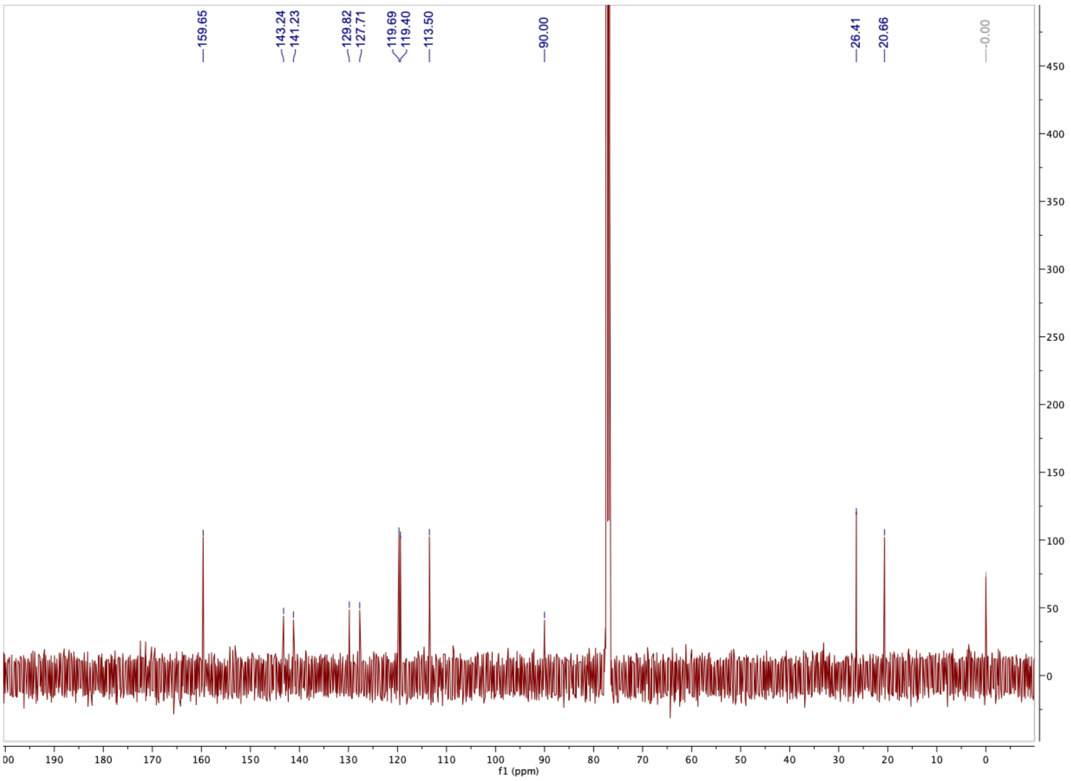
159.65
143.24
141.23
129.82
127.71
119.69
119.40
113.50
90.00
26.41
20.66
0.00
f1 (ppm)

D)

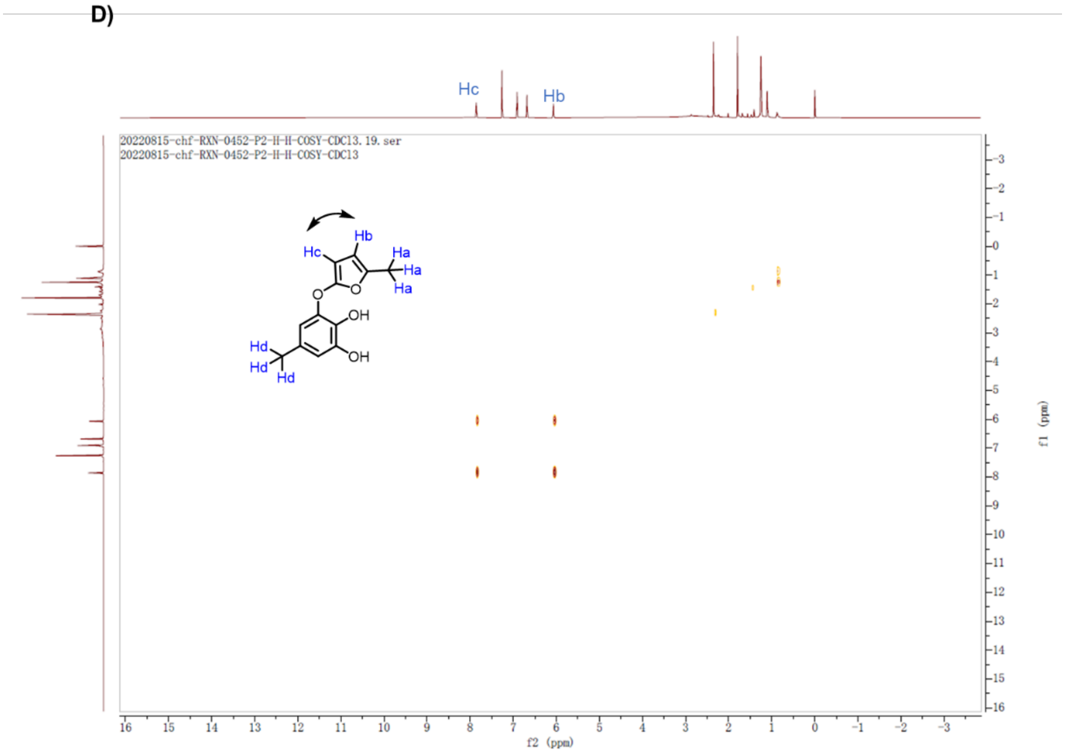

E)

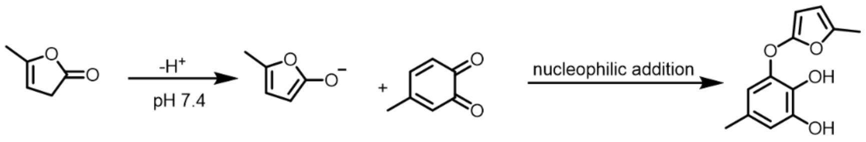

Figure S1. Structure confirmation of the model reaction. (**A**) Reaction scheme. (**B**) H[1]-NMR spectrum of product **3aa**. (**C**) C[13]-NMR spectrum of product **3aa**. (**D**) H[1]-H[1] COSY spectrum of product **3aa**. (**E**) Proposed mechanism of the model reaction.

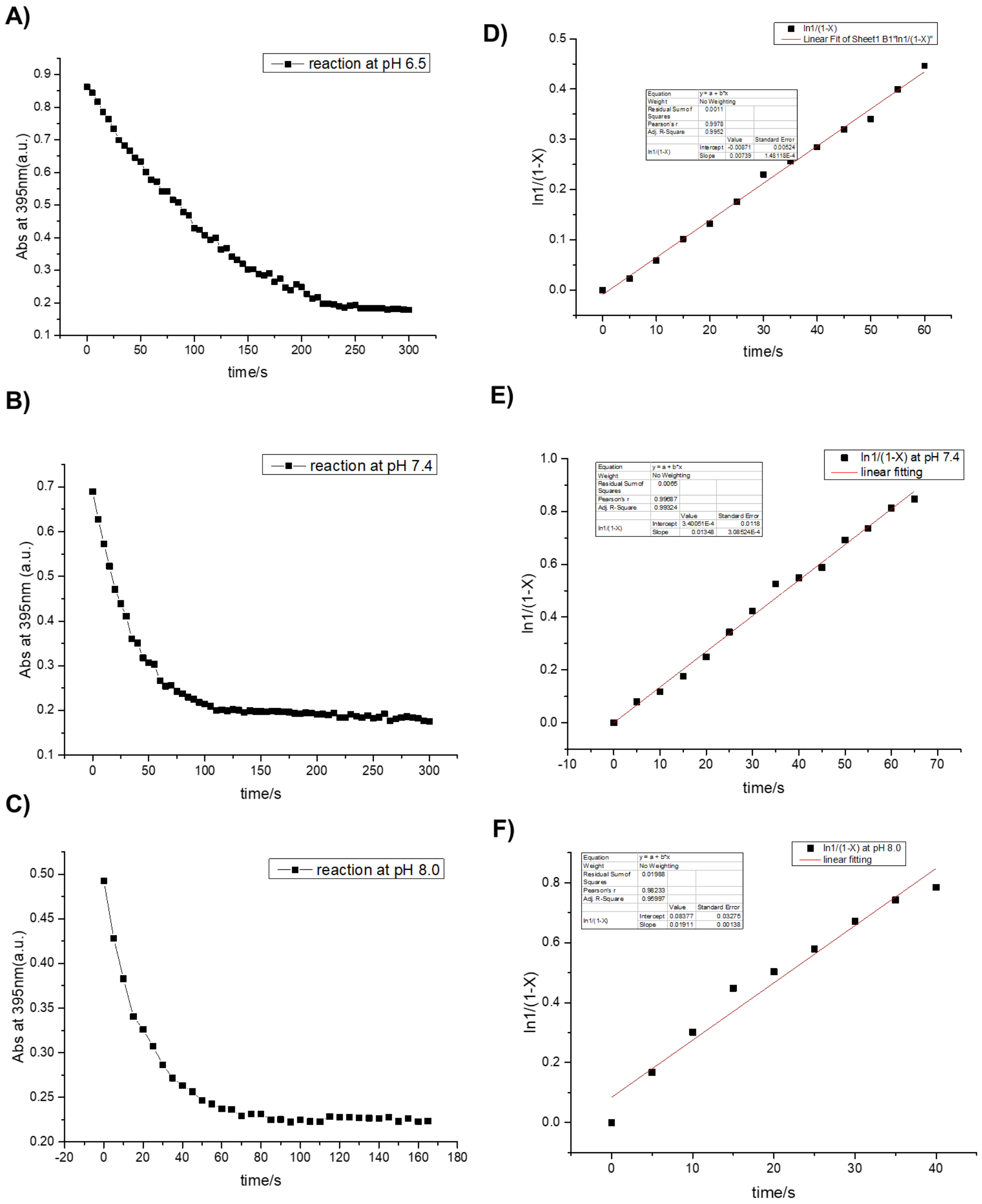

Figure S2. Kinetic study of the peptide reaction. The reaction system monitored at 395 nm and at pH 6.5 (**A**), pH 7.4 (**B**), and pH 8.0 (**C**). Pseudo-first order rate constant *k* of the model reaction at pH 6.5 (**D**), pH 7.4 (**E**), and pH 8.0 (**F**).

A)

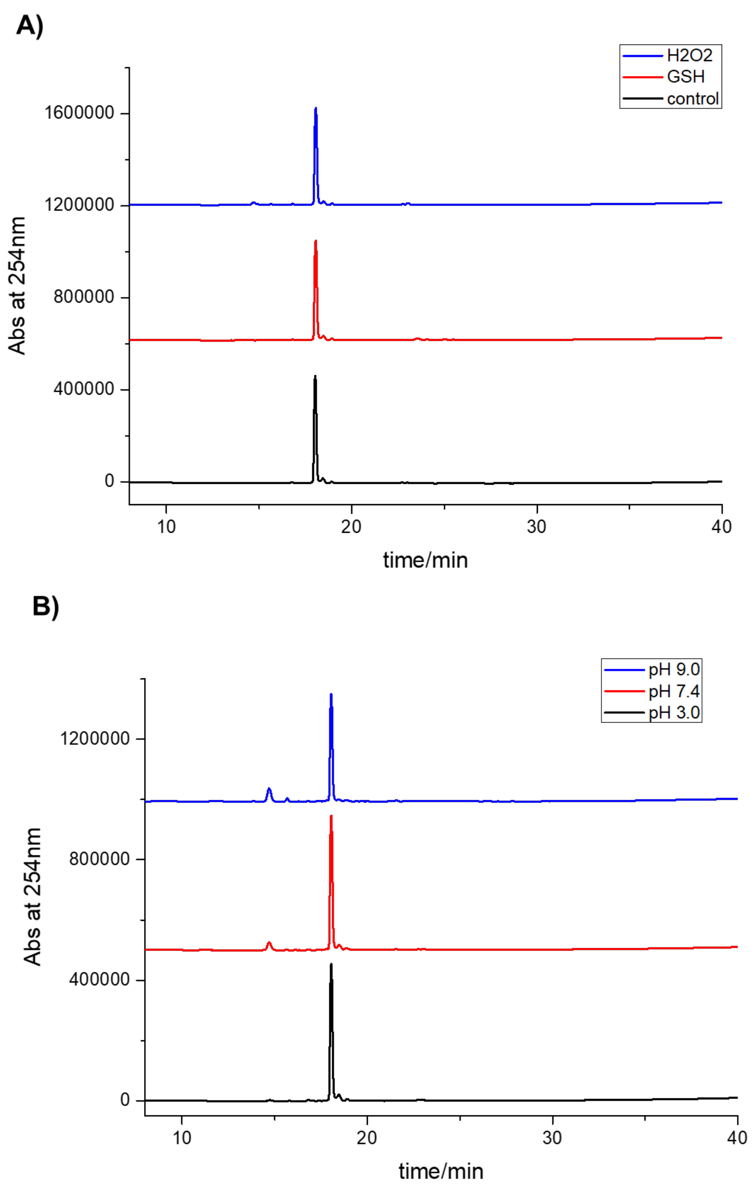

Figure S3. Stability of the peptide conjugation product. (**A**) HPLC spectrum of peptide product incubated with different additives. H_2O_2 (2.5 mM) or GSH (2.5 mM) was incubated with peptide product (0.25 mM) at room temperature for 12 h. (**B**) HPLC spectrum of peptide product (0.25 mM) incubated PB buffer at 37℃ at different pH values for 12 h.

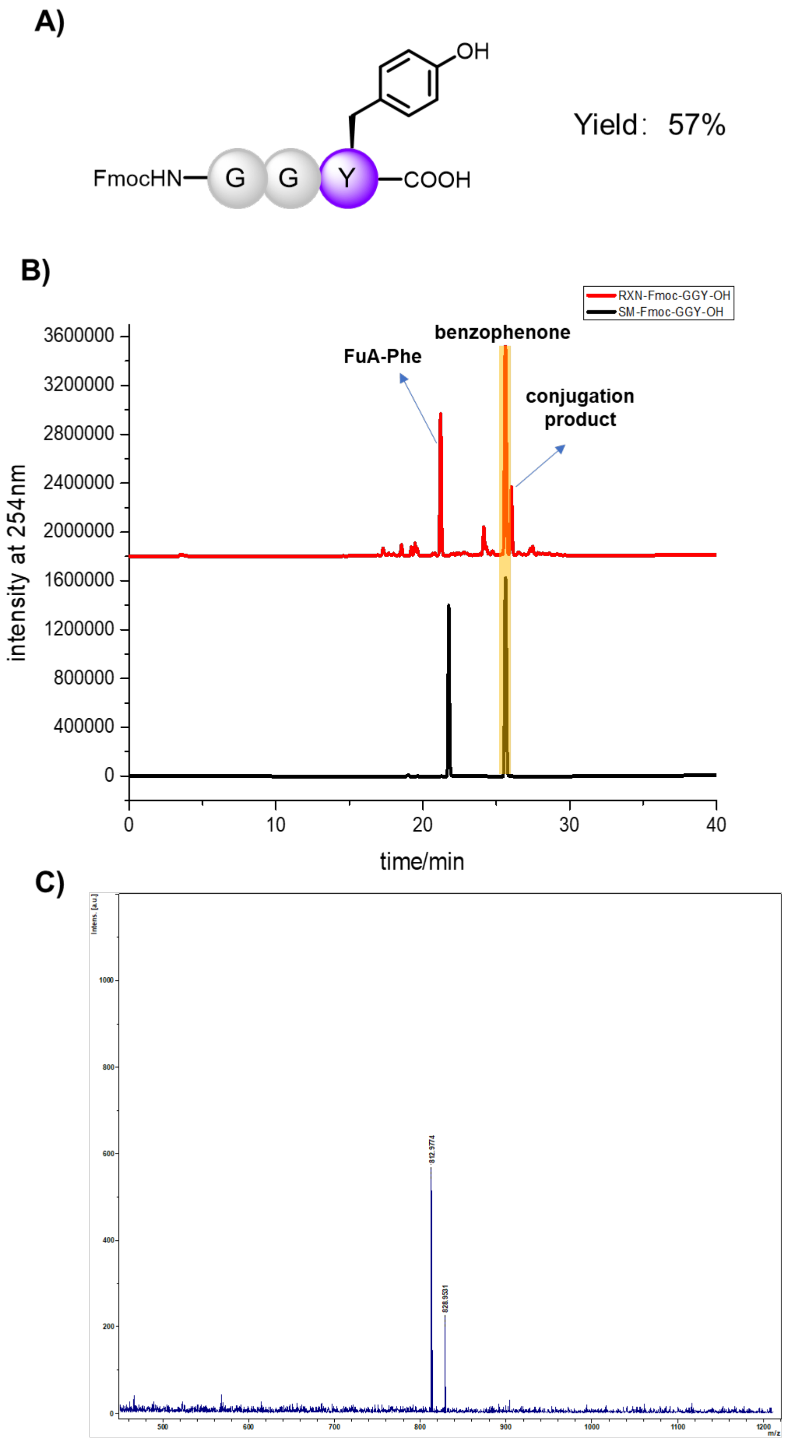

Figure S4. Reaction between Fmoc-GGY-OH and **FuA-Phe**. (**A**) Structure and reaction yield of Fmoc-GGY-OH. (**B**) HPLC spectrum of reaction of Fmoc-GGY-OH. SM: standard material benzophenone. (**C**) MALDI-TOF MS of new peaks calcd for $C_{43}H_{42}N_4NaO_{11}^+$ $[M+Na]^+$ 813.2472, found 812.9774; calcd for $C_{43}H_{42}N_4KO_{11}^+$ $[M+K]^+$ 829.2482, found 828.9531.

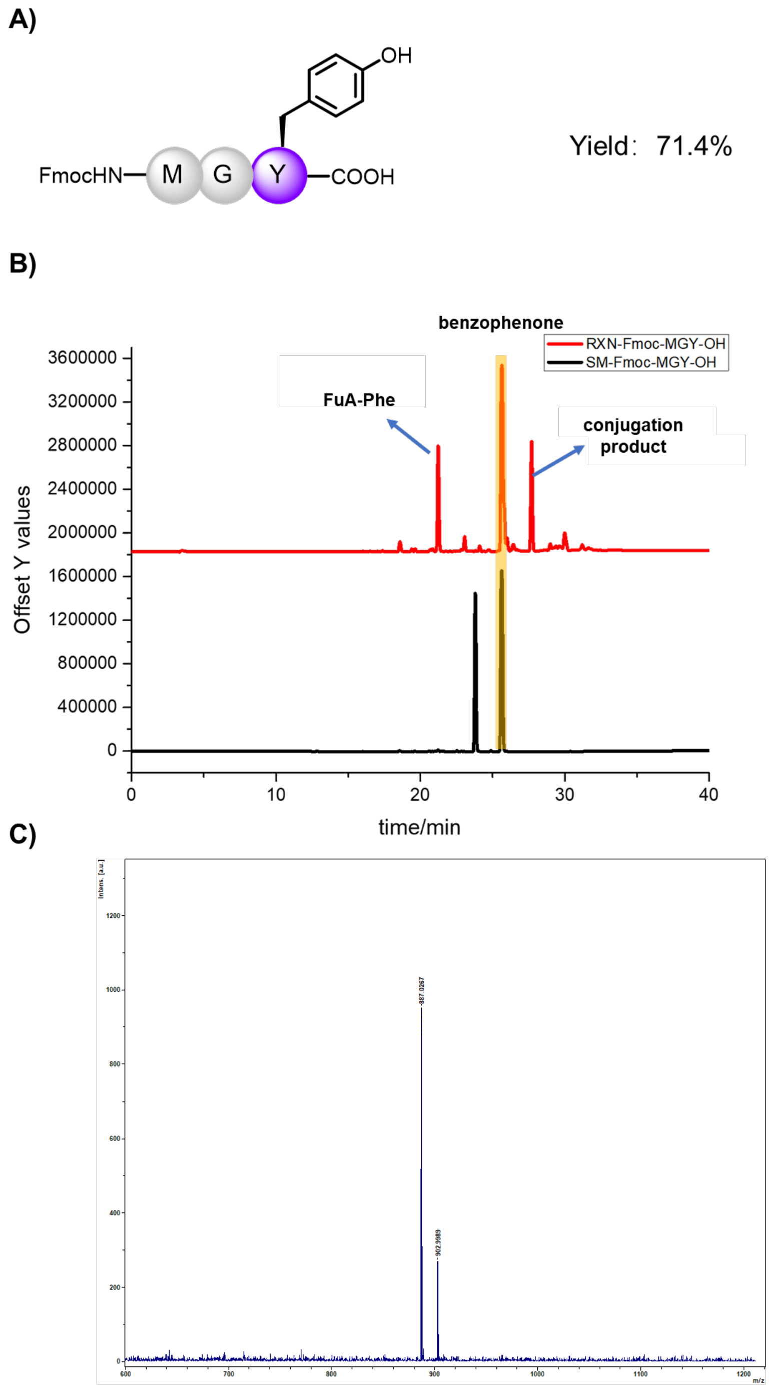

Figure S5. Reaction between Fmoc-MGY-OH and **FuA-Phe**. (**A**) Structure and reaction yield of Fmoc-MGY-OH. (**B**) HPLC spectrum of reaction of Fmoc-MGY-OH. (**C**) MALDI-TOF MS of new peaks calcd for $C_{46}H_{48}N_4NaO_{11}S^+$ $[M+Na]^+$ 887.2933, found 887.0267; calcd for $C_{46}H_{48}N_4KO_{11}S^+$ $[M+K]^+$ 903.2672, found 902.9989.

A)

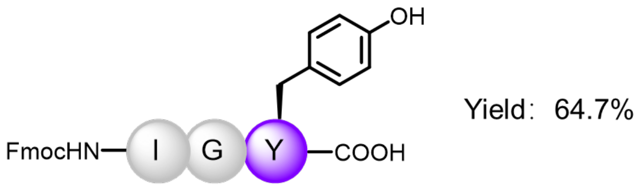

B)

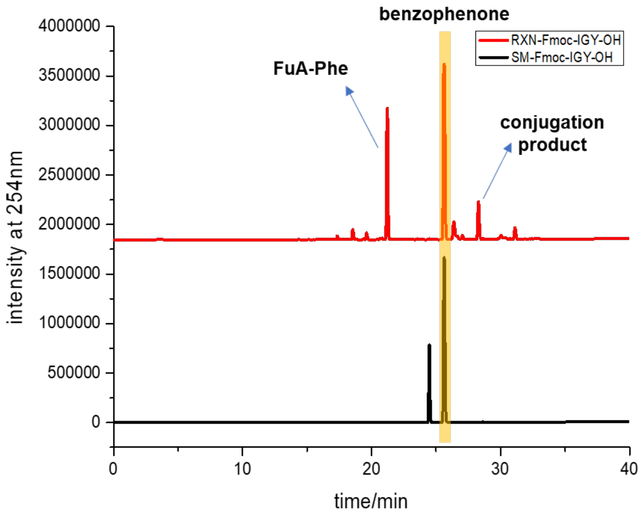

C)

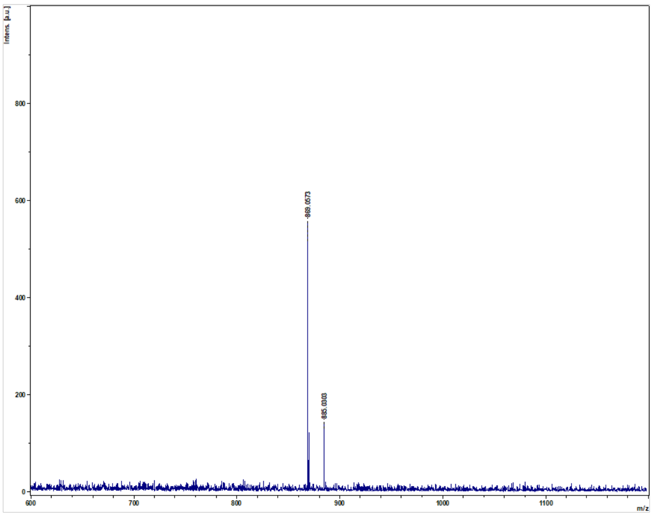

Figure S6. Reaction between Fmoc-IGY-OH and **FuA-Phe**. (**A**) Structure and reaction yield of Fmoc-IGY-OH. (**B**) HPLC spectrum of reaction of Fmoc-IGY-OH. (**C**) MALDI-TOF MS of new peaks calcd for $C_{47}H_{50}N_4NaO_{11}^+$ $[M+Na]^+$ 869.3368, found 869.0573; calcd for $C_{47}H_{50}N_4KO_{11}^+$ $[M+K]^+$ 885.3108, found 885.0303.

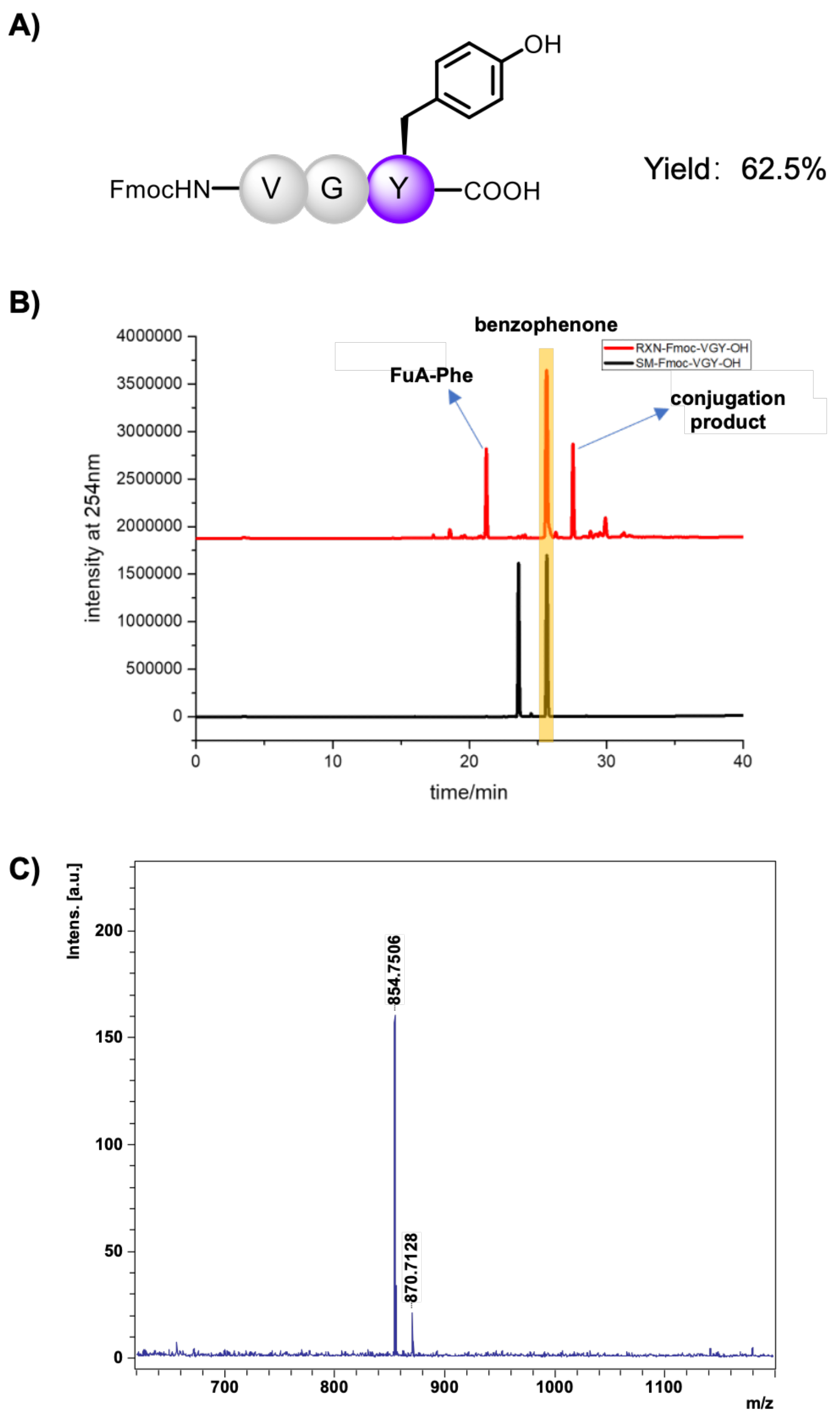

Figure S7. Reaction between Fmoc-VGY-OH and **FuA-Phe**. (**A**) Structure and reaction yield of Fmoc-VGY-OH. (**B**) HPLC spectrum of reaction of Fmoc-VGY-OH. (**C**) MALDI-TOF MS of new peaks calcd for $C_{46}H_{48}N_4NaO_{11}^+$ [M+Na]$^+$ 855.3212, found 854.7506; calcd for $C_{46}H_{48}N_4KO_{11}^+$ [M+K]$^+$ 871.2952, found 870.7128.

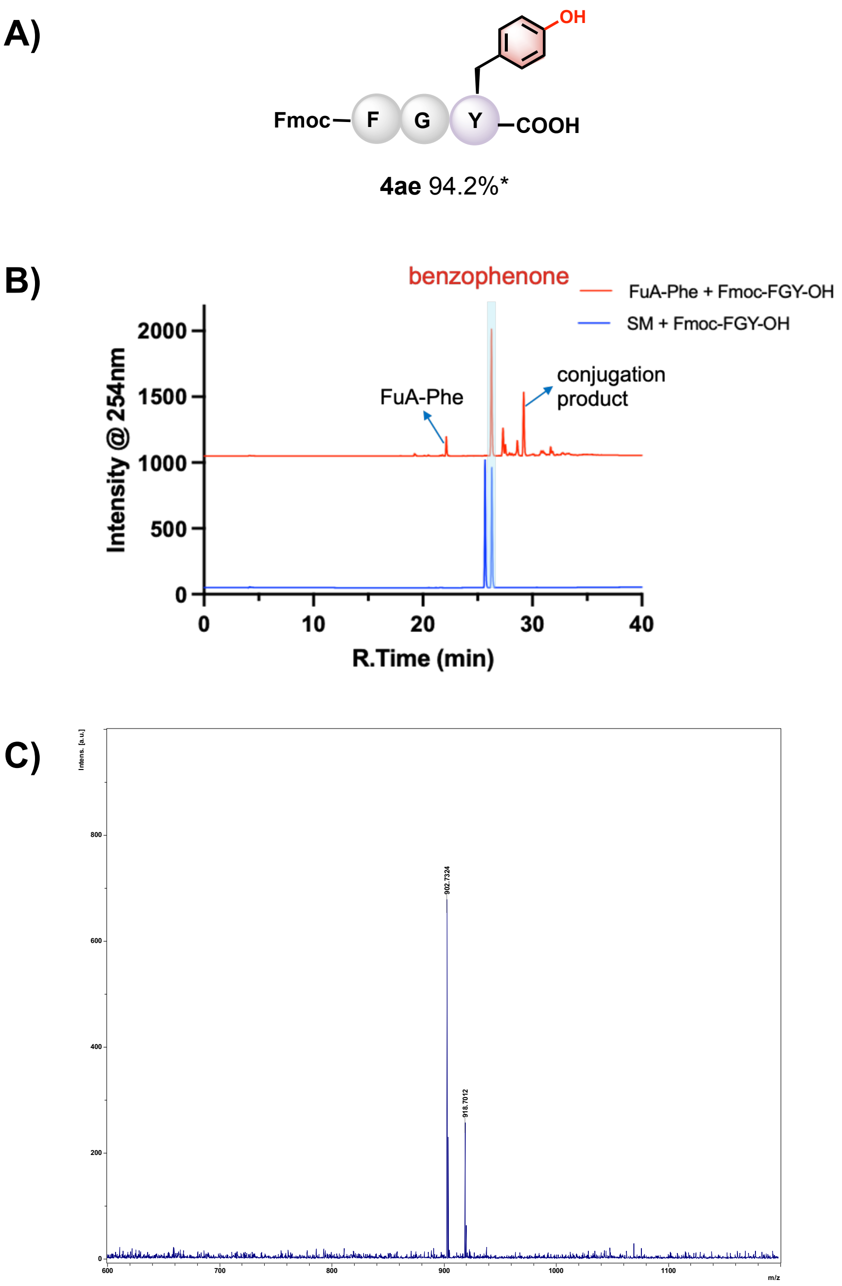

Figure S8. Reaction between Fmoc-FGY-OH and **FuA-Phe**. (**A**) Structure and reaction yield of Fmoc-FGY-OH. *, reaction with **FuA-Phe**. (**B**) HPLC spectrum of reaction of Fmoc-FGY-OH. (**C**) MALDI-TOF MS of new peaks calcd for $C_{50}H_{48}N_4NaO_{11}^+$ $[M+Na]^+$ 903.3212, found 902.7324; calcd for $C_{50}H_{48}N_4KO_{11}^+$ $[M+K]^+$ 919.2952, found 918.7012.

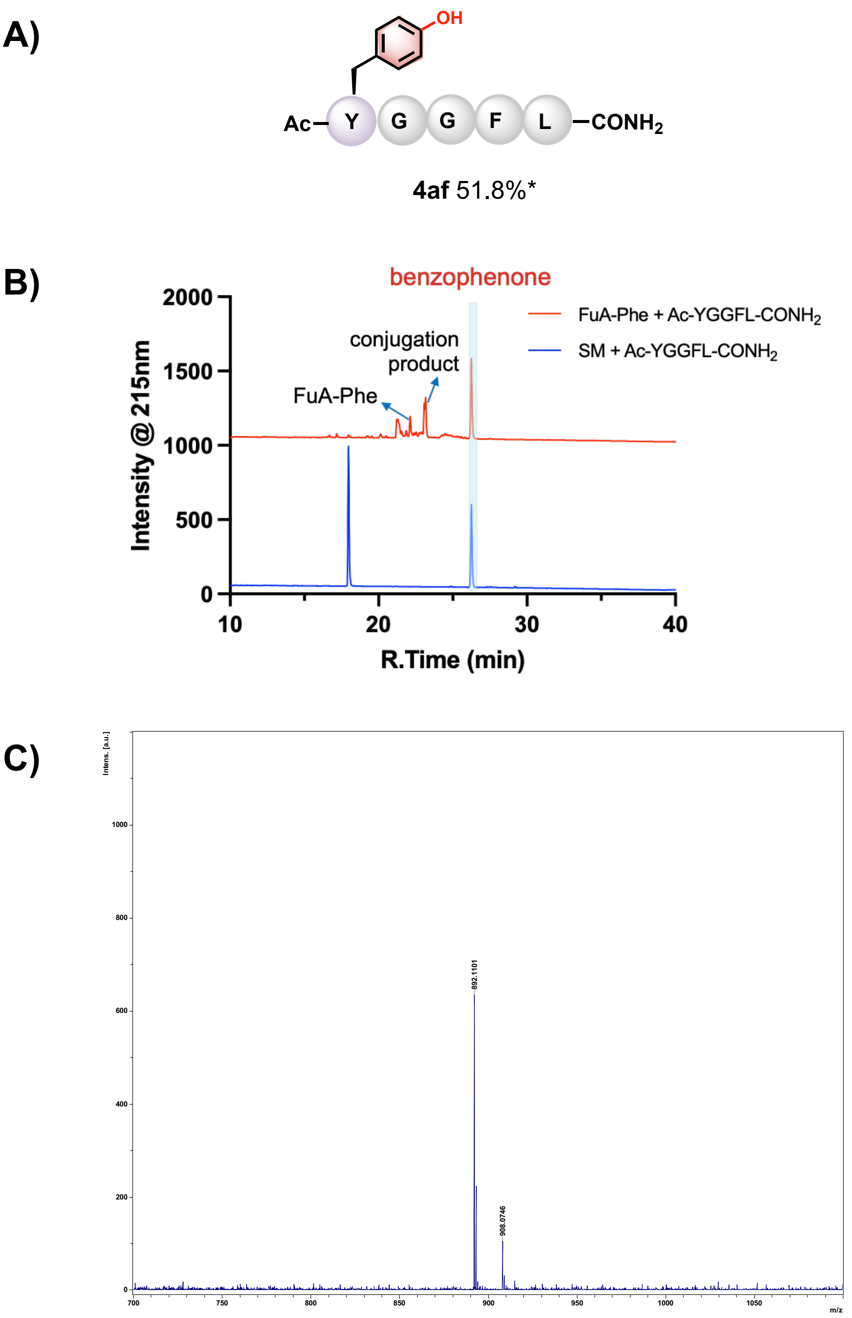

Figure S9. Reaction between Ac-YGGFL-CONH2 and **FuA-Phe**. (**A**) Structure and reaction yield of Ac-YGGFL-CONH2. (**B**) HPLC spectrum of reaction of Ac-YGGFL-CONH2. (**C**) MALDI-TOF MS of new peaks calcd for $C_{45}H_{55}N_7NaO_{11}^+$ $[M+Na]^+$ 892.3852, found 892.1101; $C_{45}H_{55}N_7KO_{11}^+$ $[M+K]^+$ 908.3592, found 908.0745.

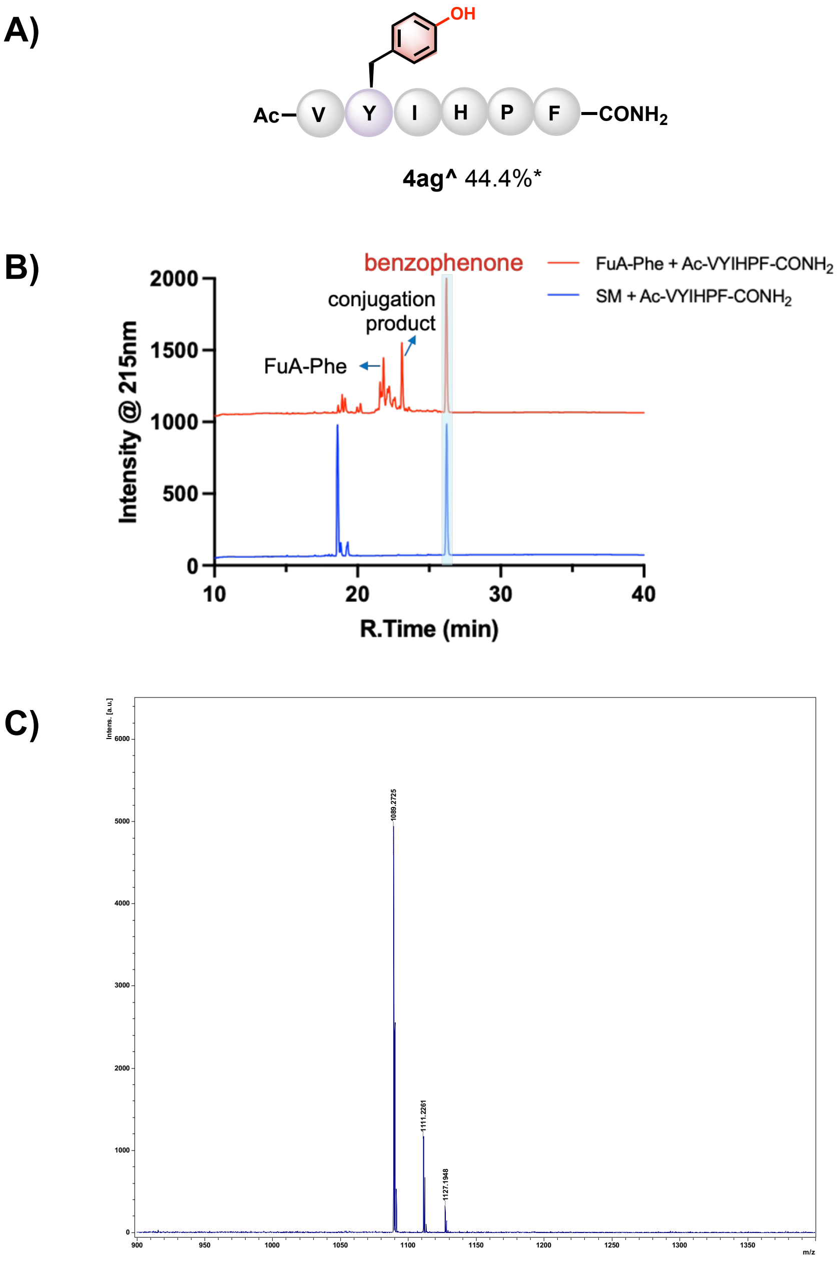

Figure S10. Reaction between Ac-VYIHPF-$CONH_2$ and **FuA-Phe**. (**A**) Structure and reaction yield of Ac-VYIHPF-$CONH_2$. ^, reaction for 12 h. (**B**) HPLC spectrum of reaction of Ac-VYIHPF-$CONH_2$. (**C**) MALDI-TOF MS of new peaks calcd for $C_{57}H_{73}N_{10}O_{11}^+$ $[M+H]^+$ 1089.5404, found 1089.2725; $C_{57}H_{72}N_{10}NaO_{11}^+$ $[M+Na]^+$ 1111.5223, found 1111.2261; $C_{57}H_{72}N_{10}KO_{11}^+$ $[M+K]^+$ 1127.4963, found 1127.1948.

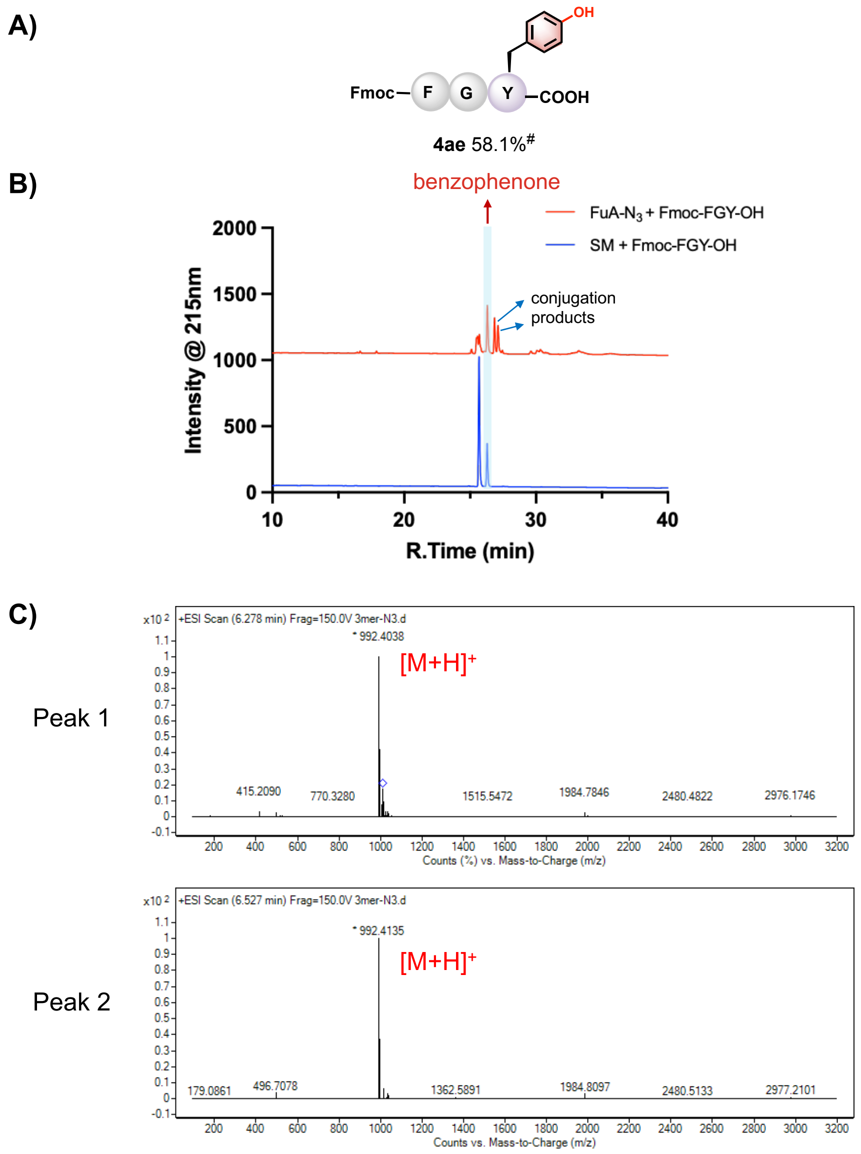

Figure S11. Reaction between Fmoc-FGY-OH and **FuA-PEG-N$_3$**. (**A**) Structure and reaction yield of Fmoc-FGY-OH. #, reaction with **FuA-PEG-N$_3$**. (**B**) HPLC spectrum of reaction of Fmoc-FGY-OH. (**C**) ESI MS of peak 1 calcd for $C_{51}H_{58}N_7O_{14}^+$ $[M+H]^+$ 992.4036, found 992.4038; ESI MS of peak 2 calcd for $C_{51}H_{58}N_7O_{14}^+$ $[M+H]^+$ 992.4036, found 992.4135.

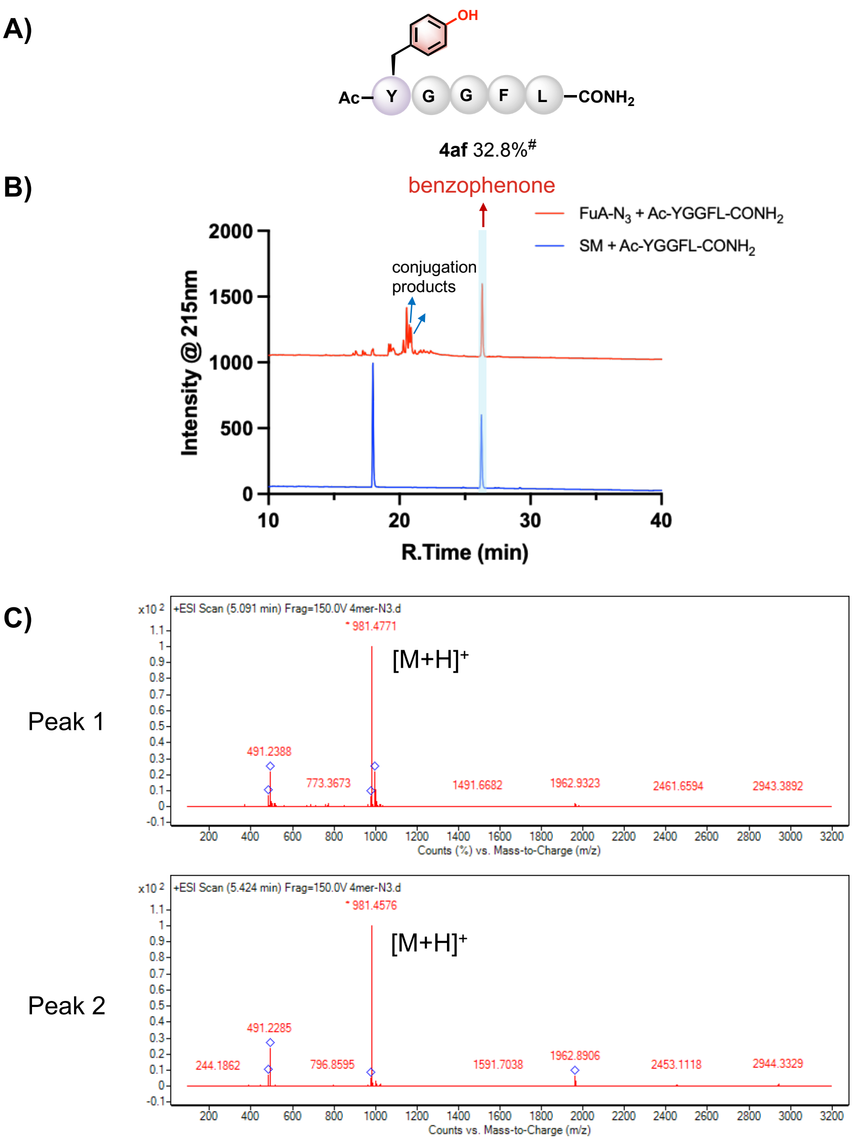

Figure S12. Reaction between Ac-YGGFL-$CONH_2$ and **FuA-PEG-N_3**. (**A**) Structure and reaction yield of Ac-YGGFL-$CONH_2$. (**B**) HPLC spectrum of reaction of Ac-YGGFL-$CONH_2$. (**C**) ESI MS of peak 1 calcd for $C_{46}H_{65}N_{10}O_{14}^+$ $[M+H]^+$ 981.4676, found 981.4771; ESI MS of peak 2 calcd for $C_{46}H_{65}N_{10}O_{14}^+$ $[M+H]^+$ 981.4676, found 981.4576.

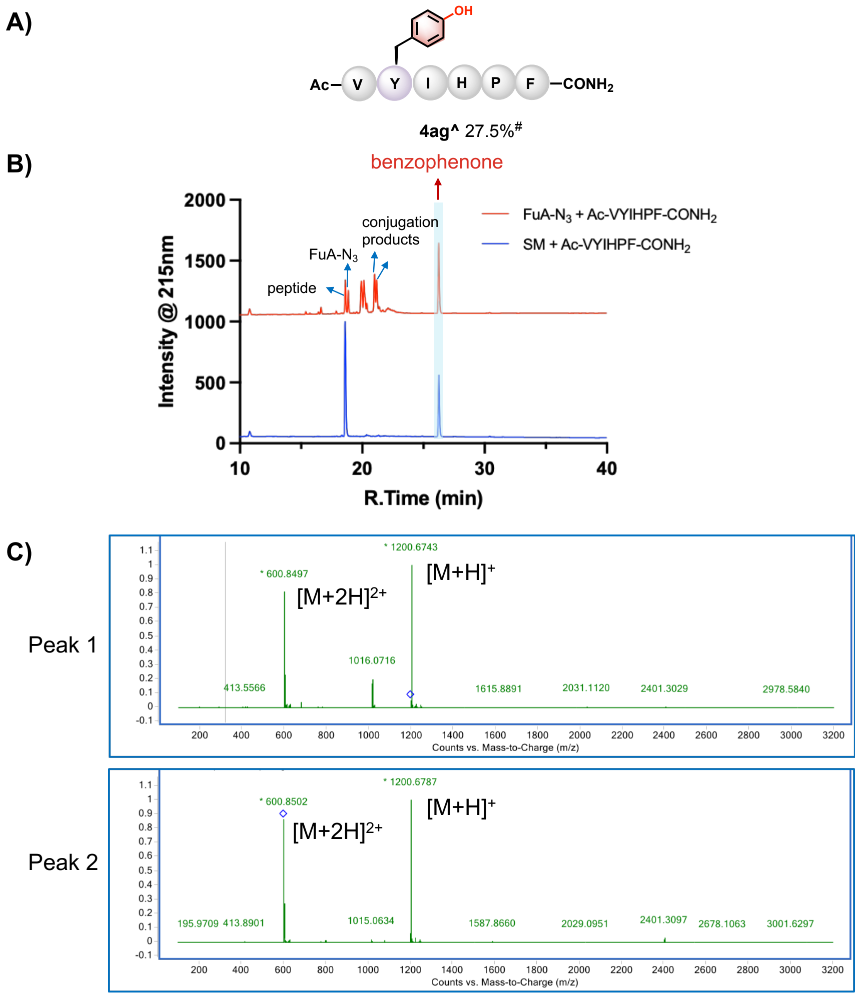

Figure S13. Reaction between Ac-VYIHPF-CONH2 and **FuA-PEG-N3**. (**A**) Structure and reaction yield of Ac-VYIHPF-CONH2. ^, reaction for 12 h. (**B**) HPLC spectrum of reaction of Ac-VYIHPF-CONH2. (**C**) ESI MS of peak 1 calcd for $C_{58}H_{82}N_{13}O_{15}^{+}$ $[M+H]^{+}$ 1200.6048, found 1200.6743; $[M+2H]^{2+}$ 600.8024, found 600.8497. ESI MS of peak 2 calcd for $C_{58}H_{82}N_{13}O_{15}^{+}$ $[M+H]^{+}$ 1200.6048, found 1200.6787; $[M+2H]^{2+}$ 600.8024, found 600.8502.

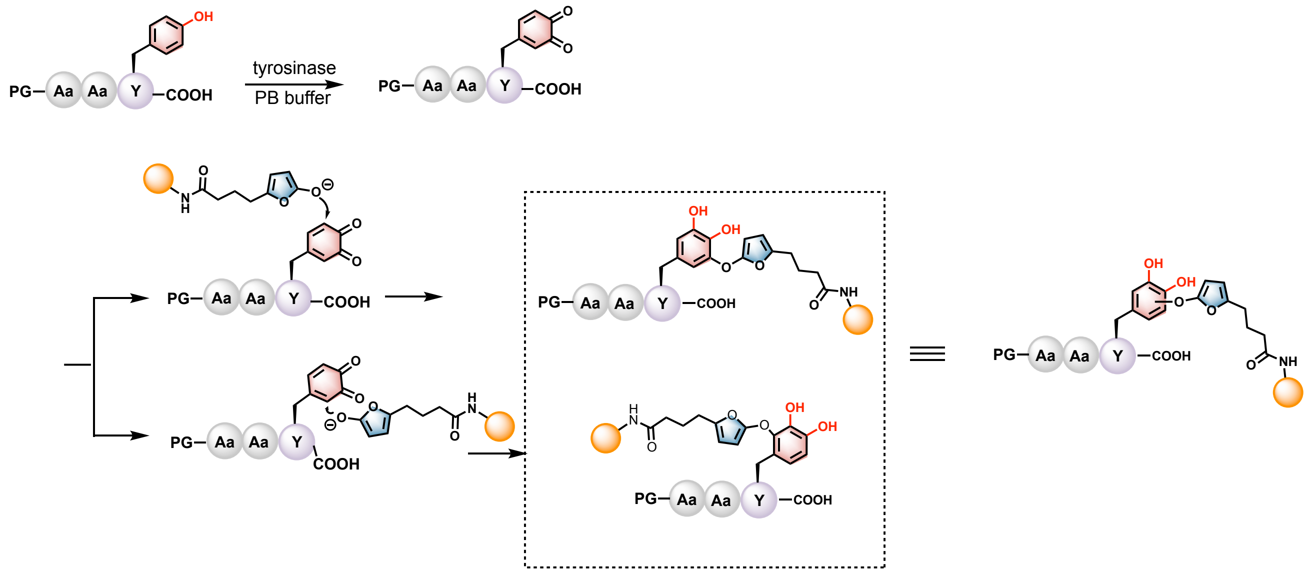

Figure S14. Reaction pathways leading to two configurational isomers.

A)

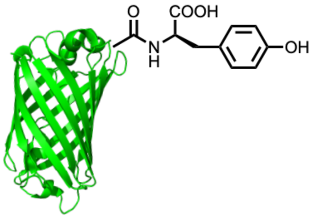

GFP-GGY

protein sequences:

MHHHHHHMASMTGGQQMGRGSMVSKGEELFTGVVPILVEL
DGDVNGHKFSVSGEGEGDATYGKLTLKFICTTGKLPVPWPTL
VTTLTYGVQCFSRYPDHMKQHDFFKSAMPEGYVQERTIFFKD
DGNYKTRAEVKFEGDTLVNRIELKGIDFKEDGNILGHKLEYN
YNSHNVYIMADKQKNGIKVNFKIRHNIEDGSVQLADHYQQN
TPIGDGPVLLPDNHYLSTQSALSKDPNEKRDHMVLLEFVTAA
GITLGMDELYKELRRQASGGGGSGGY*

B)

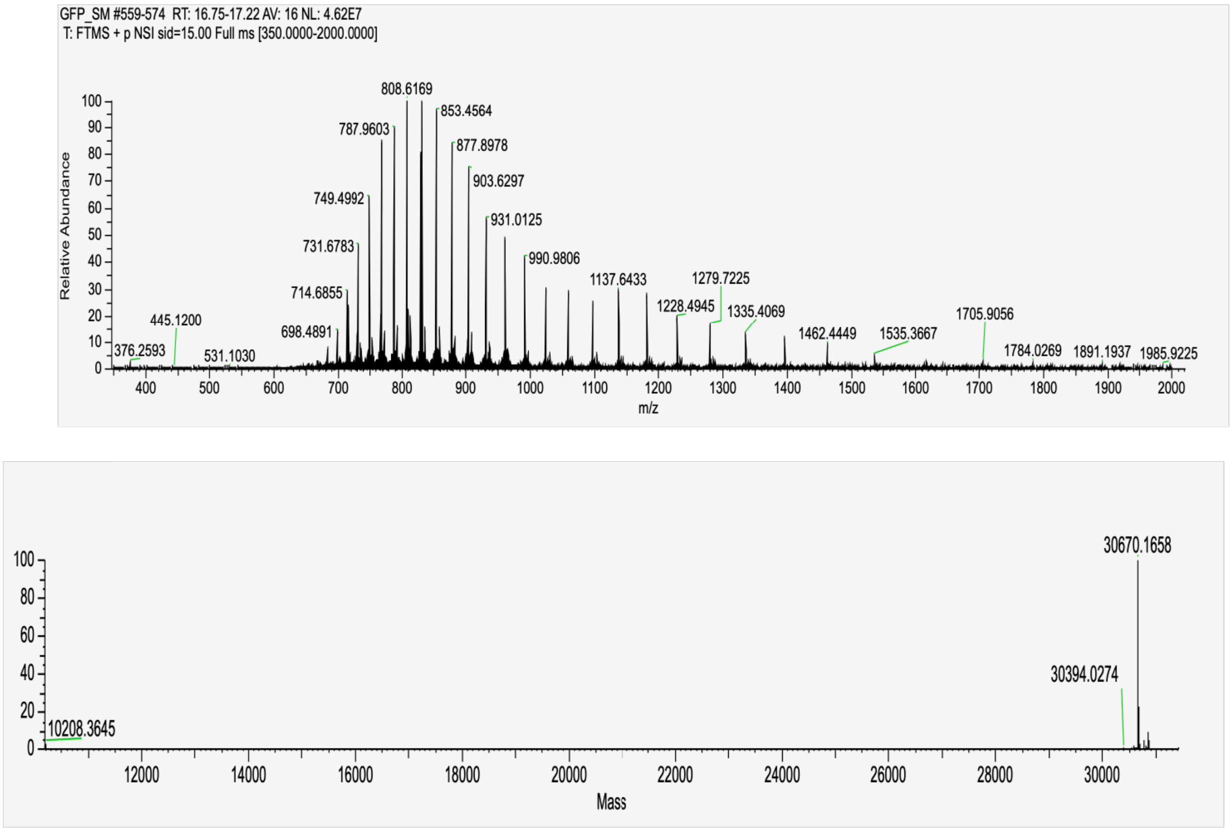

Figure S15. Characterization of GFP-GGY. (**A**) Protein sequence of GFP-GGY. (**B**) LC-MS analysis of GFP-GGY protein. Calculated 30670 Da, found 30670 Da.

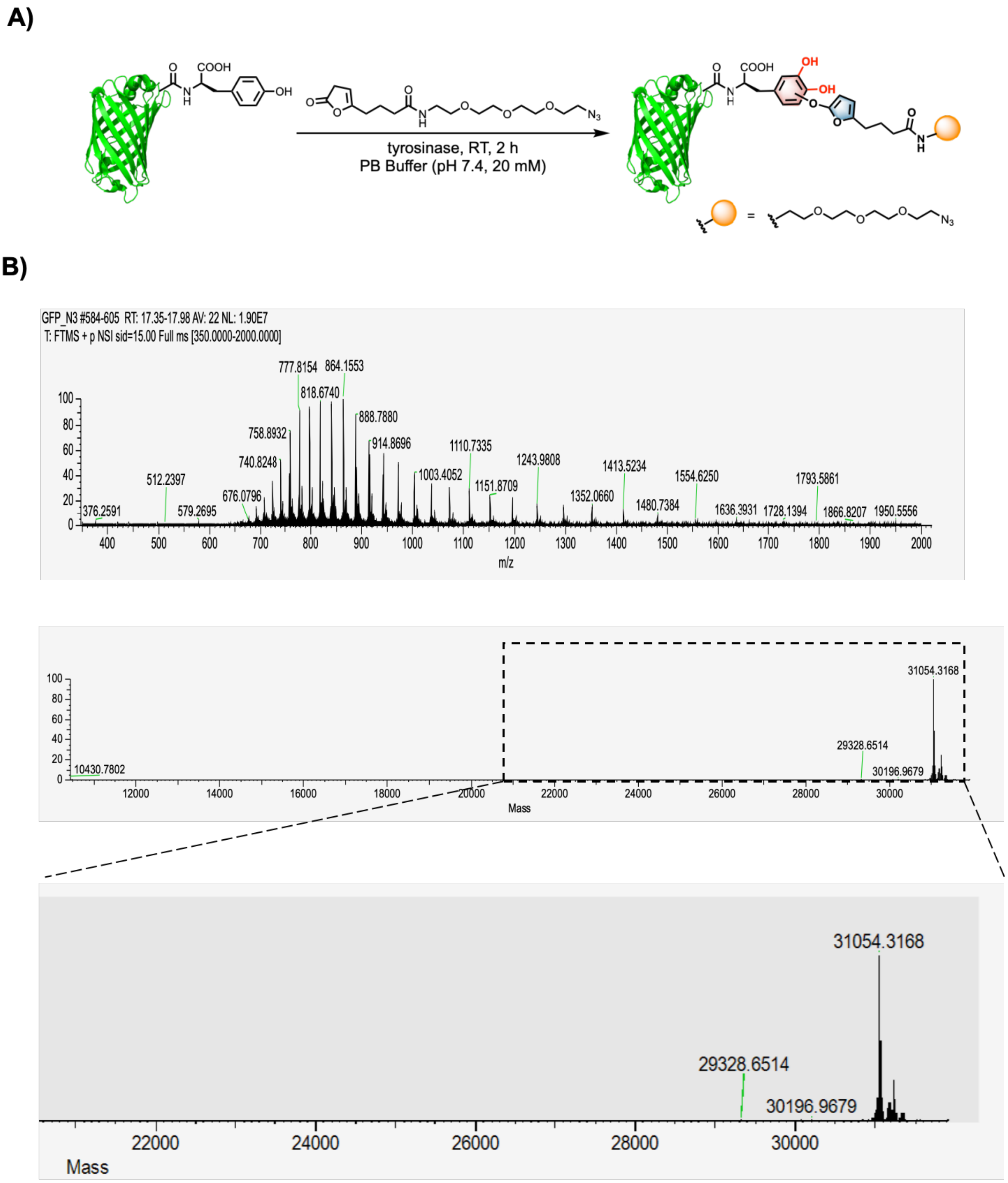

Figure S16. Reaction of GFP-GGY and **FuA-PEG-N_3**. (**A**) Scheme of GFP-GGY modification with FuA-N_3. (**B**)LC-MS analysis of modified GFP-GGY protein. Calculated 31054 Da, found 31054 Da.

A)

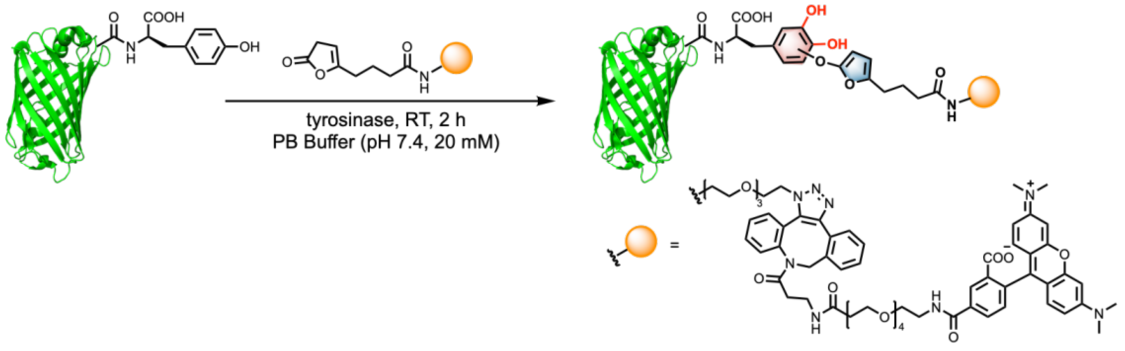

B)

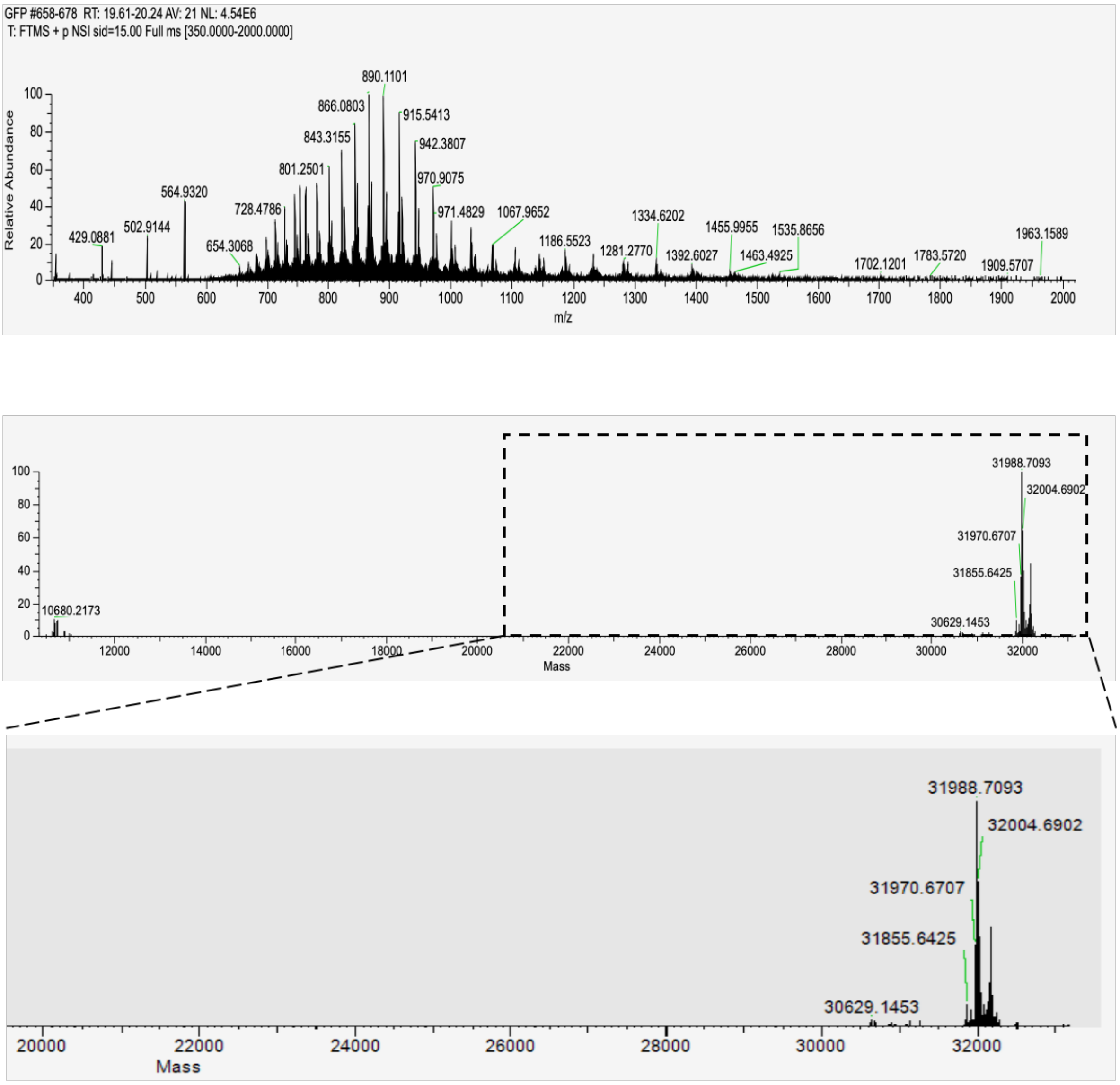

Figure S17. Reaction of GFP-GGY with **FuA-TAM**. (**A**) Scheme of GFP-GGY modification with FuA-TAM. (**B**) LC-MS analysis of modified GFP-GGY protein. Calculated 31990 Da, found 31989 Da.

A)

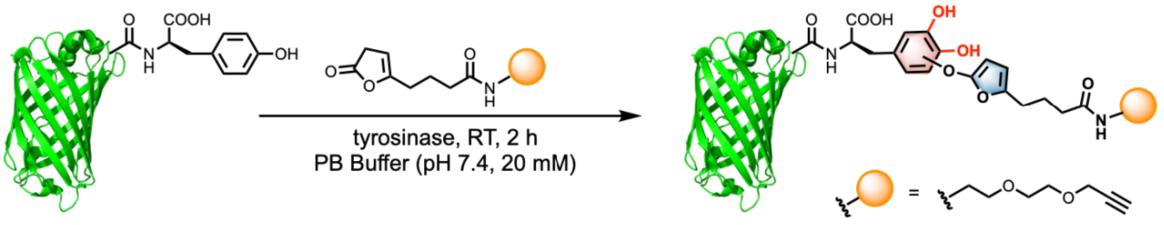

B)

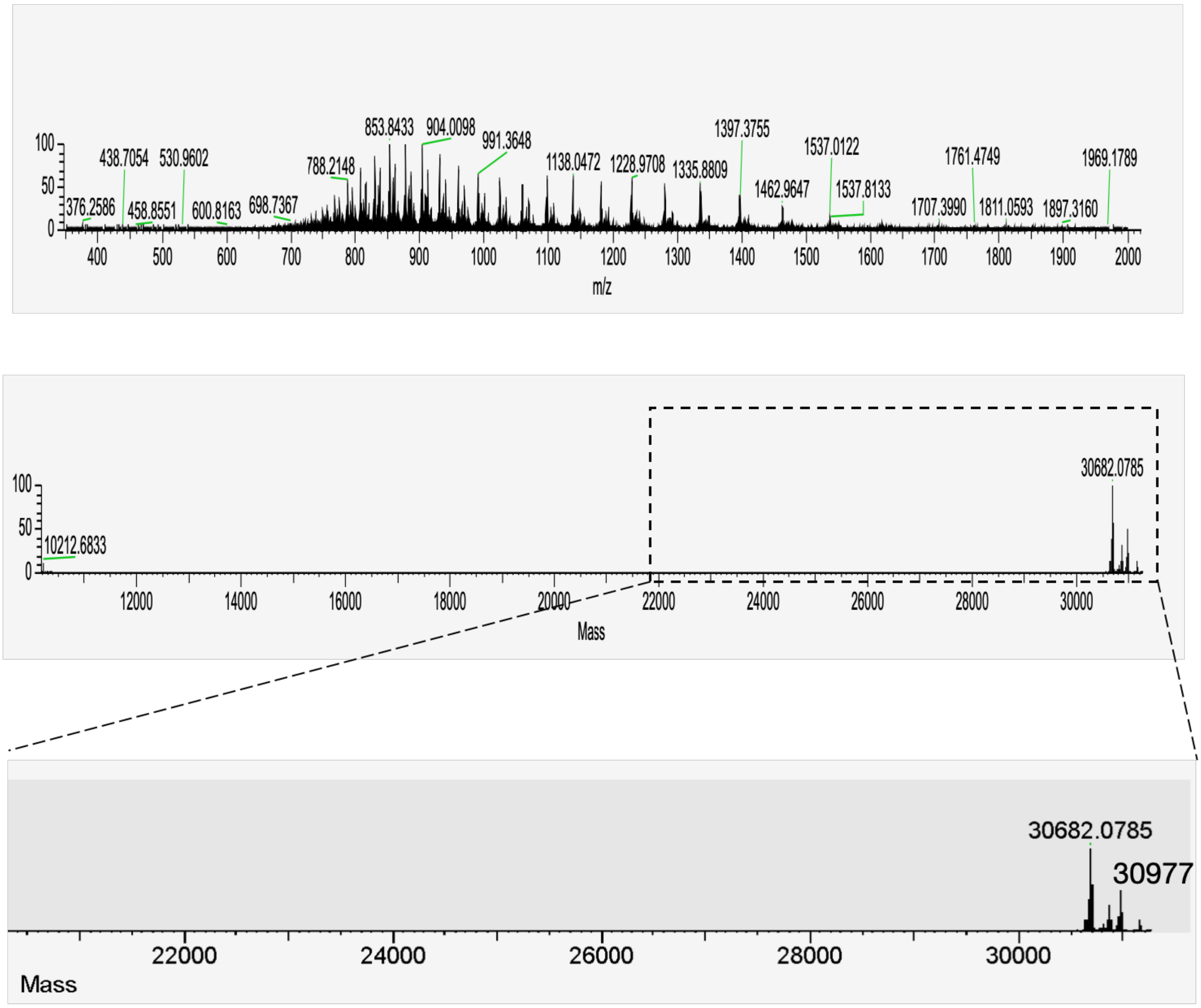

Figure S18. Reaction of GFP-GGY with **FuA-PEG-Alkyne**. (**A**) Scheme of GFP-GGY modification with FuA-PEG-Alkyne. (**B**) LC-MS analysis of modified GFP-GGY protein. Calculated 30979 Da, found 30977 Da.

A)

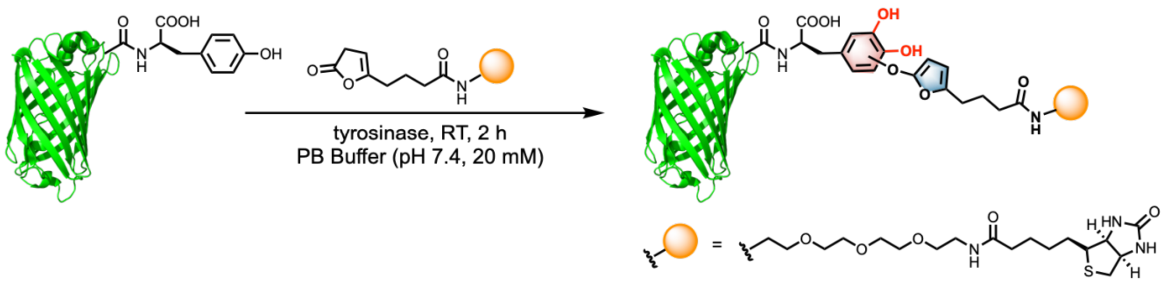

B)

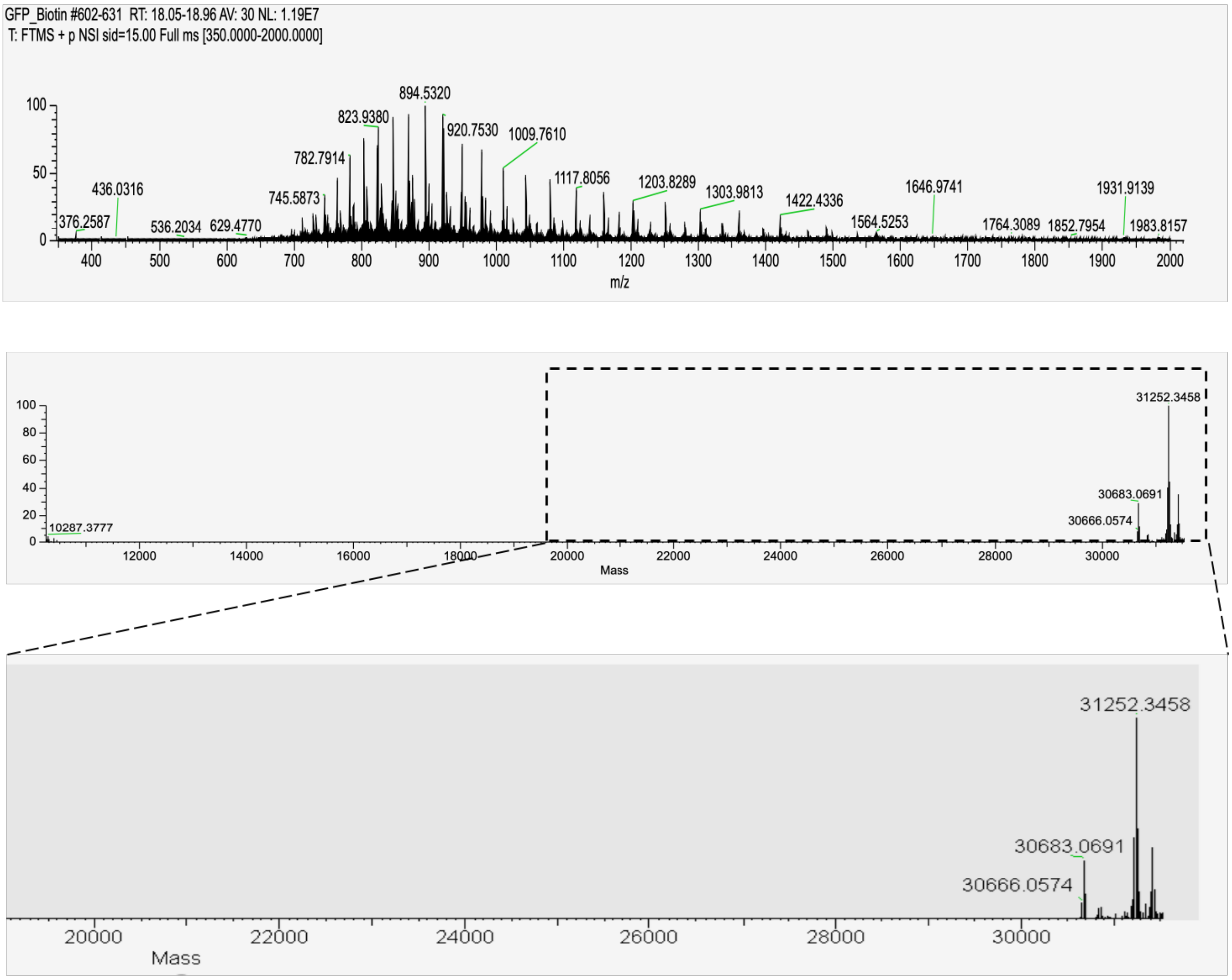

Figure S19. Reaction of GFP-GGY and **FuA-PEG-biotin**. (**A**) Scheme of GFP-GGY modification with FuA-PEG-Biotin. (**B**)LC-MS analysis of modified GFP-GGY protein. Calculated 31254 Da, found 31252 Da.

A)

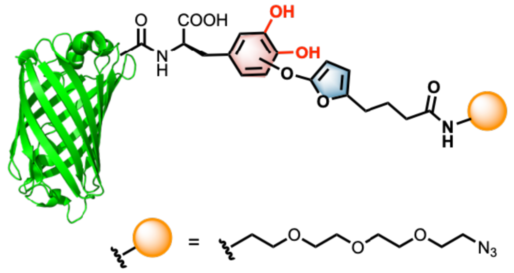

GFP-GGY-FuA-PEG-N3

B)

Peptide Summary

Fragment Matches

Value Type: Theo. Mass [Da]

Ion Series | Neutral Losses | Precursor Ions | Internal Fragments

#1	b⁺	b²⁺	Seq.	y⁺	y²⁺	#2
1	129.06585	65.03657	Q			11
2	200.10297	100.55512	A	1153.47562	577.24145	10
3	287.13500	144.07114	S	1082.43851	541.72289	9
4	344.15646	172.58187	G	995.40648	498.20688	8
5	401.17792	201.09260	G	938.38502	469.69615	7
6	458.19939	229.60333	G	881.36355	441.18542	6
7	515.22085	258.11406	G	824.34209	412.67468	5
8	602.25288	301.63008	S	767.32063	384.16395	4
9	659.27434	330.14081	G	680.28860	340.64794	3
10	716.29581	358.65154	G	623.26713	312.13721	2
11			Y-CUHK-H...	566.24567	283.62647	1

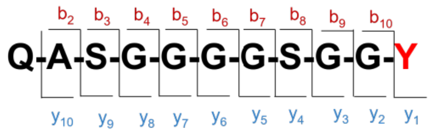

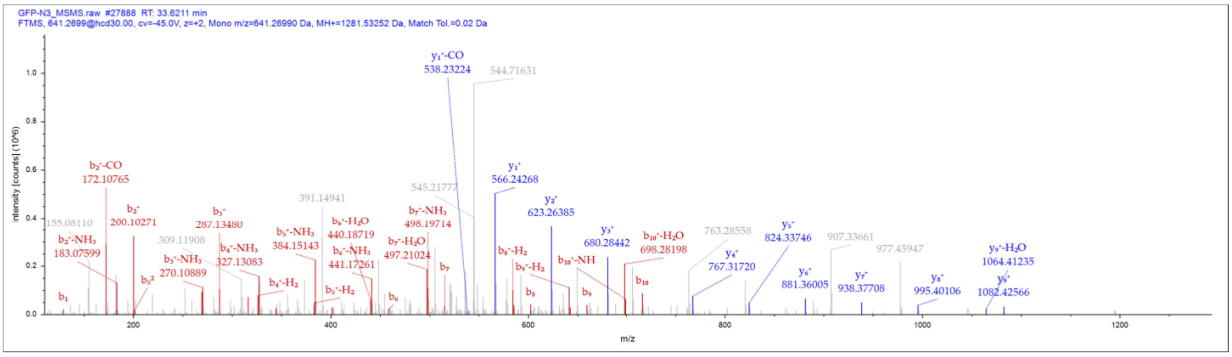

Figure S20. LC-MS/MS characterization of GFP-GGY/**FuA-PEG-N$_3$** conjugate. (**A**) Structure of GFP-GGY modification with **FuA-PEG-N$_3$**. (**B**) LC-MS/MS analysis of the protein conjugate.

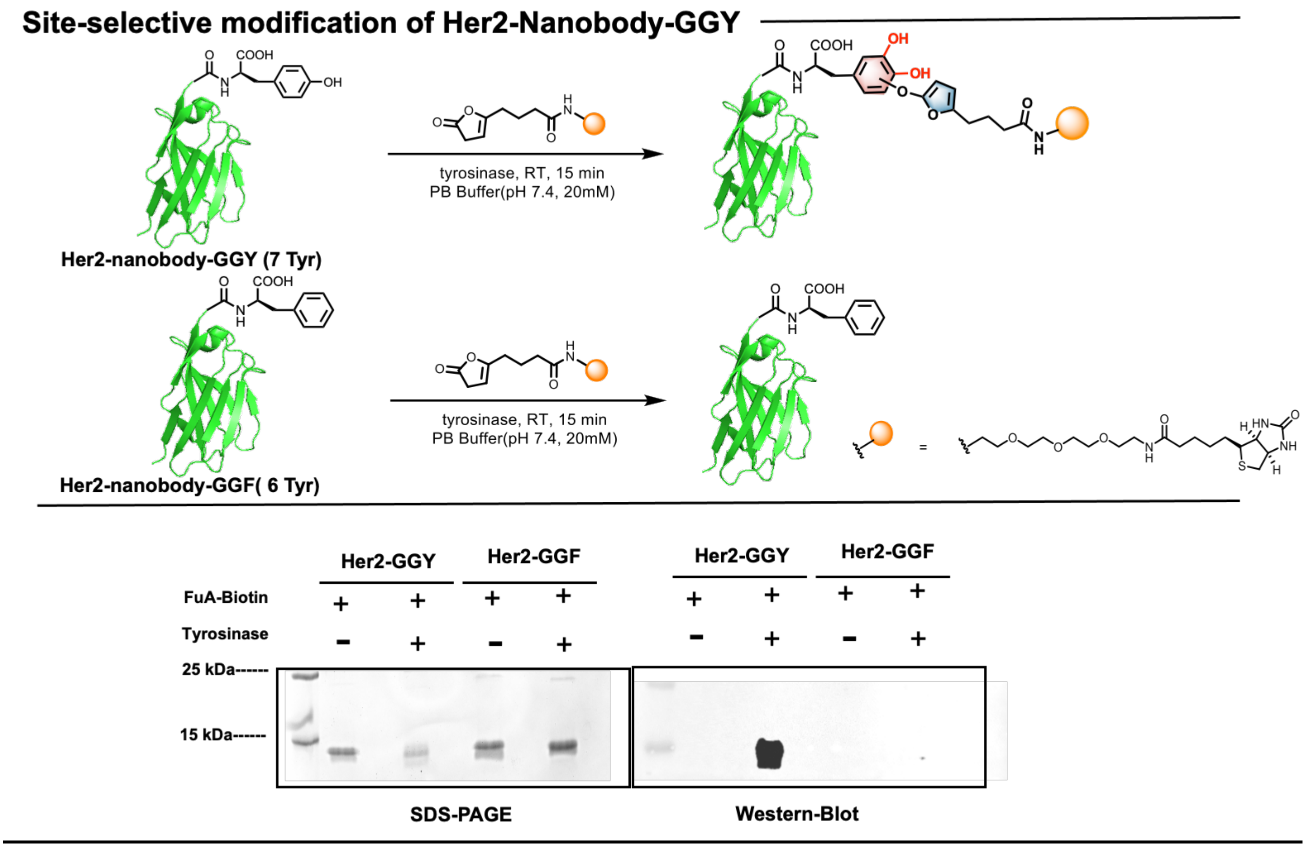

Figure S21. Reaction of HER2-nanobodies. HER2-GGY or HER2-GGF nanobody (1 *eq*.) was incubated with tyrosinase (0.01 *eq*.) and different **FuA-PEG-biotin** (100 eq. to total protein) in PB buffer (pH 7.4) at room temperature for 15min. The result was analyzed by SDS-PAGE and Western-blot.

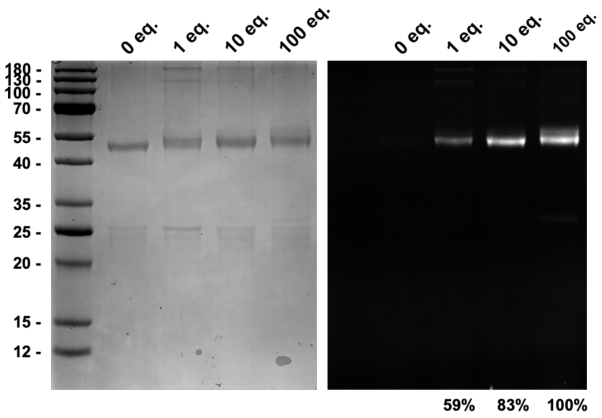

Figure S22. The reaction of atezolizumab (a humanized IgG1 monoclonal antibody) with different concentrations of **FuA-PEG-N_3**.

A)

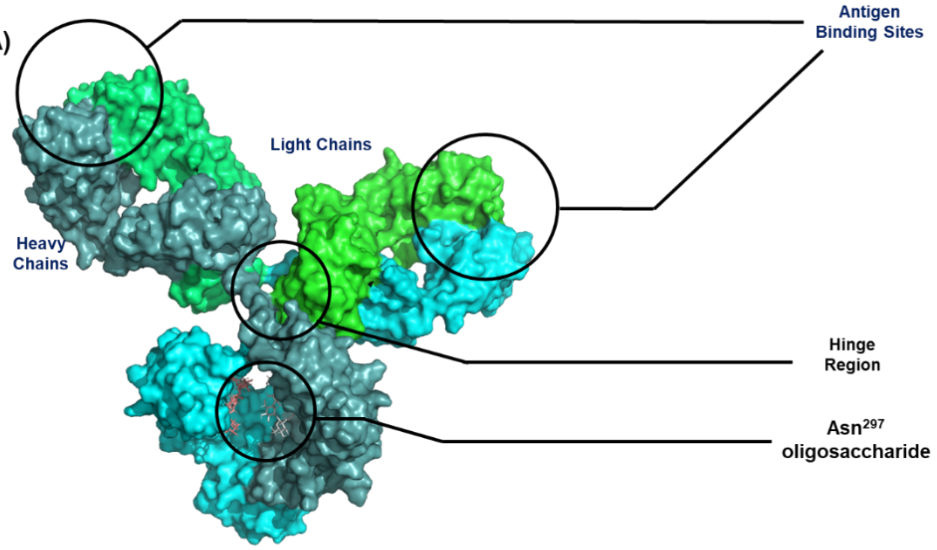

B)

Trastuzumab:
Heavy chain:

```
1                                                           60
EVQLVESGGG LVQPGGSLRL SCAASGFNIK DTYIHWVRQA PGKGLEWVAR IYPTNGYTRY
61                                                         120
ADSVKGRFTI SADTSKNTAY LQMNSLRAED TAVYYCSRWG GDGFYAMDYW GQGTLVTVSS
121                                                        180
ASTKGPSVFP LAPSSKSTSG GTAALGCLVK DYFPEPVTVS WNSGALTSGV HTFPAVLQSS
181                                                        240
GLYSLSSVVT VPSSSLGTQT YICNVNHKPS NTKVDKKVEP KSCDKTHTCP PCPAPELLGG
241                                                        300
PSVFLFPPKP KDTLMISRTP EVTCVVVDVS HEDPEVKFNW YVDGVEVHNA KTKPREEQYN
301                                                        360
STYRVVSVLT VLHQDWLNGK EYKCKVSNKA LPAPIEKTIS KAKGQPREPQ VYTLPPSREE
361                                                        420
MTKNQVSLTC LVKGFYPSDI AVEWESNGQP ENNYKTTPPV LDSDGSFFLY SKLTVDKSRW
421
QQGNVFSCSV MHEALHNHYT QKSLSLSPG
```

C)

Atezolizumab:
Heavy chain:

```
1                                                           60
EVQLVESGGG LVQPGGSLRL SCAASGFTFS DSWIHWVRQA PGKGLEWVAW ISPYGGSTYY
61                                                         120
ADSVKGRFTI SADTSKNTAY LQMNSLRAED TAVYYCARRH WPGGFDYWGQ GTLVTVSSAS
121                                                        180
TKGPSVFPLA PSSKSTSGGT AALGCLVKDY FPEPVTVSWN SGALTSGVHT FPAVLQSSGL
181                                                        240
YSLSSVVTVP SSSLGTQTYI CNVNHKPSNT KVDKKVEPKS CDKTHTCPPC PAPELLGGPS
241                                                        300
VFLFPPKPKD TLMISRTPEV TCVVVDVSHE DPEVKFNWYV DGVEVHNAKT KPREEQYAST
301                                                        360
YRVVSVLTVL HQDWLNGKEY KCKVSNKALP APIEKTISKA KGQPREPQVY TLPPSREEMT
361                                                        420
KNQVSLTCLV KGFYPSDIAV EWESNGQPEN NYKTTPPVLD SDGSFFLYSK LTVDKSRWQQ
421
GNVFSCSVMH EALHNHYTQK SLSLSPGK
```

D)

Daratumumab:
Heavy chain:

1 60
EVQLLESGGG LVQPGGSLRL SCAVSGFTFN SFAMSWVRQA PGKGLEWVSA ISGSGGGTYY
61 120
ADSVKGRFTI SRDNSKNTLY LQMNSLRAED TAVYFCAKDK ILWFGEPVFD YWGQGTLVTV
121 180
SSASTKGPSV FPLAPSSKST SGGTAALGCL VKDYFPEPVT VSWNSGALTS GVHTFPAVLQ
181 240
SSGLYSLSSV VTVPSSSLGT QTYICNVNHK PSNTKVDKRV EPKSCDKTHT CPPCPAPELL
241 300
GGPSVFLFPP KPKDTLMISR TPEVTCVVVD VSHEDPEVKF NWYVDGVEVH NAKTKPREEQ
301 360
YNSTYRVVSV LTVLHQDWLN GKEYKCKVSN KALPAPIEKT ISKAKGQPRE PQVYTLPPSR
361 420
EEMTKNQVSL TCLVKGFYPS DIAVEWESNG QPENNYKTTP PVLDSDGSFF LYSKLTVDKS
421
RWQQGNVFSC SVMHEALHNH YTQKSLSLSP GK

E)

Cetuximab:
Heavy chain:

1 60
QVQLKQSGPG LVQPSQSLSI TCTVSGFSLT NYGVHWVRQS PGKGLEWLGV IWSGGNTDYN
61 120
TPFTSRLSIN KDNSKSQVFF KMNSLQSNDT AIYYCARALT YYDYEFAYWG QGTLVTVSAA
121 180
STKGPSVFPL APSSKSTSGG TAALGCLVKDYFPEPVTVSW NSGALTSGVH TFPAVLQSSG
181 240
LYSLSSVVTV PSSSLGTQTY ICNVNHKPSN TKVDKRVEPK SCDKTHTCPP CPAPELLGGP
241 300
SVFLFPPKPK DTLMISRTPE VTCVVVDVSH EDPEVKFNWY VDGVEVHNAK TKPREEQYNS
301 360
TYRVVSVLTV LHQDWLNGKE YKCKVSNKAL PAPIEKTISK AKGQPREPQV YTLPPSREEM
361 420
TKNQVSLTCL VKGFYPSDIA VEWESNGQPE NNYKTTPPVL DSDGSFFLYS KLTVDKSRWQ
421
QGNVFSCSVM HEALHNHYTQ KSLSLSPGK

Figure S23. Sequence comparison of human IgG1 heavy chains. (**A**) Structure of human IgG1. (**B**) Heavy chain sequence of Trastuzumab. (**C**) Heavy chain sequence of Atezolizumab. (**D**) Heavy chain sequence of Daratumumab. (**E**) Heavy chain sequence of Cetuximab.

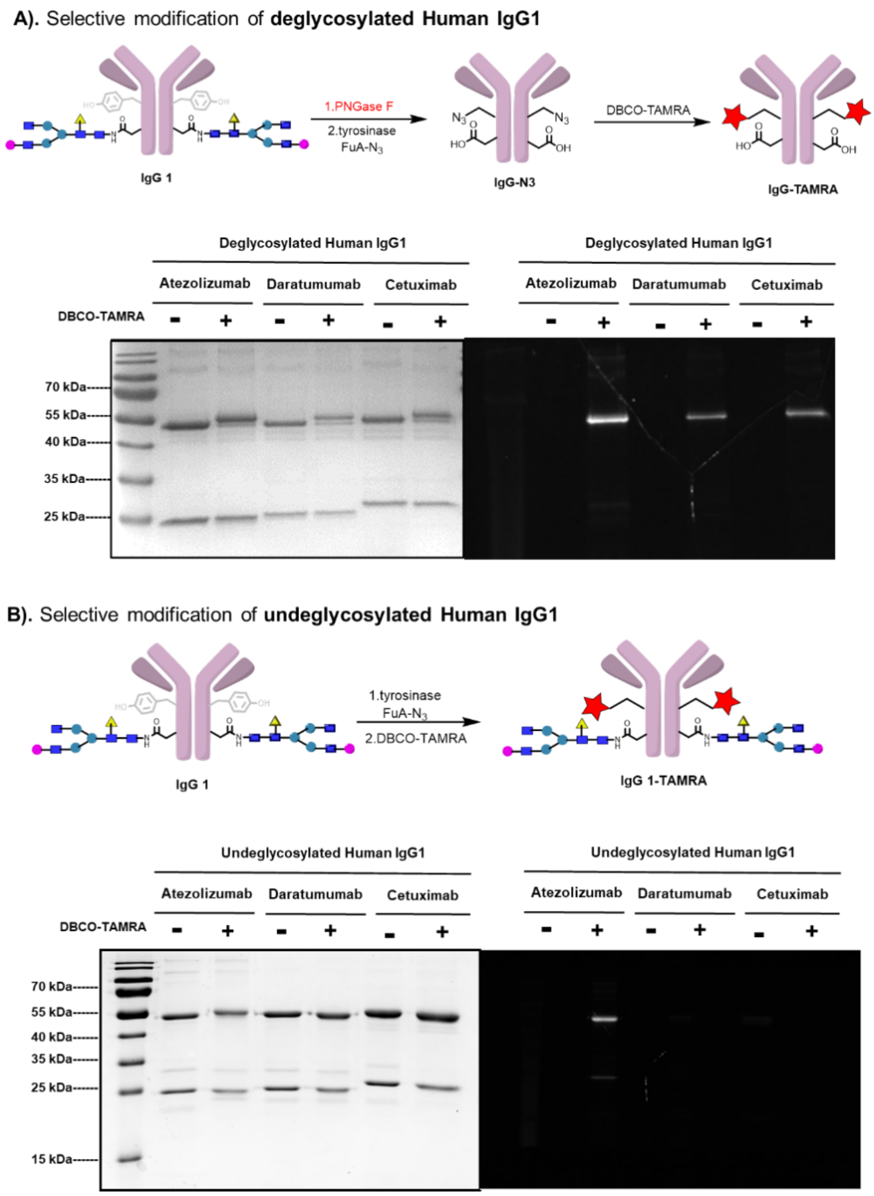

Figure S24. Reactions of Human IgG1s. (**A**) Selective modification of deglycosylated Human IgG1. (**B**) Selective modification of undeglycosylated Human IgG1. Reaction condition: 5.0 μM IgG (deglycosylated or undeglycosylated), 500 μM **FuA-PEG-N$_3$**, 2.0 μM Tyrosinase, PB buffer (0.2 M, pH 7.4), at 4 °C for 8 h. The reactions were quenched by 1% sodium dodecyl-sulfate (SDS), and DBCO-PEG$_4$-TAMRA was added at room temperature for 1.5 h. The solutions were then resolved by denaturing SDS−polyacrylamide gel electrophoresis (SDS-PAGE) and imaged by in-gel fluorescence scanning and Coomassie blue staining.

A)

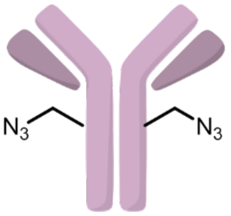

Atezo-FuA-PEG-N3

B)

Peptide Summary

Fragment Matches

Value Type: Theo. Mass [Da]

Ion Series | Neutral Losses | Precursor Ions | Internal Fragments

#1	b+	b2+	Seq.	y+	y2+	#2
1	130.04987	65.52857	E			9
2	259.09246	130.04987	E	1401.62810	701.31769	8
3	387.15104	194.07916	Q	1272.58551	636.79639	7
4	934.37887	467.69307	Y-CUHK-H...	1144.52693	572.76710	6
5	1005.41598	503.21163	A	597.29910	299.15319	5
6	1092.44801	546.72764	S	526.26199	263.63463	4
7	1193.49569	597.25148	T	439.22996	220.11862	3
8	1356.55902	678.78315	Y	338.18228	169.59478	2
9			R	175.11895	88.06311	1

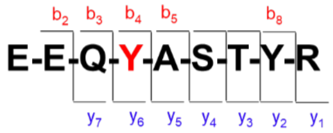

Tyr 296 of Atezolizumab is modified

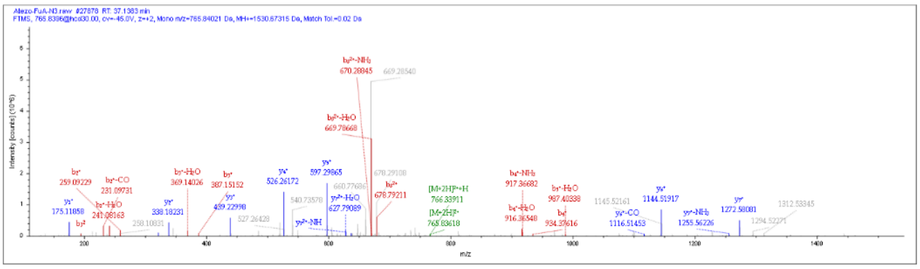

Figure S25. LC-MS/MS characterization of reaction site of Atezo/**FuA-PEG-N$_3$** conjugate. (**A**) Structure of Atezo modification with FuA-PEG-N$_3$. (**B**) LC-MS/MS analysis of the Atezolizumab conjugate.

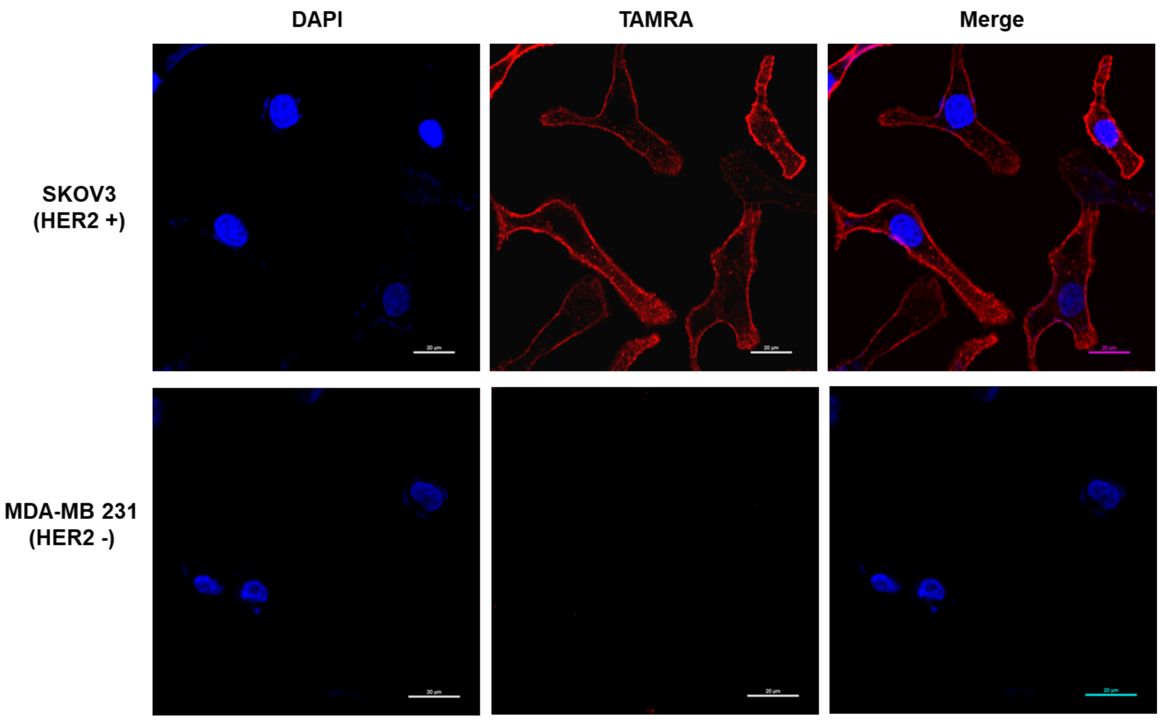

Figure S26. Confocal images of SKOV3 cells and MDA-MB 231 cells incubated with Tras-TAMRA. Scale bar, 20 μm.

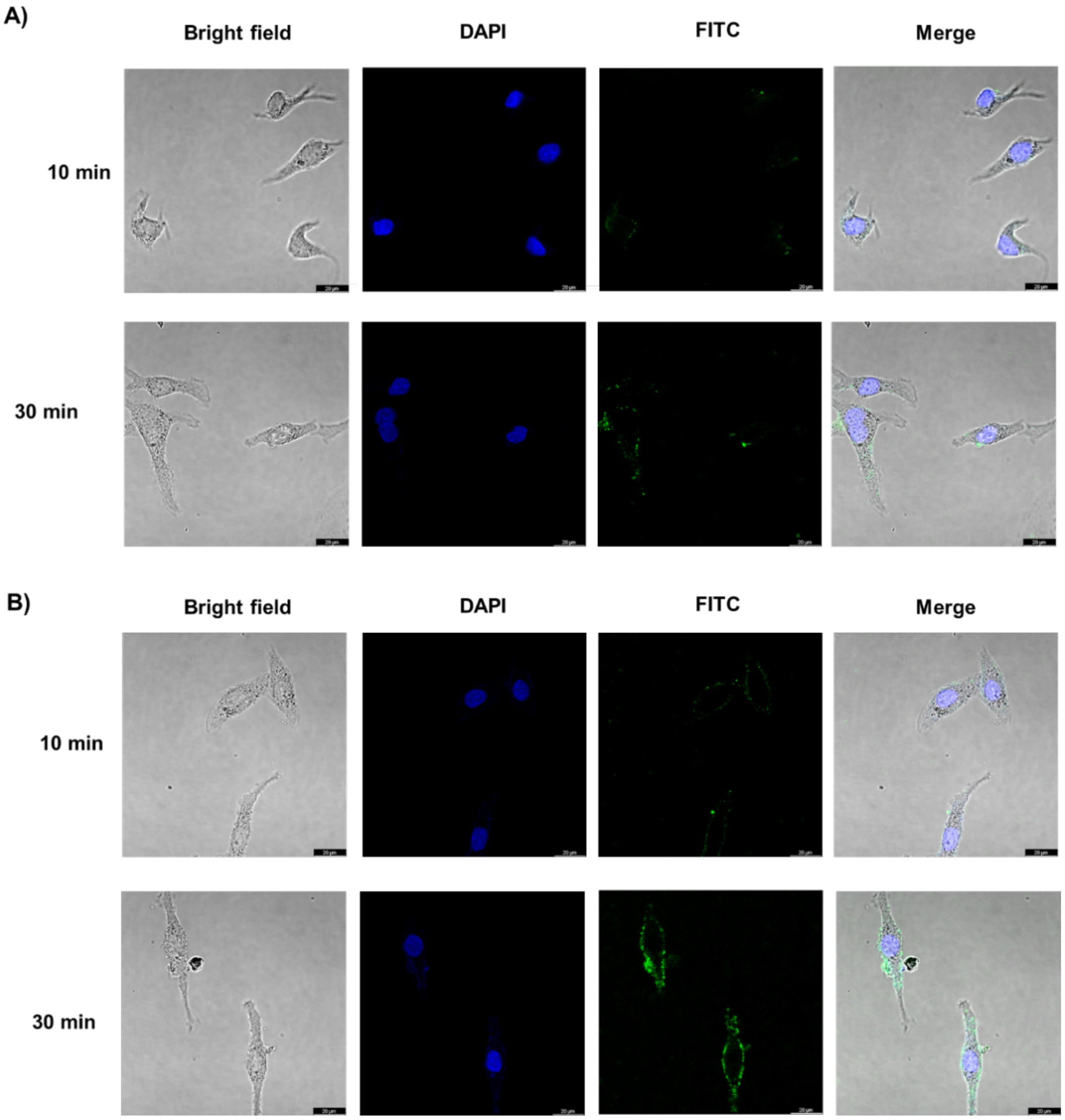

Figure S27. Confocal images of liposome-cell fusion. (**A**) Confocal images of fusion between normal liposome and SKOV3 cells. (**B**) Confocal images of fusion between immunoliposome and SKOV3 cells.

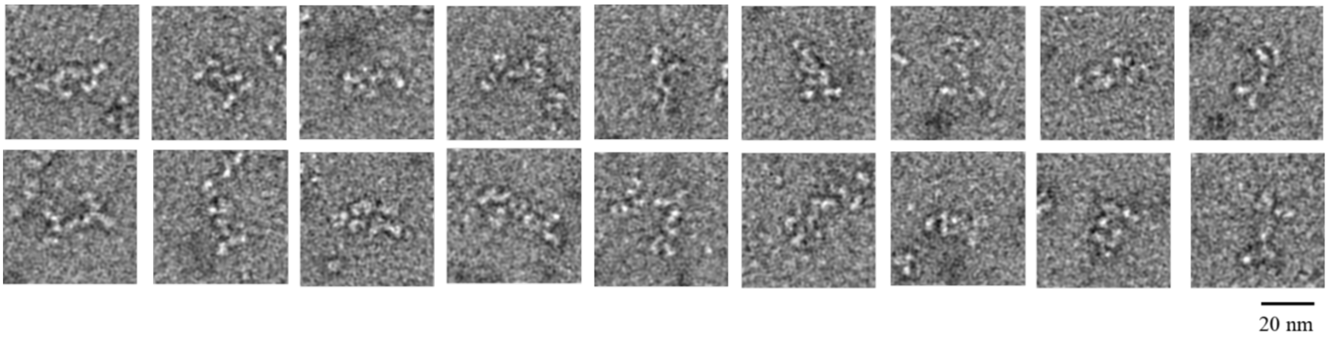

Figure S28. Representative EM images of the IgG dimers. Box size, 240 px. Box width, 74.4 nm.

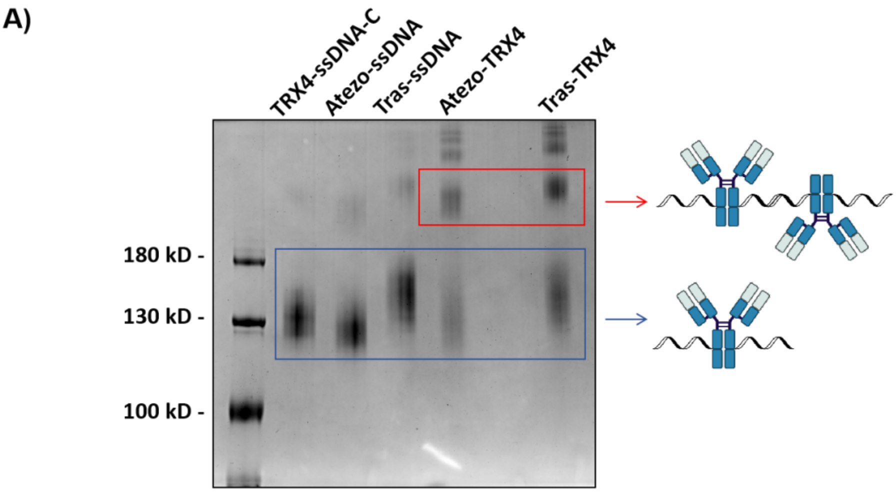

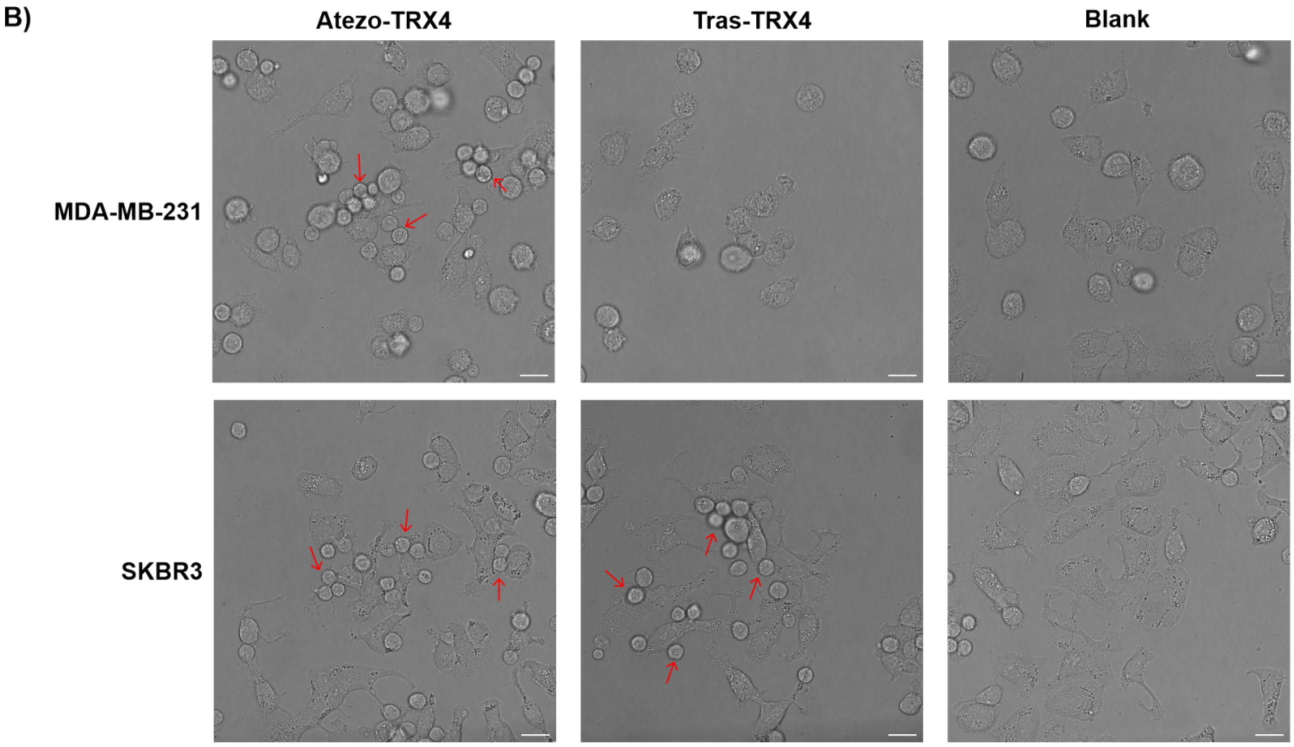

Figure S29. Bispecific antibody and Jurkat cells recruitment. (**A)** Non-reduced SDS-PAGE analysis of Atezo-TRX4 and Tras-TRX4 bispecific antibody. (**B**) Confocal images of the binding of Jurkat cells and MDA-MB-231 (PDL1+/HER2-) or SKBR3 (PDL1+/HER2+) cells via Atezo-TRX4 (anti-PDL1/CD3) or Tras-TRX4 (anti-HER2/CD3) bispecific antibodies. Scale Bar: 20 µm.

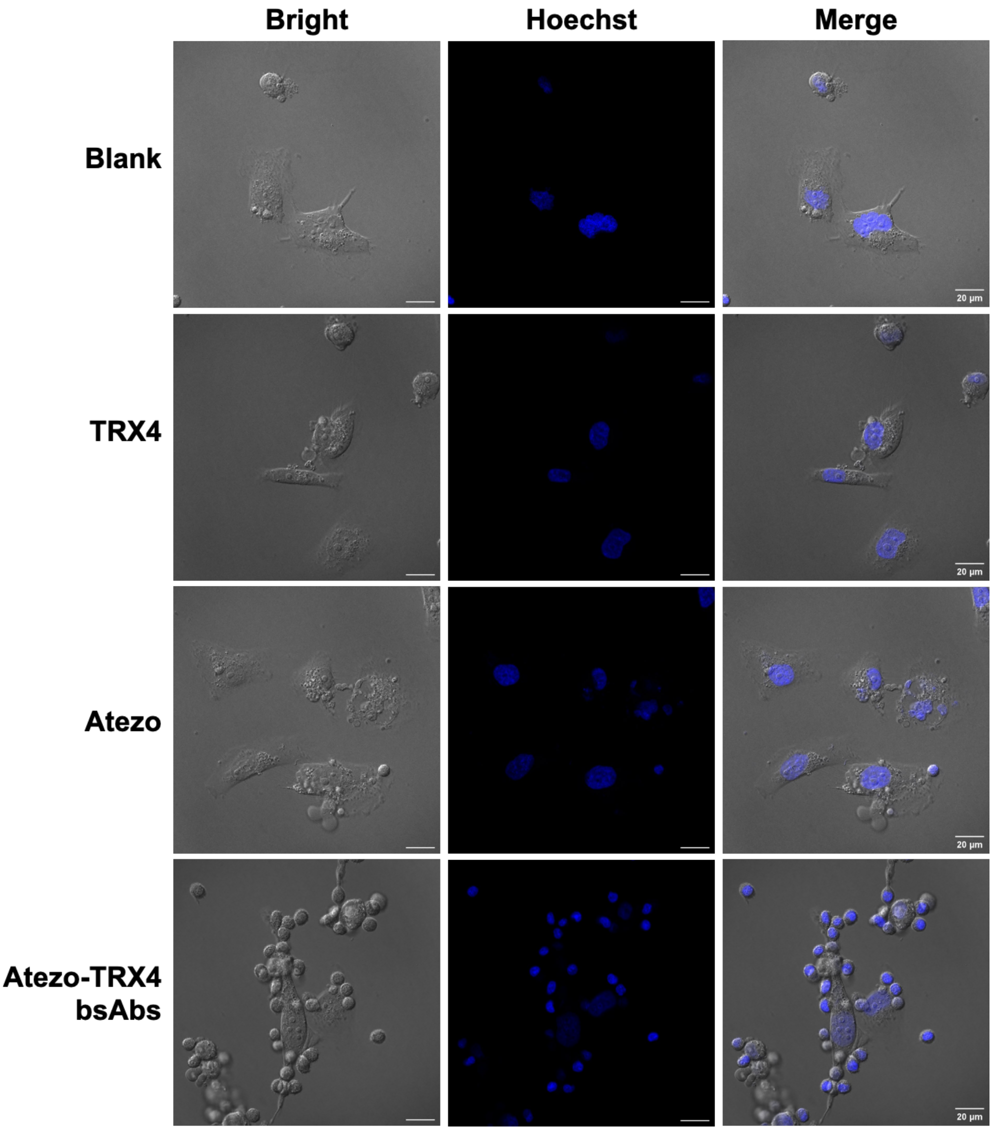

Figure S30. Confocal images show that the Atezo-TRX4 heterodimer engages T cells to MDA-MB-231 cells. T cells were amplified from human PBMCs. The cell cultures were stained with Hoechst 33342 to show the position of intact nuclei. The fluorescence of Hoechst 33342 was detected using the excitation/emission wavelengths of 361 nm / 486 nm.

More compound characterization Data

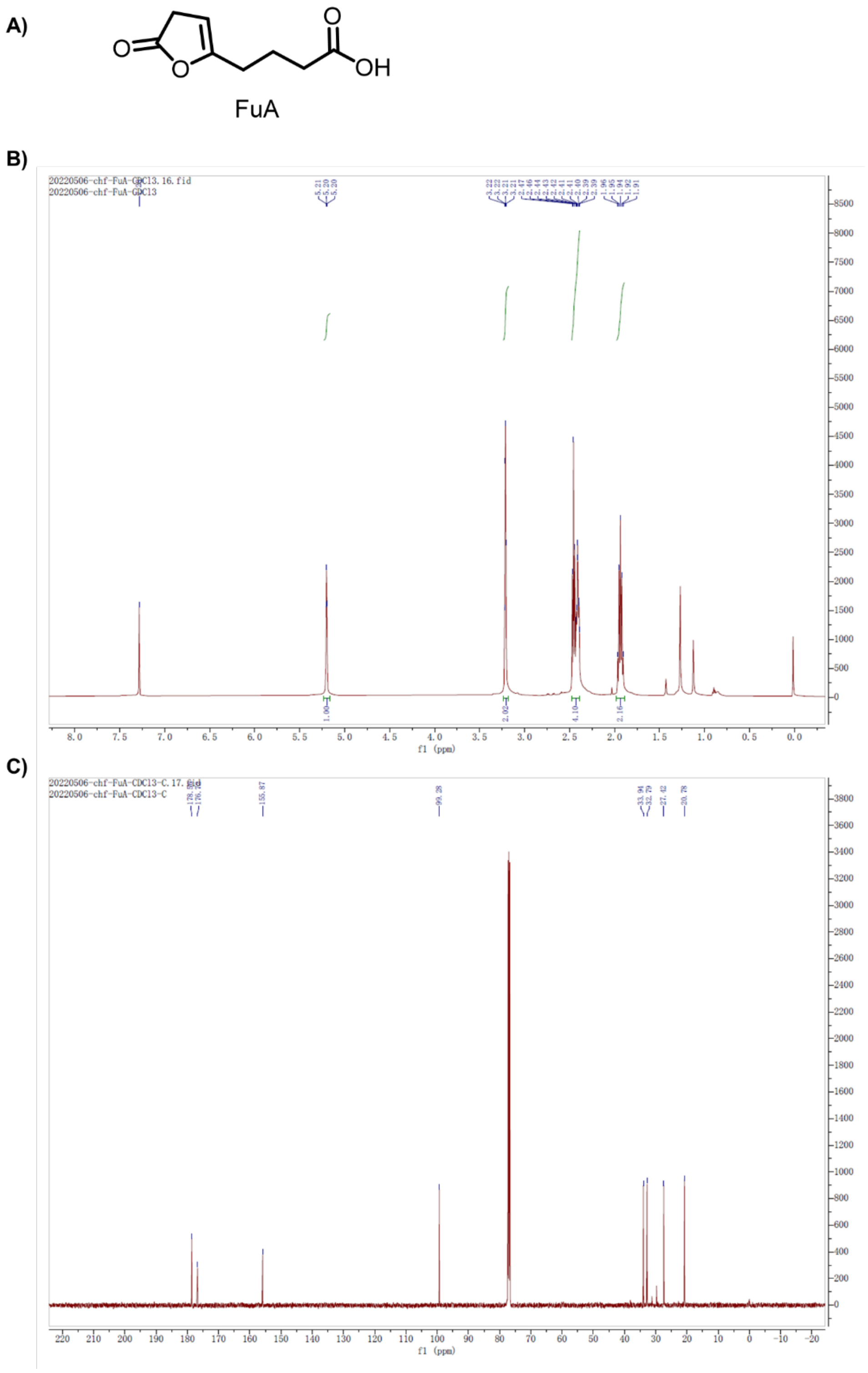

Figure S31. NMR spectrums of **FuA**. (**A**) Structure of FuA. (**B**) H[1]-NMR (500 MHz, Chloroform-d) spectrum of FuA. (**C**) C[13]-NMR (125 MHz, Chloroform-d) spectrum of FuA.

A)

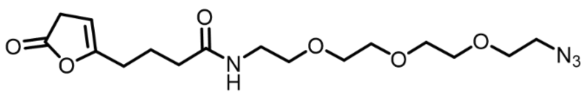

FuA-PEG-N$_3$

B)

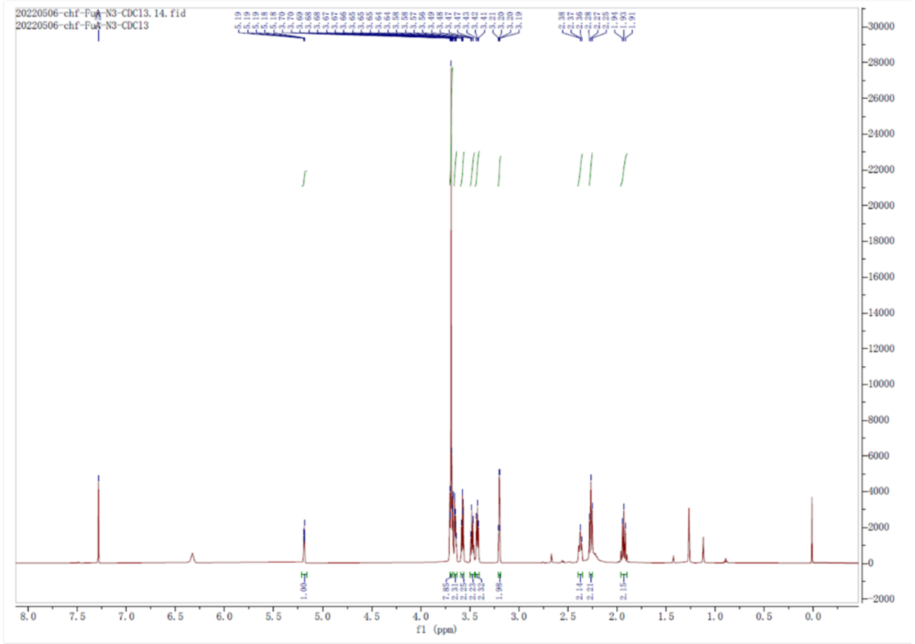

C)

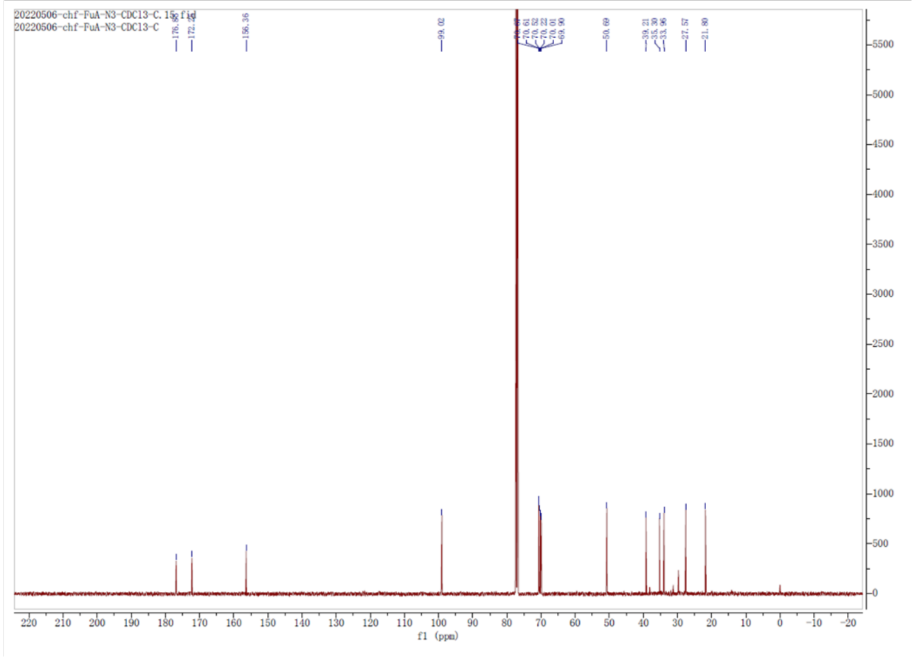

Figure S32. NMR spectrums of **FuA-PEG-N$_3$**. (**A**) Structure of FuA-PEG-N$_3$. (**B**) H[1]-NMR (500 MHz, Chloroform-*d*) spectrum of FuA-PEG-N$_3$. (**C**) C[13]-NMR (125 MHz, Chloroform-*d*) spectrum of FuA-PEG-N$_3$.

A)

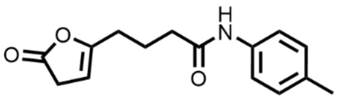

FuA-Phe

B)

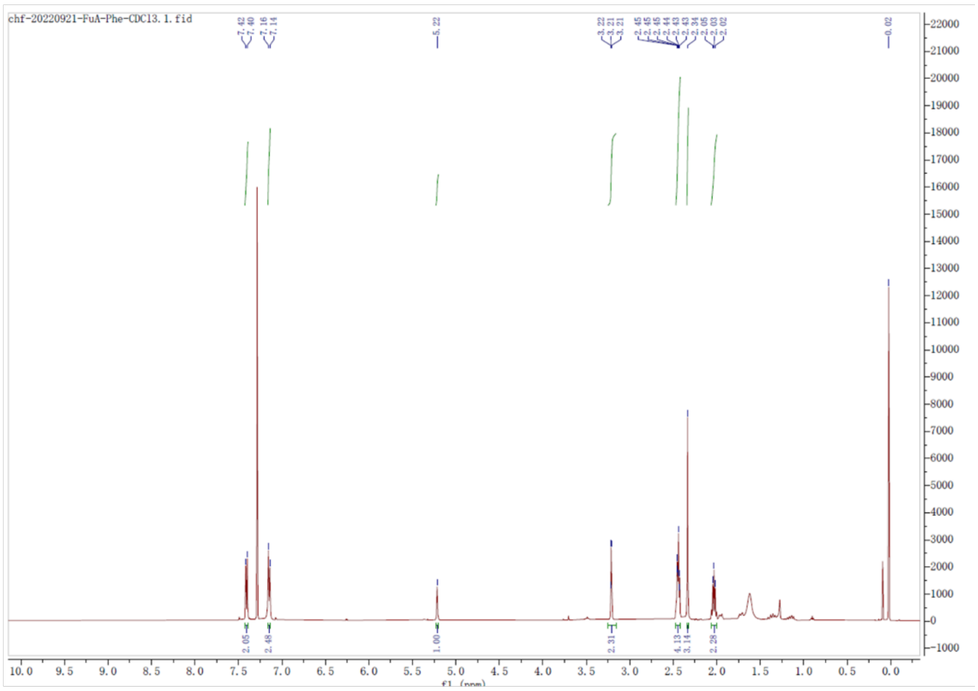

C)

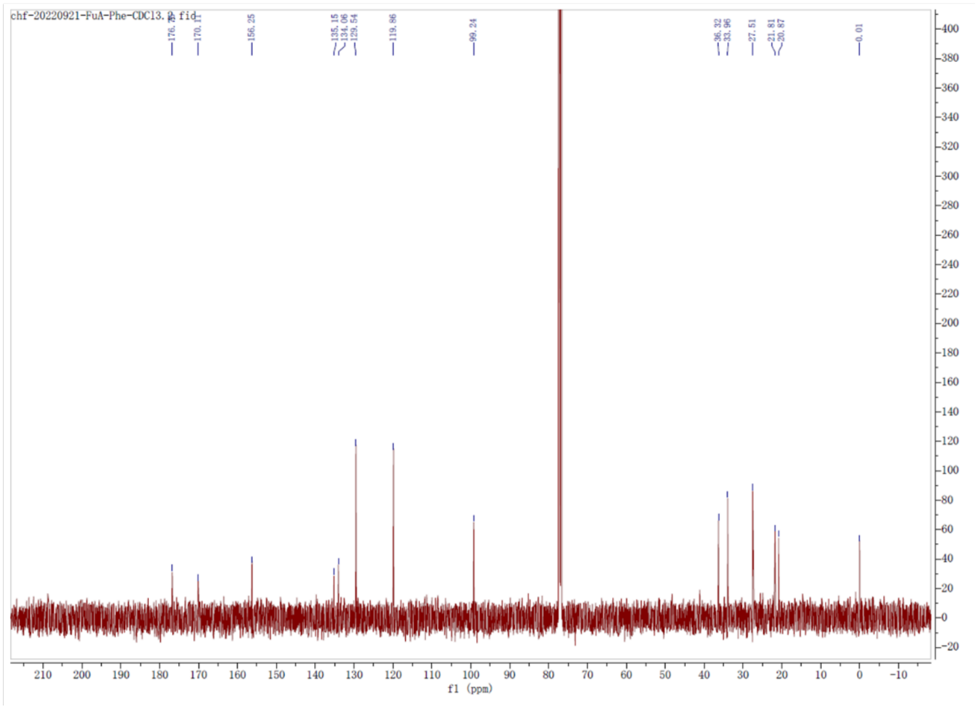

Figure S33. NMR spectrums of **FuA-Phe**. (**A**) Structure of FuA-Phe. (**B**) H[1]-NMR (500 MHz, Chloroform-*d*) spectrum of FuA-Phe. (**C**) C[13]-NMR (125 MHz, Chloroform-*d*) spectrum of FuA-Phe.

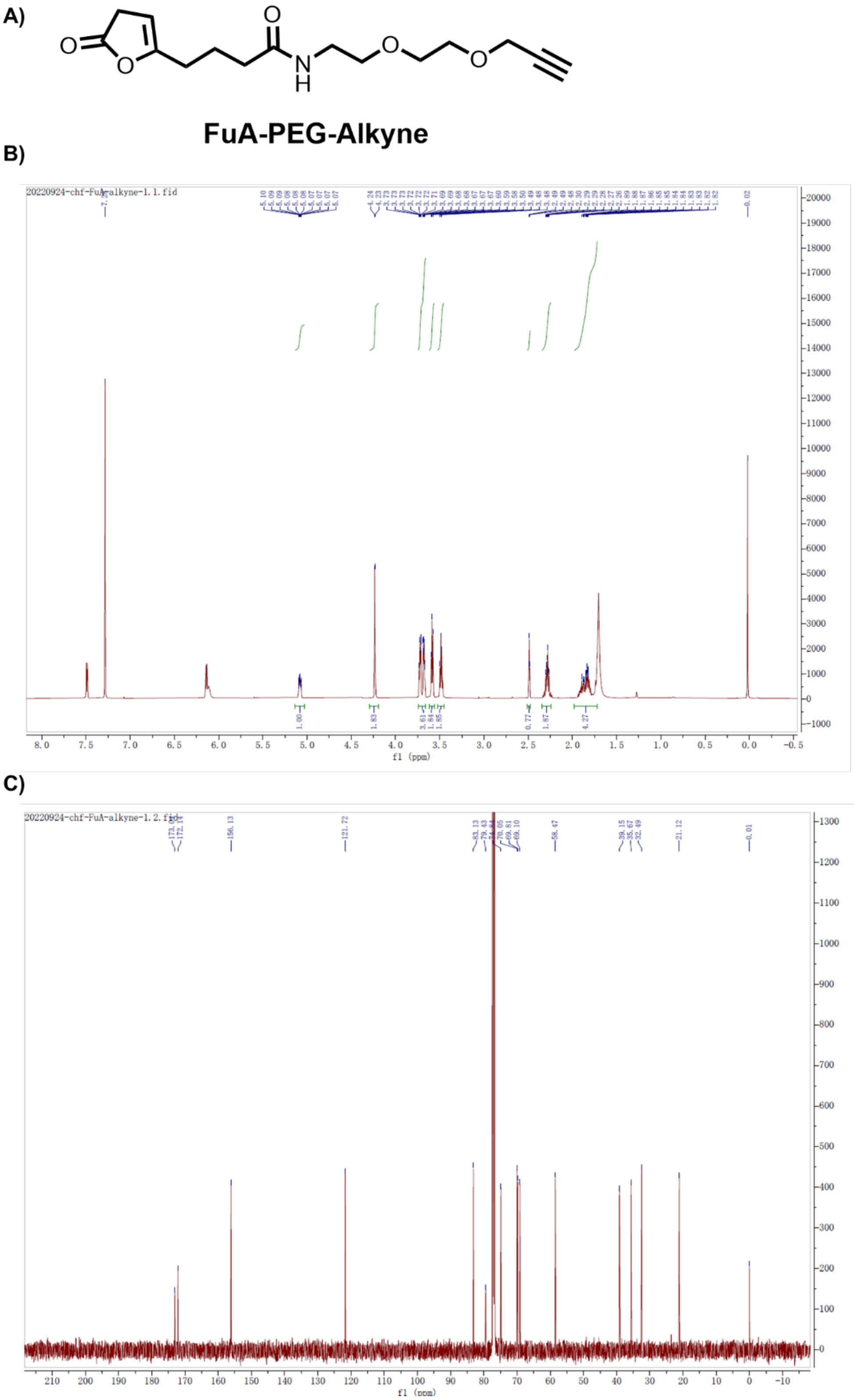

Figure S34. NMR spectrums of **FuA-PEG-Alkyne**. (**A**) Structure of FuA-PEG-Alkyne. (**B**) H^1-NMR (500 MHz, Chloroform-*d*) spectrum of FuA-PEG-Alkyne. (**C**) C^{13}-NMR (125 MHz, Chloroform-*d*) spectrum of FuA-PEG-Alkyne.

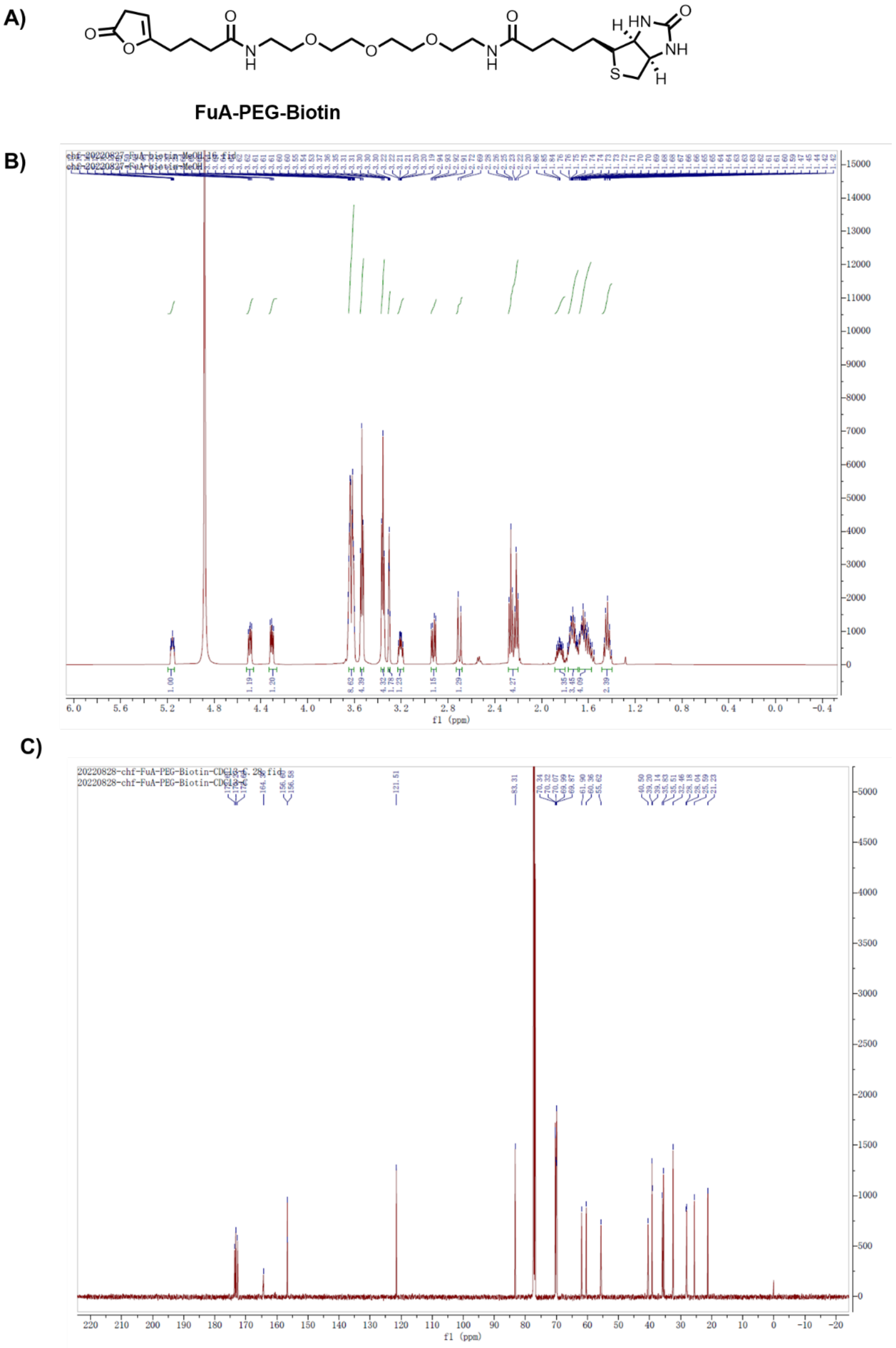

Figure S35. NMR spectrums of **FuA-PEG-Biotin**. (**A**) Structure of FuA-PEG-Biotin. (**B**) H^1-NMR (500 MHz, Methanol-d_4) spectrum of FuA-PEG-Biotin. (**C**) C^{13}-NMR (125 MHz, Chloroform-*d*) spectrum of FuA-PEG-Biotin.

References

1. Xi, Z.; Kong, H.; Chen, Y.; Deng, J.; Xu, W.; Liang, Y.; Zhang, Y., Metal- and strain-free bioorthogonal cycloaddition of o-diones with furan-2(3H)-one as anionic cycloaddend. *Angew. Chem. Int. Ed.* **2022,** *61* (23), e202200239.

2. Chen, H.; Wong, H.-C. F.; Qiu, J.; Li, B.; Yuan, D.; Kong, H.; Bao, Y.; Zhang, Y.; Xu, Z.; Tse, Y.-L. S.; Xia, J. Site-selective tyrosine reaction for antibody-cell conjugation and targeted immunotherapy. *Adv. Sci.* **2024**, *11*, 2305012.

3. *Gaussian 16 Rev. C.01*; Wallingford, CT, 2016.

4. Chai, J.-D.; Head-Gordon, M. Long-range corrected hybrid density functionals with damped atom–atom dispersion corrections. *Physical Chemistry Chemical Physics* **2008**, *10* (44), 6615-6620.

5. Marenich, A. V.; Cramer, C. J.; Truhlar, D. G. Generalized born solvation model SM12. *J. Chem. Theory Comput.* **2013**, *9* (1), 609-620.

6. Bergner, A.; Dolg, M.; Küchle, W.; Stoll, H.; Preuß, H. Ab initio energy-adjusted pseudopotentials for elements of groups 13–17. *Mol. Phys.* **1993**, *80* (6), 1431-1441. Dolg, M.; Wedig, U.; Stoll, H.; Preuss, H. Energy‐adjusted ab initio pseudopotentials for the first row transition elements. *J. Chem. Phys.* **1987**, *86* (2), 866-872. Hariharan, P. C.; Pople, J. A. The influence of polarization functions on molecular orbital hydrogenation energies. *Theor. Chim. Acta* **1973**, *28*, 213-222.

7. Li, X.; Frisch, M. J. Energy-represented direct inversion in the iterative subspace within a hybrid geometry optimization method. *J. Chem. Theory Comput.* **2006**, *2* (3), 835-839.

8. Fukui, K. The path of chemical reactions-the IRC approach. *Acc. Chem. Res.* **1981**, *14* (12), 363-368.

9. Weigend, F.; Furche, F.; Ahlrichs, R. Gaussian basis sets of quadruple zeta valence quality for atoms H–Kr. *J. Chem. Phys.* **2003**, *119* (24), 12753-12762.

10. Cramer, C. J. *Essentials of computational chemistry: theories and models*; John Wiley & Sons, 2013.
